# Cobalt-assisted route to rare carbocyclic *C*-ribonucleosides†

**DOI:** 10.1039/d3ra04937j

**Published:** 2023-10-20

**Authors:** A. C. Ojeda-Porras, V. Roy, O. Bourzikat, P. Favetta, L. A. Agrofoglio

**Affiliations:** a Université d'Orléans et CNRS, ICOA, UMR 7311 F-45067 Orléans France luigi.agrofoglio@univ-orleans.fr

## Abstract

(Re)emerging RNA viruses have been major threats to public health in the past years, and from the few drugs available, nucleoside analogues are still at the cornerstone of the antiviral therapy. Among them, the synthesis of carbocyclic *C*-nucleosides is suffering from long syntheses and poor yields. Herein we report a concise stereoselective synthesis of rare carbocyclic *C*-nucleosides (11a–l) bearing non-canonical nucleobases through a cobalt-assisted-route as key step starting from the optically pure (−)-cyclopentenone 1. This approach paves the route for novel carbocyclic *C*-nucleoside discovery.

## Abstract 2

Twelve rare carbocyclic *C*-nucleosides bearing non-canonical nucleobases were obtained by a cobalt assisted-route starting from the optically pure (−)-cyclopentenone.

## Graphical abstract

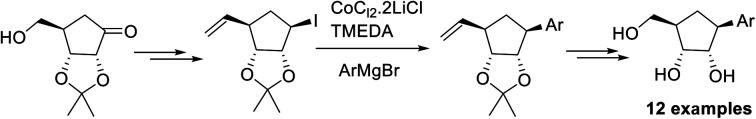

## Introduction

**Fig. 1:**
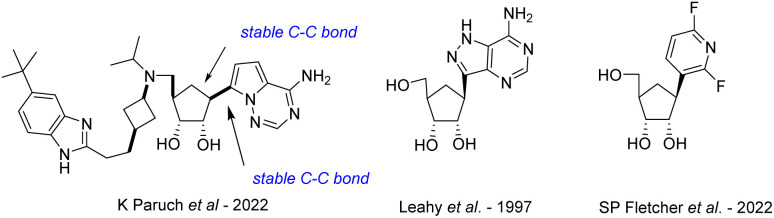
Structure of synthetic carbocyclic *C*-nucleosides.

The continuous growth of the human population as well as human interaction with wild environments have shown several emerging and re-emerging RNA viruses responsible of highly fatal viral diseases and threatened public health with deadly outbreaks of global concern,^1–4^ the majority of RNA viruses are zoonotic or vector-borne infectious agents^5–7^ and are considered as the most common class of pathogens (2 to 3 novel viruses discovered by year);^8^ they represent a challenge for global disease control^9^ because of their faster evolutionary rates and increased mutability compared to some DNA viruses.^10^ Except for the SARS-CoV-2 for which vaccines have been developed at an unprecedented speed, the biological diversity and rapid adaptive rates of RNA viruses make that no effective commercial vaccine is available for any of the above-mentioned viruses, and we are still awaiting the first effective antiviral drugs to be approved. Nucleoside analogues, with more than 40 derivatives marketed to treat viral DNA infections as well as certain cancers or bacterial infections,^11^ are the most promising and leading-class of antivirals against RNA emerging viruses.^12^ On one hand, the replacement of the oxygen in the furanose ring with methylene group led to carbocyclic nucleosides which are chemically more stable towards the enzymatic cleavage of the *N*-glycosidic bond and have an increased cell permeability because of its higher lipophilicity than their nucleoside parents. The broad spectrum of biological activities of carbocyclic nucleosides and their pharmaceutical importance have generated considerable research interest in their design and synthesis. Many reviews by us^13^ and others^14^ have been published on this topic, as well as on their biological applications.^15^ On the other hand, *C*-nucleosides,^16^ in which the ribofuranosyl moiety is linked to a heterocyclic non-canonical base (such as bearing hydrophobic aryl group)^17^ through a C–C bond instead of C–N-bond found in natural nucleosides, have been reported for their antiviral and/or anticancer potential. So far, no natural carbocyclic *C*-nucleosides have been found in nature and only a few have been synthesized to date (Fig. 1), such as the inhibitor of histone methyltransferase DOT1L recently developed by Paruch *et al.*,^18^ the carbocyclic formycin^19^ analog by Leahy *et al.*, and the compound reported by Fletcher *et al.*^20^ bearing a difluoro pyridine non-canonical nucleobase.

**Scheme 1:**
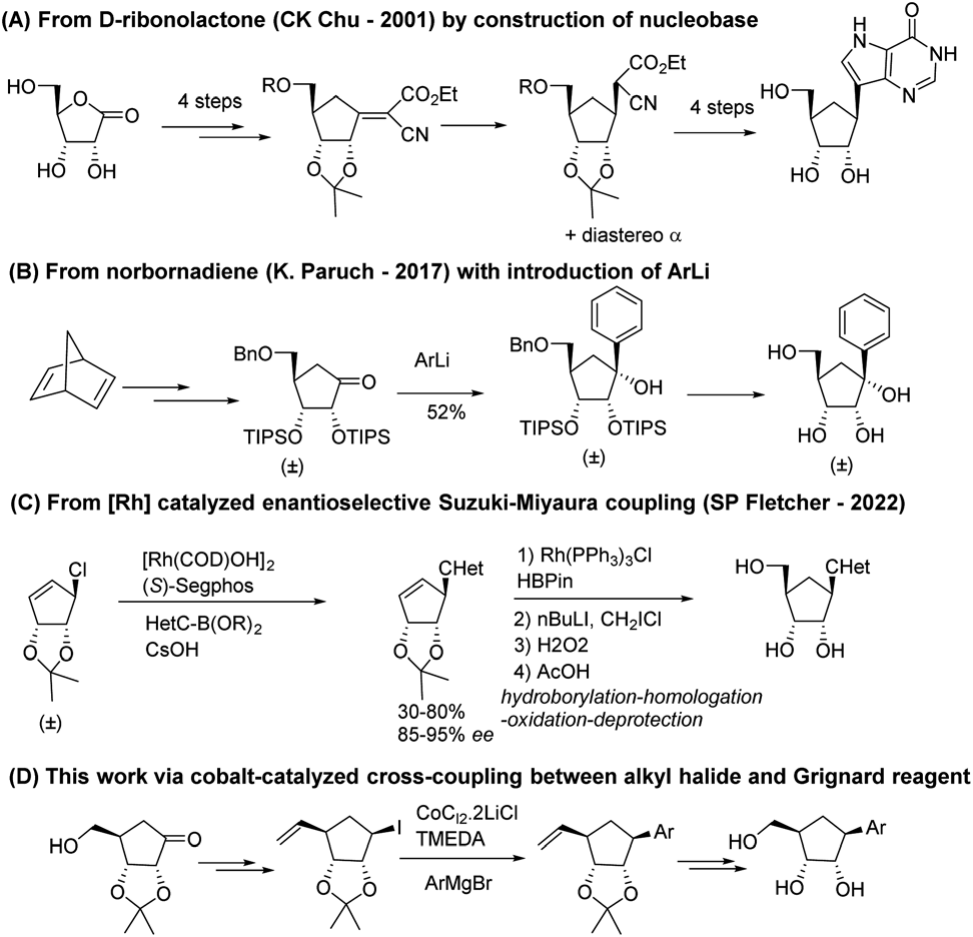
Comparison of previous syntheses and our synthesis of carbocyclic *C*-ribonucleosides.

A first approach was reported in 2001 by Chu and co-workers^21^ from 1,4-γ-ribonolactone as chiral starting material (Scheme 1A). The key intermediate is obtained by Bu_3_SnH–AIBN reduction of an olefinic intermediate obtained by treating the protected ribonolactone with ethyl cyanoacetate and *t*BuOK and then it is converted to final carbocyclic *C*-nucleosides by the full construction of the pyrazole non-canonical nucleobases.

Paruch and co-workers developed a diastereoselective, racemic procedure from norbornadiene through the TIPS-protected cyclopentanone which underwent diastereoselective addition of various lithiated (hetero)aryl moieties (Scheme 1B).^22^ A recent publication by Fletcher group^20^ reported the Rh-catalytic asymmetric synthesis of carbocyclic *C*-nucleosides using an asymmetric Suzuki–Miyaura type reaction as the key C–C bond forming step, followed by late-stage addition of the hydroxymethyl group to ribose analogs (Scheme 1C).

Even if several efforts towards the synthesis of enantio-enriched carbocyclic nucleosides have been reported in the past years, the multi-step sequences combined with the difficulty associated to the installation of stereocenters make these approaches long, tedious, and not suitable for the pharmaceutical industry. Thus, the synthetic difficulties have probably hampered the production of those very promising carbocyclic *C*-nucleoside analogs.

Hence, we focused on designing a new, versatile, robust and stereo-controlled synthetic route to diverse carbocyclic *C*-nucleosides without the use chiral catalyst but taking advantage of stereocenters previously set in the molecule (Scheme 1D). Several non-canonical aromatic bases were installed.^23^ This (hetero)aryl moieties included electron-donating and electron-withdrawing substituents, and the presence of heteroatoms in the ring, while maintaining π-stacking and Watson–Crick hydrogen bond interactions.

## Results and discussion

**Scheme 2:**
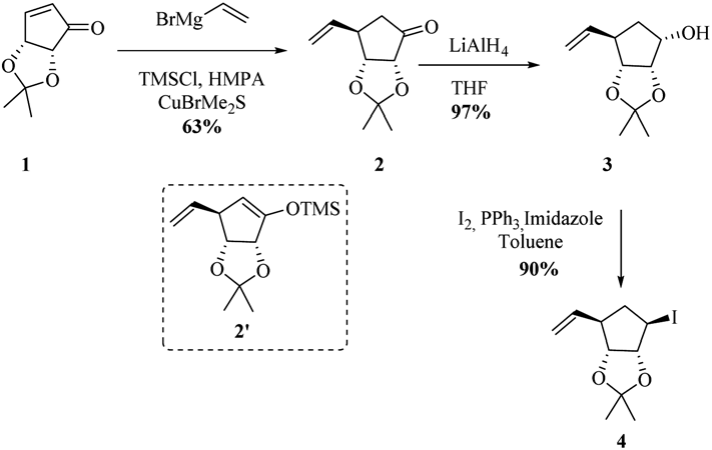
Synthesis of enantiomerically pure halohydrin 4.

We began the synthesis from the commercially available optically pure (−)-cyclopentenone 1 which is easily obtained in 3 steps from d-ribose.^24^ Following a reported procedure by Schneller *et al.*,^25*a*^ the diastereoselective Grignard addition of vinyl magnesium^25*b*^ bromide on 1 afforded the ketone 2 as one diastereomer in 63% yield (Scheme 2). ^1^H NMR and NOESY experiments revealed that H_3_ correlates only with H_2_ (d, *J*(H_3_,H_2_) = 5.2 Hz) and not with H_4_ corresponding to a dihedral angle close to 90° according to Karplus equation. This angle is attainable with H_3_ and H_4_*trans* to each other (but not when they are *cis*), proving that the vinyl substituent of the organocuprate added to the less hindered face resulting in 2 with the depicted stereochemistry, as illustrated by molecular models (see ESI†). The use of TBAF in the quenching of the Grignard addition step is vital since the yield of 2 can be negatively affected by the formation and isolation of silyl ether 2′. Reduction of resulting cyclopentanone 2 with AlLiH_4_ afforded alcohol 3 in 97% yield. The compound was diastereomerically pure according to ^1^H and ^13^C NMR. ^1^H NMR and NOESY analysis, and Karplus equation indicate that compound 3 has H_1_ and H_2_ in a *cis* relationship.^25*b*^ This is in agreement with molecular models (see ESI†). With alcohol 3 in hand, an Appel-type reaction (proceeding with the inversion of configuration) using iodine and triphenylphosphine in the presence of imidazole was intended. This reaction was initially performed using dichloromethane as solvent, but unfortunately, just starting material was recovered. Finally when toluene was used as solvent and the reaction was done under reflux conditions, the desired iodohydrin 4 was obtained in 90% yield. Interestingly, ^1^H NMR clearly shows a van der Waals deshielding effect of the iodine atom on the vinylic C–H proton.

**Scheme 3:**
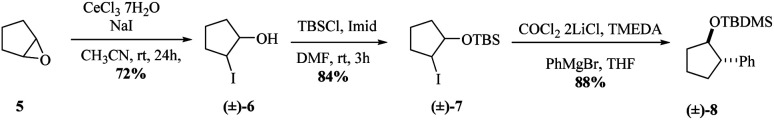
Cobalt-assisted cross-coupling on a model compound.

Inspired by the cobalt-mediated diastereoselective cross coupling reaction reported by Knochel and co-workers,^26^ we decided to apply this methodology as key step of the synthesis of carbocyclic *C*-nucleosides. According to the authors, the presence of the neighbouring TBS-ether group is responsible for the high diastereoselectivity observed because the silyl ether and the newly inserted aromatic group adopt an anti-configuration. We sought the isopropylidene motive at C2–C3 would have a similar effect by blocking the α-phase of the cyclopentane nucleoside. The cobalt solution can be prepared in a day using the protocol reported by Knochel. Before trying the conditions in the previously synthesized iodohydrin 4, we decided to test them on the model substrate 7 (Scheme 3). Starting from epoxide 5, iodohydrin 6 was obtained in 72% yield. Further protection of the alcohol as a TBS ether afforded the model substrate 7 in 84% yield as a racemate.

With 7 in hand, we tested the cobalt-catalysed cross coupling conditions. Thus, to a cooled solution of the iodohydrin 7 and TMEDA in dry THF was slowly added the Grignard reagent. To our delight, we were able to isolate the desired compound 8 in 88% yield as only relative *trans* stereochemistry, when the reaction was allowed to stir at room temperature overnight. The aryl group was *trans* to the silylated ether. We did notice that shorter reaction times resulted in a decreased reaction yield.

**Scheme 4:**
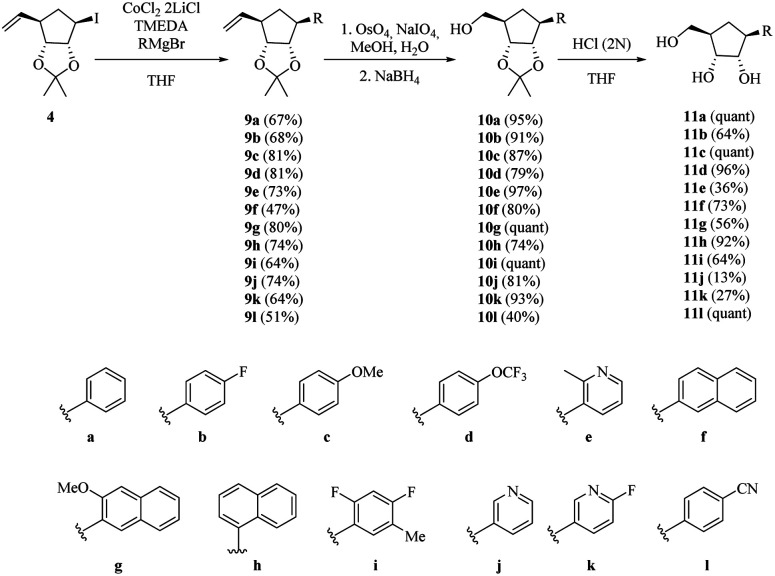
Synthesis of carbocyclic *C*-nucleosides 11a–l.

We then applied the above-mentioned conditions for the cross-coupling reaction between halohydrin 4 and different Grignard reagents (Scheme 4). The desired products (9a–l) were obtained in good yields in the presence of activating groups, such as methoxide 9c and trifluoro-methoxide 9d. Fluorinated compound 9b was isolated in 68% yield. Ortho-substituted product 9e was successfully obtained in moderate yield despite its steric hindrance and the presence of a nitrogenated base.

**Scheme 5:**
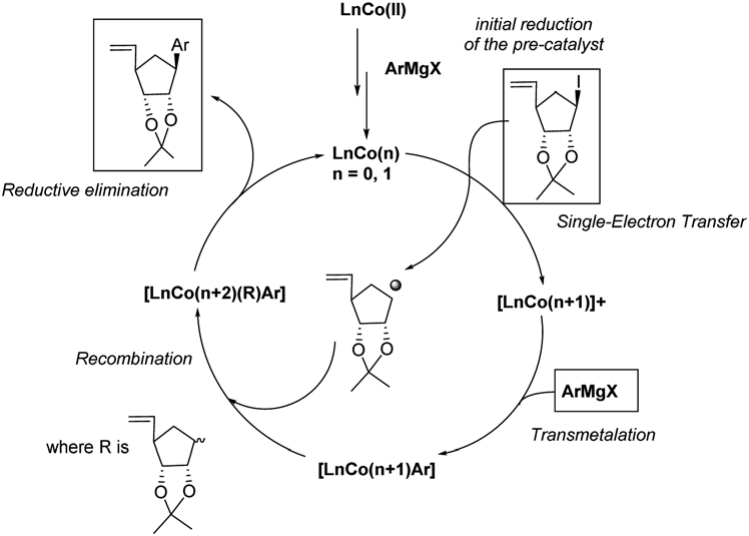
Proposed mechanism (adapted from Guérinot and Cossy^27^).

Binaphthyl substituted products 9f–h were also were also isolated, although in poor to moderate yields. Only one isomer is produced and its formation proceeds through a proposed radical mechanism (Scheme 5).^27^

With alkenes 9a–l in hand, an oxidative cleavage of the double bond was attempted. Initially, a ruthenium catalysed reaction was performed on 9a furnishing 10a in just 25% yield. To our delight, the use of osmium tetroxide afforded alcohols 10a–l in excellent yields (Scheme 4). Later deprotection of the isopropylidene ketal in acidic media yielded the desired carbocyclic *C*-nucleoside analogues 11a–l. It should be noticed that the ^1^H and ^13^C NMR spectra of 11a are consistent with those reported by Fletcher *et al.* for the same compound obtained by a different way.^20^

## Conclusions

In summary, a series of hitherto unknown rare carbocyclic *C*-nucleosides (11a–l) were successfully synthesized in a highly efficient manner from protected ribonolactone 1 through a cobalt-catalyzed cross-coupling between the enantiopure halohydrine 4 and various aryl Grignard reagents. To the best of our knowledge, the proposed synthetic procedure is far superior to any other route reported to date. It is believed that this study will contribute to explore this promising class of carbocyclic *C*-nucleosides.

## Experimental section

### General methods

Commercially available chemicals were of reagent grade and used as received. The reactions were monitored by thin layer chromatography (TLC) analysis using silica gel plates (Kieselgel 60F254, E. Merck). Column chromatography was performed on Silica Gel 60 M (0.040 × 0.063 mm, E. Merck). Optical rotations were measured with a PerkinElmer 341 polarimeter in appropriate solvent, at the indicated temperature (20 °C) and at 589 nm sodium line, in a 1 dm cell. Concentrations are given in g/100 mL. The ^1^H and ^13^C NMR spectra were recorded on a Varian InovaUnity 400 spectrometer (400 MHz) in (d4) methanol, CDCl_3_, shift values in parts per million relative to SiMe_4_ as internal reference. High Resolution Mass spectra were performed on a Bruker maXis mass spectrometer by the “Federation de Recherche” ICOA/CBM (FR2708) platform.

### Preparation of the CoCl_2_·2LiCl (1.0 M) solution in THF

The CoCl_2_·2LiCl (1.0 M) solution in THF was prepared following the reported experimental procedure.^24^ A dried two neck 50 mL flask equipped with a stirring bar a septum was charged with anhydrous LiCl (2.12 g) and heated at 130 °C under high vacuum for 2 h. After cooling to room temperature under argon, CoCl_2_ (3.24 g) was added. The resulting mixture was heated to 130 °C for 5 h under high vacuum, cooled down to room temperature under argon and charged with dry THF (25 mL). The resulting solution was vigorously stirred at room temperature overnight. The dark blue solution was used within 1 month.

### Synthesis of Grignard reagents

All non-commercially available aryl Grignard reagents were prepared following the following experimental procedure. To a flask containing a stirring bar was added Mg turnings (240 mg, 10 mmol, 2.0 equiv.) and LiCl (230 mg, 5.5 mmol, 1.1 equiv.). The flask was flame dried under vacuum and flushed with argon three times. THF (5 mL) and dibromoethane (25 μL) were then added, followed by the dropwise addition of 1-bromo-4-methoxybenzene (935 mg, 5 mmol). The resulting solution was stirred at room temperature for two hours and used immediately in the cross coupling reaction.

### General procedure A: cobalt mediated cross coupling reaction

To a stirred solution of iodohydrin (1.0 equiv.) in dry THF (1 M) was added TMEDA (0.3 equiv.) and a 1.0 M solution of CoCl_2_·2LiCl in THF (0.85 equiv.). The resulting blue solution was cooled down to −78 °C before the Grignard reagent (1.7 equiv.) was slowly added. The reaction mixture was stirred at room temperature overnight. A saturated aqueous solution of NH_4_Cl was added to quench the reaction. The aqueous layer was extracted with AcOEt, and the combined organic extracts were washed with brine, dried over anhydrous MgSO_4_, filtered and concentrated under reduced pressure. The crude mixture was then purified by flash chromatography to afford the desired product.

### General procedure B: oxidative cleavage of double bond catalysed by osmium

To a stirred solution of alkene (1.0 equiv.) in a mixture of methanol: water (7 : 3) (0.11 M) was added NaIO_4_ (2.07 equiv.). The reaction mixture was cooled down to 0 °C and a 2.5 wt% solution of OsO_4_ in *t*-BuOH (0.08 equiv.) was slowly added. The resulting mixture was stirred 1 h at 0 °C and 2 h at room temperature. The newly formed white solid was removed by filtration and washed with a mixture of methanol water (2 : 1). The filtrate was then cooled down to 0 °C and NaBH_4_ (6.4 equiv.) was added portion wise, turning the reaction black. The resulting mixture was allowed to stir at room temperature for 30 minutes, after which the volatiles were removed under reduced pressure. Brine was then added and the aqueous layer was extracted with CH_2_Cl_2_. The combined organic extracts were washed with brine, dried over anhydrous MgSO_4_, filtered and concentrated under reduced pressure afford the desired product.

### General procedure C: deprotection of the isopropylidene acetal

To a stirred solution of isopropylidene acetal (1.0 equiv.) in THF (0.4 M) at 0 °C was added dropwise a 2.0 M aqueous solution of HCl (0.2 M final concentration). The reaction mixture was stirred at room temperature for 16 h. The resulting solution was neutralized by the addition of a saturated aqueous solution of NaHCO_3_. The aqueous layer was abundantly extracted with AcOEt, and the combined organic extracts were washed with brine, dried over anhydrous MgSO_4_, filtered and concentrated under reduced pressure to afford the desired product.

#### (3a*R*,6a*R*)-2,2-Dimethyl-3a,6a-dihydro-4*H*-cyclopenta[*d*][1,3]dioxol-4-one (1)

Commercially available ketone 8 was purified by flash chromatography using petroleum ether : AcOEt to afford the desired product 1 as a white solid (109 mg, 84% yield). ^1^H NMR (400 MHz, CDCl_3_) *δ* ppm: 7.57–7.63 (m, 1H), 6.21 (d, *J* = 5.9 Hz, 1H), 5.34–5.18 (m, 1H), 4.46 (d, *J* = 5.5 Hz, 1H), 1.41 (s, 6H). ^13^C NMR (100 MHz, CDCl_3_) *δ* ppm: 202.9, 159.5, 134.3, 115.6, 78.6, 76.5, 27.4, 26.2.

#### (3a*R*,6*R*,6a*R*)-2,2-Dimethyl-6-vinyltetrahydro-4*H*-cyclopenta[*d*][1,3]dioxol-4-one (2)

To a stirred suspension of CuBrS·Me_2_S (612 mg, 2.98 mmol, 0.1 equiv.) in dry THF (13.5 mL) at −78 °C was added a solution of vinyl magnesium bromide (1.0 M in THF) (90 mL, 90 mmol, 3.0 equiv.) dropwise. The resulting mixture was allowed to stir at this temperature for 10 minutes before a solution of ketone 1 (4.59 g, 29.8 mmol), TMSCl (7.75 mL, 60.8 mmol, 2.05 equiv.) and HMPA (13.5 mL, 77.5 mmol, 2.6 equiv.) in dry THF (14.8 mL) was slowly added. The reaction mixture was stirred at −78 °C for 5 h. A saturated aqueous solution of NH_4_Cl and 10 mL of TBAF were added to quench the reaction. The resulting mixture was allowed to stir at room temperature for 30 min. The aqueous layer was extracted with AcOEt, and the combined organic extracts were washed with brine, dried over anhydrous MgSO_4_, filtered and concentrated under reduced pressure. The crude mixture was then purified by flash chromatography using petroleum ether : AcOEt (5 : 1) to afford the desired product 2 as a pale yellow oil (3.54 g, 65% yield).^25*a*^ [*α*]^20^_D_ = −95.1 (*c* = 1.2, CH_3_OH). ^1^H NMR (400 MHz, CDCl_3_) *δ* ppm: 5.83 (ddd, *J* = 17.2, 10.6, 6.5 Hz, 1H), 5.20–5.02 (m, 2H), 4.64 (d, *J* = 5.2 Hz, 1H), 4.20 (d, *J* = 5.2 Hz, 1H), 3.11 (t, *J* = 7.3 Hz, 1H), 2.83 (dd, *J* = 18.3, 8.6 Hz, 1H), 2.28 (d, *J* = 18.3 Hz, 1H), 1.44 (s, 3H), 1.35 (s, 3H). ^13^C NMR (100 MHz, CDCl_3_) *δ* ppm: 213.2, 137.1, 116.4, 112.4, 81.4, 77.9, 39.8, 38.6, 26.9, 24.9.

#### (3a*S*,4*S*,6*R*,6a*R*)-2,2-Dimethyl-6-vinyltetrahydro-4*H*-cyclopenta[*d*][1,3]dioxol-4-ol (3)

To a stirred suspension of LiAlH_4_ (1.23 g, 32.2 mmol, 1.7 equiv.) in dry THF (48 mL) at −78 °C was slowly added a solution of ketone 2 (3.46 g, 18.9 mmol) in dry THF (32 mL). The reaction mixture was stirred at room temperature for 2 h. A saturated aqueous solution of Rochelle salt was slowly added to quench the reaction and the resulting mixture was allowed to stir at room temperature overnight. The aqueous layer was extracted with AcOEt, and the combined organic extracts were washed with brine, dried over anhydrous MgSO_4_, filtered and concentrated under reduced pressure to afford the desired product 3 as a colourless oil (3.40 g, 97% yield).^25*a*^ [*α*]^20^_D_ = +44.4 (*c* = 1.20, CH_3_OH). ^1^H NMR (400 MHz, CDCl_3_) *δ* ppm: 5.74 (ddd, *J* = 17.2, 10.5, 6.5 Hz, 1H), 5.10–5.04 (m, 2H), 4.51–4.45 (m, 2H), 4.07 (bs, 1H), 2.78–2.69 (m, 1H), 2.42 (d, *J* = 7.5 Hz, 1H D_2_O exchangeable), 1.95–1.84 (m, 2H), 1.51 (s, 3H), 1.35 (s, 3H). ^13^C NMR (100 MHz, CDCl_3_) *δ* ppm: 138.0, 115.3, 111.6, 84.3, 79.0, 71.1, 44.3, 36.0, 26.1, 24.3.

#### (3a*R*,4*R*,6*R*,6a*R*)-4-Iodo-2,2-dimethyl-6-vinyltetrahydro-4*H*-cyclopenta[*d*][1,3]dioxole (4)

To a stirred solution of alcohol 3 (3.40 g, 18.5 mmol) in toluene (645 mL) was added triphenyl phosphine (14.5 g 55.4 mmol, 3.0 equiv.), imidazole (3.77 g, 55.4 mmol, 3.0 equiv.) and iodine (9.37 g, 36.9 mmol, 2.0 equiv.). The reaction mixture was refluxed for 2 h and then cooled down to room temperature. The resulting mixture was diluted with AcOEt and washed with a saturated aqueous solution of NaHCO_3_ The organic phase was washed with brine, dried over anhydrous MgSO_4_, filtered and concentrated under reduced pressure. The crude mixture was then purified by flash chromatography using petroleum ether : CH_2_Cl_2_ (8 : 2) to (1 : 1) to afford the desired product 4 as a pale yellow solid (4.67 g, 86% yield). [*α*]^20^_D_ = −2.7 (*c* = 0.60, CH_3_OH). ^1^H NMR (400 MHz, CDCl_3_) *δ* ppm: 5.97 (ddd, *J* = 17.2, 10.3, 6.9 Hz, 1H), 5.15–5.05 (m, 2H), 4.97–4.81 (m, 1H), 4.41 (dd, *J* = 6.7, 4.0 Hz, 1H), 4.11–3.98 (m, 1H), 2.77–2.57 (m, 2H), 2.05–2.13 (m, 1H), 1.50 (s, 3H), 1.30 (s, 3H). ^13^C NMR (100 MHz, CDCl_3_) *δ* ppm: 138.1, 115.8, 113.2, 90.5, 85.4, 49.9, 43.2, 27.3, 24.9, 24.8. HRMS (ESI): calcd for C_10_H_16_IO_2_^+^ [M + H]^+^, 295.0189; found, 295.0190.

#### 2-Iodocyclopentan-1-ol (6)

To a stirred solution of CeCl_3_·7H_2_O(1.07 g, 2.89 mmol, 0.5 equiv.) and NaI (865 mg, 5.75 mmol, 1.0 equiv.) in acetonitrile (57 mL) was added dropwise a solution of epoxide 5 (483 mg, 5.75 mmol) in acetonitrile (11 mL). The reaction mixture was protected from the light and stirred at room temperature for 24 h. The volatiles were removed under reduced pressure and the resulting mixture was diluted with AcOEt. The organic phase was washed with brine, dried over anhydrous MgSO_4_, filtered and concentrated under reduced pressure. The crude mixture was then purified by flash chromatography using CH_2_Cl_2_ to afford the desired product 6 as a pale yellow oil (876 mg, 72% yield). ^1^H NMR (400 MHz, CDCl_3_) *δ* ppm: 4.46 (bs, 1H), 4.06–4.02 (m, 1H), 2.41–2.32 (m, 1H), 2.14–2.05 (m, 2H), 1.96 (s, 1H), 1.88–1.77 (m, 2H), 1.63–1.58 (m, 1H). ^13^C NMR (100 MHz, CDCl_3_) *δ* ppm: 82.2, 35.6, 34.1, 31.0, 22.0.

#### (±)-*tert*-Butyl((2-iodocyclopentyl)oxy)dimethylsilane (7)

To a stirred solution of alcohol 6 (87 mg, 0.41 mmol) in dry DMF (2.0 mL) was added imidazole (130 mg, 1.62 mmol, 3.9 equiv.) and TBDMSCl (185 mg, 1.35 mmol, 3 equiv.). The reaction mixture was stirred at room temperature for 3 h and 30 min. The resulting mixture was diluted with CH_2_Cl_2_ and the organic phase was washed several times with an aqueous solution of LiCl, then washed with brine, dried over anhydrous MgSO_4_, filtered and concentrated under reduced pressure. The crude mixture was then purified by flash chromatography using petroleum ether to afford the desired product 7 as a pale yellow oil (109 mg, 84% yield). ^1^H NMR (400 MHz, CDCl_3_) *δ* ppm: 4.46–4.37 (m, 1H), 4.07–3.95 (m, 1H), 2.25–2.41 (m, 1H), 1.99–2.12 (m, 2H), 1.85–1.72 (m, 2H), 1.61–1.47 (m, 1H), 0.88 (s, 9H), 0.09 (s, 3H), 0.06 (s, 3H). ^13^C NMR (100 MHz, CDCl_3_) *δ* ppm: 82.5, 35.8, 35.0, 32.3, 25.8, 22.3, 18.0, −4.5, −4.8.

#### (±)-*tert*-Butyldimethyl((2-phenylcyclopentyl)oxy)silane (8)

Following the general procedure A for iodohydrin 7 (163 mg, 0.5 mmol), the desired compound 8 (122 mg, 88% yield) was isolated as a colourless oil after purification by flash chromatography using petroleum ether. ^1^H NMR (400 MHz, CDCl_3_) *δ* ppm: 7.38–7.15 (m, 5H), 4.07 (q, *J* = 7.0 Hz, 1H), 2.92 (dd, *J* = 16.6, 8.1 Hz, 1H), 2.20–1.98 (m, 2H), 1.95–1.75 (m, 3H), 1.74–1.64 (m, 1H), 0.83 (s, 9H), −0.14 (s, *J* = 19.0 Hz, 3H), −0.19 (s, 3H). ^13^C NMR (100 MHz, CDCl_3_) *δ* ppm: 144.0, 128.1, 127.6, 126.0, 81.6, 54.4, 34.8, 30.9, 25.8, 21.9, 18.0, −5.0.

#### (3a*S*,4*S*,6*R*,6a*R*)-2,2-Dimethyl-4-phenyl-6-vinyltetrahydro-4*H*-cyclopenta[*d*][1,3]dioxole (9a)

Following the general procedure A for iodohydrin 4 (700 mg, 2.35 mmol), the desired compound 9a (360 mg, 63% yield) was isolated as a colourless oil after purification by flash chromatography using petroleum ether : CH_2_Cl_2_ (7 : 3) to (1 : 1). [*α*]^20^_D_ = −8.4 (*c* = 15.3, CH_3_OH). ^1^H NMR (400 MHz, CDCl_3_) *δ* ppm: 7.37–7.23 (m, 5H), 5.96 (ddd, *J* = 17.3, 10.3, 7.1 Hz, 1H), 5.28–5.10 (m, 2H), 4.61–4.57 (m, 1H), 4.49–4.43 (m, 1H), 3.27 (dt, *J* = 12.6, 6.3 Hz, 1H), 2.81 (td, *J* = 12.7, 6.3 Hz, 1H), 2.36 (dt, *J* = 12.7, 6.3 Hz, 1H), 1.87–1.76 (m, 1H), 1.61 (s, 3H), 1.35 (s, 3H). ^13^C NMR (100 MHz, CDCl_3_) *δ* ppm: 142.3, 139.0, 128.5, 127.1, 126.5, 115.2, 113.4, 87.0, 85.3, 50.5, 49.5, 38.4, 27.7, 25.2. HRMS (ESI): calcd for C_16_H_21_O_2_^+^ [M + H]^+^, 245.1536; found, 245.1535.

#### (3a*S*,4*S*,6*R*,6a*R*)-4-(4-Fluorophenyl)-2,2-dimethyl-6-vinyltetrahydro-4*H*-cyclopenta[*d*][1,3]dioxole (9b)

Following the general procedure A for iodohydrin 4 (231 mg, 0.78 mmol), the desired compound 9b (135 mg, 68% yield) was isolated as a colourless oil after purification by flash chromatography using petroleum ether : CH_2_Cl_2_ (1 : 2). [*α*]^20^_D_ = −22.9 (*c* = 5.10, CH_3_OH). ^1^H NMR (400 MHz, CDCl_3_) *δ* ppm: 7.29–7.23 (m, 2H), 6.99–7.06 (m, 2H), 6.02–5.87 (m, 1H), 5.20 (d, *J* = 17.2 Hz, 1H), 5.11 (d, *J* = 10.4 Hz, 1H), 4.54–4.40 (m, 2H), 3.31–3.16 (m, 1H), 2.81–2.76 (m, 1H), 2.36–2.31 (m, 1H), 1.83–1.69 (m, 1H), 1.60 (s, 3H), 1.34 (s, 3H). ^13^C NMR (100 MHz, CDCl_3_) *δ* ppm: 161.6 (d, *J* = 245 Hz), 138.8, 137.9 (d, *J* = 3.03 Hz), 128.5 (d, *J* = 7.07 Hz), 115.3, 115.2 (d, *J* = 21.2), 113.5, 87.0, 85.2, 49.9, 49.3, 38.4, 27.6, 25.1. ^19^F NMR (376 MHz, CDCl_3_) *δ* ppm: −116.69. HRMS (ESI): calcd for C_16_H_20_FO_2_^+^ [M + H]^+^, 263.1442; found, 263.1443.

#### (3a*S*,4*S*,6*R*,6a*R*)-4-(4-Methoxyphenyl)-2,2-dimethyl-6-vinyltetrahydro-4*H*-cyclopenta[*d*][1,3]dioxole (9c)

Following the general procedure A for iodohydrin 4 (294 mg, 1.0 mmol), the desired compound 9c (222 mg, 81% yield) was isolated as a white solid after purification by flash chromatography using petroleum ether : CH_2_Cl_2_ (6 : 4) to (4 : 6). ^1^H NMR (400 MHz, CDCl_3_) *δ* ppm: 7.19 (d, *J* = 8.5 Hz, 2H), 6.86 (d, *J* = 8.4 Hz, 2H), 5.92 (ddd, *J* = 17.3, 10.3, 7.1 Hz, 1H), 5.19–5.07 (m, 2H), 4.52–4.49 (m, 1H), 4.43–4.39 (m, 1H), 3.79 (s, 3H), 3.19 (dt, *J* = 12.6, 6.2 Hz, 1H), 2.81–2.69 (m, 1H), 2.35–2.25 (m, 1H), 1.79–1.71 (m, 1H), 1.57 (s, 3H), 1.31 (s, 3H). ^13^C NMR (100 MHz, CDCl_3_) *δ* ppm: 158.3, 139.0, 134.4, 128.0, 115.1, 113.9, 113.3, 87.2, 85.3, 55.3, 49.7, 49.5, 38.6, 27.7, 25.1. HRMS (ESI): calcd for C_17_H_23_O_3_^+^ [M + H]^+^, 275.1645; found, 275.1642. [*α*]^20^_D_ = 18.23 (*c* 1.0, CH_2_Cl_2_).

#### (3a*S*,4*S*,6*R*,6a*R*)-2,2-Dimethyl-4-(4-(trifluoromethoxy)phenyl)-6-vinyltetrahydro-4*H*-cyclopenta[*d*][1,3]dioxole (9d)

Following the general procedure A for iodohydrin 4 (294 mg, 1.0 mmol), the desired compound 9d (265 mg, 81% yield) was isolated as a white solid after purification by flash chromatography using petroleum ether : CH_2_Cl_2_ (7 : 3) to (1 : 1). [*α*]^20^_D_ = −43.5 (*c* = 1.40, CH_3_OH). ^1^H NMR (400 MHz, CDCl_3_) *δ* ppm: 7.40–7.14 (m, 4H), 5.95 (ddd, *J* = 17.3, 10.3, 7.2 Hz, 1H), 5.20 (d, *J* = 17.8 Hz, 1H), 5.12 (d, *J* = 10.3 Hz, 1H), 4.44–4.58 (m, 2H), 3.25 (dt, *J* = 12.7, 6.3 Hz, 1H), 2.83–2.78 (m, 1H), 2.38–2.33 (m, 1H), 1.87–1.70 (m, 1H), 1.60 (s, 3H), 1.34 (s, 3H). ^13^C NMR (100 MHz, CDCl_3_) *δ* ppm: 147.8 (q, *J* = 147 Hz), 141.0, 138.7, 128.4, 121.1, 120.5 (q, *J* = 258 Hz), 115.3, 113.6, 86.9, 85.2, 50.0, 49.3, 38.2, 27.6, 25.1. ^19^F NMR (376 MHz, CDCl_3_) *δ* ppm: −57.95. HRMS (ESI): calcd for C_17_H_20_F_3_O_3_^+^ [M + H]^+^, 329.1359; found, 329.1361.

#### 3-((3a*S*,4*S*,6*R*,6a*R*)-2,2-Dimethyl-6-vinyltetrahydro-4*H*-cyclopenta[*d*][1,3]dioxol-4-yl)-2-methylpyridine (9e)

Following the general procedure A for iodohydrin 4 (294 mg, 1.0 mmol), the desired compound 9e (190 mg, 73% yield) was isolated as a colourless oil after purification by flash chromatography using petroleum ether : AcOEt : Et_3_N (9 : 1 : 0.1) to (8 : 2 : 0.1). [*α*]^20^_D_ = −3.7 (*c* = 5.30, CH_3_OH). ^1^H NMR (400 MHz, CDCl_3_) *δ* ppm: 8.35 (d, *J* = 4.6 Hz, 1H), 7.47 (d, *J* = 7.8 Hz, 1H), 7.14–7.07 (m, 1H), 5.91 (ddd, *J* = 17.1, 9.7, 7.4 Hz, 1H), 5.17 (d, *J* = 17.2 Hz, 1H), 5.09 (d, *J* = 10.3 Hz, 1H), 4.56–5.50 (m, 1H), 4.47–4.42 (m, 1H), 3.45 (dt, *J* = 12.3, 6.1 Hz, 1H), 2.84–2.78 (m, 1H), 2.65 (s, 3H), 2.29–2.21 (m, 1H), 1.79–1.67 (m, 1H), 1.57 (s, 3H), 1.29 (s, 3H). ^13^C NMR (100 MHz, CDCl_3_) *δ* ppm: 157.2, 146.9, 138.6, 135.6, 133.3, 121.3, 115.4, 113.4, 86.8, 85.2, 49.6, 46.4, 38.2, 27.7, 25.2, 22.9. HRMS (ESI): calcd for C_16_H_22_NO_2_^+^ [M + H]^+^, 260.1645; found, 260.1649.

#### (3a*S*,4*S*,6*R*,6a*R*)-2,2-Dimethyl-4-(naphthalen-2-yl)-6-vinyltetrahydro-4*H*-cyclopenta[*d*][1,3]dioxole (9f)

Following the general procedure A for iodohydrin 4 (294 mg, 1.0 mmol), the desired compound 9f (138 mg, 47% yield) was isolated as a colourless oil after purification by flash chromatography using petroleum ether : CH_2_Cl_2_ (7 : 3) to (3 : 7). [*α*]^20^_D_ = −20.3 (*c* = 1.00, CH_3_OH). ^1^H NMR (400 MHz, CDCl_3_) *δ* ppm: 7.83–7.79 (m, 3H), 7.70 (s, 1H), 7.48–7.42 (m, 3H), 6.04–5.89 (m, 1H), 5.21 (d, *J* = 17.2 Hz, 1H), 5.11 (d, *J* = 10.3 Hz, 1H), 4.68–4.62 (m, 1H), 4.53–4.44 (m, *J* = 6.8 Hz, 1H), 3.41 (dt, *J* = 12.6, 6.2 Hz, 1H), 2.86–2.79 (m, 1H), 2.46–2.39 (m, 1H), 1.97–1.87 (m, 1H), 1.61 (s, 3H), 1.34 (s, 3H). ^13^C NMR (100 MHz, CDCl_3_) *δ* ppm: 139.7, 139.0, 133.5, 132.4, 128.2, 127.7, 127.6, 126.1, 125.8, 125.5, 125.3, 115.3, 113.5, 86.9, 85.4, 50.7, 49.6, 38.4, 27.7, 25.2. HRMS (ESI): calcd for C_20_H_23_O_2_^+^ [M + H]^+^, 295.1693; found, 295.1686.

#### (3a*S*,4*S*,6*R*,6a*R*)-4-(3-Methoxynaphthalen-2-yl)-2,2-dimethyl-6-vinyltetrahydro-4*H*-cyclopenta[*d*][1,3]dioxole (9g)

Following the general procedure A for iodohydrin 4 (294 mg, 1.0 mmol), the desired compound 9g (260 mg, 80% yield) was isolated as a white solid after purification by flash chromatography using petroleum ether : CH_2_Cl_2_ (6 : 4) to (4 : 6). ^1^H NMR (400 MHz, CDCl_3_) *δ* ppm: 7.78 (t, *J* = 8.1 Hz, 2H), 7.72 (s, 1H), 7.47 (t, *J* = 7.5 Hz, 1H), 7.39 (t, *J* = 7.4 Hz, 1H), 7.17 (s, 1H), 6.01 (ddd, *J* = 17.3, 10.2, 7.3 Hz, 1H), 5.25 (d, *J* = 17.2 Hz, 1H), 5.15 (d, *J* = 10.3 Hz, 1H), 4.97 (t, *J* = 5.9 Hz, 1H), 4.58 (t, *J* = 6.8 Hz, 1H), 3.98 (s, 3H), 3.68 (dt, *J* = 11.7, 5.8 Hz, 1H), 2.98–2.81 (m, 1H), 2.48–2.29 (m, 1H), 2.01 (q, *J* = 12.3 Hz, 1H), 1.67 (s, 3H), 1.41 (s, 3H). ^13^C NMR (100 MHz, CDCl_3_) *δ* ppm: 156.2, 139.3, 133.6, 132.1, 128.8, 127.6, 127.3, 126.3, 125.9, 123.8, 115.0, 112.8, 105.6, 85.7, 85.4, 55.3, 50.4, 47.0, 38.4, 28.0, 25.5. HRMS (ESI): calcd for C_21_H_25_O_3_^+^ [M + H]^+^, 325.1798; found, 325.1804.

#### (3a*S*,4*S*,6*R*,6a*R*)-2,2-Dimethyl-4-(naphthalen-1-yl)-6-vinyltetrahydro-4*H*-cyclopenta[*d*][1,3]dioxole (9h)

Following the general procedure A for iodohydrin 4 (294 mg, 1.0 mmol), the desired compound 9h (219 mg, 74% yield) was isolated as a colourless oil after purification by flash chromatography using petroleum ether : CH_2_Cl_2_ (7 : 3) to (1 : 1). ^1^H NMR (400 MHz, CDCl_3_) *δ* ppm: 8.40 (d, *J* = 8.5 Hz, 1H), 7.93 (d, *J* = 8.0 Hz, 1H), 7.82 (d, *J* = 7.6 Hz, 1H), 7.66–7.46 (m, 4H), 6.03 (ddd, *J* = 17.3, 10.3, 7.1 Hz, 1H), 5.29 (d, *J* = 17.2 Hz, 1H), 5.19 (d, *J* = 10.3 Hz, 1H), 4.84–4.72 (m, 1H), 4.57 (t, *J* = 6.8 Hz, 1H), 4.20–4.05 (m, 1H), 3.10–2.89 (m, 1H), 2.56–2.38 (m, 1H), 2.10 (q, *J* = 12.1 Hz, 1H), 1.74 (s, 3H), 1.39 (s, 3H). ^13^C NMR (100 MHz, CDCl_3_) *δ* ppm: 139.1, 138.4, 134.1, 132.3, 128.8, 127.3, 126.1, 125.7, 125.4, 124.2, 122.9, 115.4, 113.1, 86.7, 85.4, 49.9, 46.1, 38.2, 27.9, 25.3. HRMS (ESI): calcd for C_20_H_23_O_2_^+^ [M + H]^+^, 295.1693; found, 295.1694.

#### (3a*S*,4*S*,6*R*,6a*R*)-4-(2,4-Difluoro-5-methylphenyl)-2,2-dimethyl-6-vinyltetrahydro-4*H*-cyclopenta[*d*][1,3]dioxole (9i)

Following the general procedure A for iodohydrin 4 (220 mg, 0.75 mmol), the desired compound 9i (98 mg, 45% yield, 64% yield brsm) was isolated as a colourless oil after purification by flash chromatography using petroleum ether : CH_2_Cl_2_ (8 : 2) to (6 : 4). ^1^H NMR (400 MHz, CDCl_3_) *δ* ppm: 7.05 (t, *J* = 8.4 Hz, 1H), 6.76 (t, *J* = 10.0 Hz, 1H), 5.93 (ddd, *J* = 17.3, 10.3, 7.1 Hz, 1H), 5.18 (d, *J* = 17.2 Hz, 1H), 5.10 (d, *J* = 10.3 Hz, 1H), 4.69 (t, *J* = 6.5 Hz, 1H), 4.47 (t, *J* = 6.8 Hz, 1H), 3.33 (dt, *J* = 12.5, 6.3 Hz, 1H), 2.78 (dq, *J* = 13.0, 6.6 Hz, 1H), 2.35–2.17 (m, 4H), 1.83 (q, *J* = 12.6 Hz, 1H), 1.58 (s, 3H), 1.33 (s, 3H). ^13^C NMR (100 MHz, CDCl_3_) *δ* ppm: 160.6 (dd, *J* = 60.9, 11.8 Hz), 158.2 (dd, *J* = 60.4, 11.8 Hz), 138.7, 130.9 (t, *J* = 6.4 Hz), 124.2 (dd, *J* = 14.1, 4.1 Hz), 120.4 (dd, *J* = 17.1, 3.9 Hz), 115.3, 113.3, 103.6 (t, *J* = 26.3 Hz), 85.6 (d, *J* = 1.3 Hz), 85.2, 49.7, 45.3, 38.2 (d, *J* = 2.2 Hz), 27.7, 25.2, 13.9 (d, *J* = 3.0 Hz). ^19^F NMR (376 MHz, CDCl_3_) *δ* ppm: −116.16 to −116.38 (m), −117.60 (dd, *J* = 16.9, 8.4 Hz). HRMS (ESI): calcd for C_17_H_21_F_2_O_2_^+^ [M + H]^+^, 295.1504; found, 295.1505.

#### 3-((3a*S*,4*S*,6*R*,6a*R*)-2,2-Dimethyl-6-vinyltetrahydro-4*H*-cyclopenta[*d*][1,3]dioxol-4-yl)pyridine (9j)

Following the general procedure A for iodohydrin 4 (220 mg, 0.75 mmol), the desired compound 9j (136 mg, 74% yield) was isolated as a colourless oil after purification by flash chromatography using petroleum ether : AcOEt : Et_3_N (9 : 1 : 0.1) to (8 : 2 : 0.1). ^1^H NMR (400 MHz, CDCl_3_) *δ* ppm: 8.66–8.38 (m, 2H), 7.58 (d, *J* = 7.8 Hz, 1H), 7.31–7.12 (m, 1H), 5.91 (ddd, *J* = 17.3, 10.2, 7.1 Hz, 1H), 5.13 (dd, *J* = 33.0, 13.8 Hz, 2H), 4.53 (t, *J* = 6.9 Hz, 1H), 4.44 (t, *J* = 6.7 Hz, 1H), 3.22 (dt, *J* = 12.7, 6.3 Hz, 1H), 2.89–2.70 (m, 1H), 2.45–2.25 (m, 1H), 1.77 (q, *J* = 12.7 Hz, 1H), 1.56 (s, 3H), 1.31 (s, 3H). ^13^C NMR (100 MHz, CDCl_3_) *δ* ppm: 148.8, 148.0, 138.5, 137.5, 134.6, 123.4, 115.4, 113.7, 86.5, 85.2, 49.3, 48.3, 38.0, 27.6, 25.1. HRMS (ESI): calcd for C_15_H_20_NO_2_^+^ [M + H]^+^, 246.1489; found, 246.1492.

#### 5-((3a*S*,4*S*,6*R*,6a*R*)-2,2-Dimethyl-6-vinyltetrahydro-4*H*-cyclopenta[*d*][1,3]dioxol-4-yl)-2-fluoropyridine (9k)

Following the general procedure A for iodohydrin 4 (220 mg, 0.75 mmol), the desired compound 9k (78 mg, 40% yield, 64% yield brsm) was isolated as a yellow oil after purification by flash chromatography using petroleum ether : AcOEt : Et_3_N (50 : 5 : 0.5) ^1^H NMR (400 MHz, CDCl_3_) *δ* ppm: 8.11 (s, 1H), 7.71 (td, *J* = 8.3, 1.8 Hz, 1H), 6.89 (dd, *J* = 8.3, 2.3 Hz, 1H), 5.92 (ddd, *J* = 17.3, 10.1, 7.2 Hz, 1H), 5.19 (d, *J* = 17.2 Hz, 1H), 5.11 (d, *J* = 10.3 Hz, 1H), 4.55–4.40 (m, 2H), 3.31–3.14 (m, 1H), 2.81 (td, *J* = 12.0, 6.0 Hz, 1H), 2.36 (dt, *J* = 12.6, 6.2 Hz, 1H), 1.76 (q, *J* = 12.6 Hz, 1H), 1.58 (s, 3H), 1.32 (s, 3H). ^13^C NMR (100 MHz, CDCl_3_) *δ* ppm: 162.7 (d, *J* = 237.9 Hz), 145.9 (d, *J* = 14.5 Hz), 139.9 (d, *J* = 7.8 Hz), 138.4, 135.2 (d, *J* = 4.5 Hz), 115.6, 113.8, 109.2 (d, *J* = 37.5 Hz), 86.6, 85.1, 49.2, 47.5, 37.9, 27.6, 25.1. ^19^F NMR (376 MHz, CDCl_3_) *δ* ppm: −71.17. HRMS (ESI): calcd for C_15_H_19_FNO_2_^+^ [M + H]^+^, 264.1394; found, 264.1394.

#### 4-((3a*S*,4*S*,6*R*,6a*R*)-2,2-Dimethyl-6-vinyltetrahydro-4*H*-cyclopenta[*d*][1,3]dioxol-4-yl)benzonitrile (9l)

Following the general procedure A for iodohydrin 4 (147 mg, 0.5 mmol), the desired compound 9l (31 mg, 23% yield) was isolated as a yellow oil after purification by flash chromatography using petroleum ether : CH_2_Cl_2_ (6 : 4) to (0 : 1). ^1^H NMR (400 MHz, CDCl_3_) *δ* ppm: 7.63 (d, *J* = 8.3 Hz, 2H), 7.41 (d, *J* = 8.1 Hz, 2H), 6.01–5.84 (m, 1H), 5.24–5.10 (m, 2H), 4.50 (dt, *J* = 13.3, 7.3 Hz, 2H), 3.29 (dt, *J* = 12.7, 6.3 Hz, 1H), 2.82 (td, *J* = 12.5, 6.3 Hz, 1H), 2.37 (dt, *J* = 12.7, 6.3 Hz, 1H), 1.79 (q, *J* = 12.7 Hz, 1H), 1.59 (s, 3H), 1.34 (s, 3H). ^13^C NMR (100 MHz, CDCl_3_) *δ* ppm: 147.9, 138.6, 132.4, 128.1, 119.0, 115.7, 113.9, 110.6, 86.6, 85.3, 50.8, 49.3, 37.9, 27.7, 25.2. HRMS (ESI): calcd for C_17_H_20_NO_2_^+^ [M + H]^+^, 270.1489; found, 264.1491.

#### ((3a*R*,4*R*,6*S*,6a*S*)-2,2-Dimethyl-6-phenyltetrahydro-4*H*-cyclopenta[*d*][1,3]dioxol-4-yl)methanol (10a)

Following the general procedure B for alkene 9a (566 mg, 2.3 mmol), the desired compound 10a (541 mg, 95% yield) was isolated as a colourless oil. ^1^H NMR (400 MHz, CDCl_3_) *δ* ppm: 7.43–7.18 (m, 5H), 4.65–4.44 (m, 2H), 3.86–3.70 (m, 2H), 3.26 (dt, *J* = 12.5, 6.3 Hz, 1H), 2.48–2.23 (m, 2H), 2.06 (bs, 1H), 1.76–1.68 (m, 1H), 1.60 (s, 3H), 1.35 (s, 3H). ^13^C NMR (100 MHz, CDCl_3_) *δ* ppm: 142.2, 128.5, 127.1, 126.5, 113.3, 87.2, 83.1, 64.4, 50.6, 47.6, 35.0, 27.7, 25.2. HRMS (ESI): calcd for C_15_H_20_NaO_3_^+^ [M + Na]^+^, 271.1305; found, 271.1306.

#### ((3a*R*,4*R*,6*S*,6a*S*)-6-(4-Fluorophenyl)-2,2-dimethyltetrahydro-4*H*-cyclopenta[*d*][1,3]dioxol-4-yl)methanol (10b)

Following the general procedure B for alkene 9b (109 mg, 0.42 mmol), the desired compound 10b (100 mg, 91% yield) was isolated as a colourless oil. ^1^H NMR (400 MHz, CDCl_3_) *δ* ppm: 7.22 (dd, *J* = 7.7, 5.9 Hz, 2H), 6.98 (t, *J* = 8.5 Hz, 2H), 4.56–4.38 (m, 2H), 3.79–3.59 (m, 2H), 3.19 (dt, *J* = 12.8, 6.4 Hz, 1H), 2.69 (bs, 1H), 2.39–2.21 (m, 2H), 1.66 (q, *J* = 12.4 Hz, 1H), 1.56 (s, 3H), 1.32 (s, 3H). ^13^C NMR (100 MHz, CDCl_3_) *δ* ppm: 161.6 (d, *J* = 244.3 Hz), 137.8 (d, *J* = 3.2 Hz), 128.5 (d, *J* = 7.8 Hz), 115.2 (d, *J* = 21.1 Hz), 113.3, 87.1, 83.0, 64.2, 49.9, 47.4, 35.0, 30.9, 27.7, 25.2. ^19^F NMR (376 MHz, CDCl_3_) *δ* ppm: −116.81. HRMS (ESI): calcd for C_15_H_20_FO_3_^+^ [M + H]^+^, 267.1391; found, 267.1383.

#### ((3a*R*,4*R*,6*S*,6a*S*)-6-(4-Methoxyphenyl)-2,2-dimethyltetrahydro-4*H*-cyclopenta[*d*][1,3]dioxol-4-yl)methanol (9c)

Following the general procedure B for alkene 9c (164 mg, 0.59 mmol), the desired compound 10c (143 mg, 87% yield) was isolated as a pale yellow oil. ^1^H NMR (400 MHz, CDCl_3_) *δ* ppm: 7.19 (d, *J* = 8.3 Hz, 2H), 6.85 (d, *J* = 8.3 Hz, 2H), 4.53–4.42 (m, 2H), 3.82–3.69 (m, 5H), 3.23–3.12 (m, 1H), 2.40–2.19 (m, 2H), 1.94 (bs, 1H), 1.72–1.53 (m, 1H), 1.56 (s, 3H), 1.32 (s, 3H). ^13^C NMR (100 MHz, CDCl_3_) *δ* ppm: 158.3, 134.3, 128.0, 113.9, 113.2, 87.3, 83.1, 64.5, 55.3, 49.8, 47.6, 35.1, 27.7, 25.2. HRMS (ESI): calcd for C_16_H_22_NaO_4_^+^ [M + Na]^+^, 301.1410; found, 301.1413.

#### ((3a*R*,4*R*,6*S*,6a*S*)-2,2-Dimethyl-6-(4-(trifluoromethoxy)phenyl)tetrahydro-4*H*-cyclopenta[*d*][1,3]dioxol-4-yl)methanol (9d)

Following the general procedure B for alkene 9d (190 mg, 0.57 mmol), the desired compound 10d (147 mg, 79% yield) was isolated as a dark yellow oil. ^1^H NMR (400 MHz, CDCl_3_) *δ* ppm: 7.31 (d, *J* = 7.7 Hz, 2H), 7.17 (d, *J* = 8.0 Hz, 2H), 4.59–4.44 (m, 2H), 3.86–3.69 (m, 2H), 3.24 (dt, *J* = 12.7, 6.2 Hz, 1H), 2.58–2.22 (m, 3H), 1.78–1.59 (m, 1H), 1.59 (s, 3H), 1.34 (s, 3H). ^13^C NMR (100 MHz, CDCl_3_) *δ* ppm: 147.9 (q, *J* = 10.1 Hz), 140.9, 128.3, 121.0, 120.5 (q, *J* = 252 Hz), 113.5, 87.0, 83.0, 64.2, 50.1, 47.3, 34.8, 27.6, 25.1. ^19^F NMR (376 MHz, CDCl_3_) *δ* ppm: −57.96. HRMS (ESI): calcd for C_16_H_19_F_3_NaO_4_^+^ [M + Na]^+^, 355.1128; found, 355.1129.

#### ((3a*R*,4*R*,6*S*,6a*S*)-2,2-Dimethyl-6-(2-methylpyridin-3-yl)tetrahydro-4*H*-cyclopenta[*d*][1,3]dioxol-4-yl)methanol (10e)

Following the general procedure B for alkene 9e (123 mg, 0.47 mmol), the desired compound 10e (119 mg, 97% yield) was isolated as a dark yellow oil. [*α*]^20^_D_ = −35.0 (*c* = 4.40, CHCl_3_). ^1^H NMR (400 MHz, CDCl_3_) *δ* ppm: 8.28 (bs, 1H), 7.48 (d, *J* = 7.7 Hz, 1H), 7.13–7.01 (m, 1H), 4.55–4.46 (m, 2H), 3.75–3.69 (m, 2H), 3.45–3.36 (m, 1H), 3.12 (bs, 1H), 2.61 (s, 3H), 2.39–3.25 (m, 1H), 2.20–2.15 (m, 1H), 1.71–1.62 (m, 1H), 1.53 (s, 3H), 1.26 (s, 3H). ^13^C NMR (100 MHz, CDCl_3_) *δ* ppm: 157.0, 146.5, 135.8, 133.5, 121.5, 113.2, 87.0, 82.8, 63.5, 47.5, 46.6, 35.0, 27.7, 25.2, 22.7. HRMS (ESI): calcd for C_15_H_22_NO_3_^+^ [M + H]^+^, 264.1594; found, 264.1596.

#### ((3a*R*,4*R*,6*S*,6a*S*)-2,2-Dimethyl-6-(naphthalen-2-yl)tetrahydro-4*H*-cyclopenta[*d*][1,3]dioxol-4-yl)methanol (10f)

Following the general procedure B for alkene 9f (83 mg, 0.28 mmol), the desired compound 10f (66 mg, 80% yield) was isolated as white solid. [*α*]^20^_D_ = −15.3 (*c* = 0.44, CHCl_3_). ^1^H NMR (400 MHz, CDCl_3_) *δ* ppm: 7.85–7.76 (m, 3H), 7.70 (s, 1H), 7.52–7.44 (m, 3H), 4.68–4.51 (m, 2H), 3.88–3.62 (m, 2H), 3.41 (dt, *J* = 12.2, 6.2 Hz, 1H), 2.47–2.34 (m, 2H), 2.04 (bs, 1H), 1.82 (q, *J* = 11.6 Hz, 1H), 1.62 (s, 3H), 1.36 (s, 3H). ^13^C NMR (100 MHz, CDCl_3_) *δ* ppm: 139.7, 133.5, 132.4, 128.2, 127.7, 127.6, 126.0, 125.8, 125.4, 125.2, 113.4, 87.0, 83.2, 64.5, 50.7, 47.6, 34.9, 27.7, 25.2. HRMS (ESI): calcd for C_19_H_22_NaO_3_^+^ [M + Na]^+^, 321.1461; found, 321.1464.

#### ((3a*R*,4*R*,6*S*,6a*S*)-6-(3-Methoxynaphthalen-2-yl)-2,2-dimethyltetrahydro-4*H*-cyclopenta[*d*][1,3]dioxol-4-yl)methanol (10g)

Following the general procedure B for alkene 9g (249 mg, 0.76 mmol), the desired compound 10g (249 mg, quant) was isolated as a white foam. ^1^H NMR (400 MHz, CDCl_3_) *δ* ppm: 7.73 (t, *J* = 7.1 Hz, 2H), 7.67 (s, 1H), 7.42 (t, *J* = 7.5 Hz, 1H), 7.34 (t, *J* = 7.5 Hz, 1H), 7.14 (s, 1H), 4.96–4.86 (m, 1H), 4.68–4.52 (m, 1H), 3.96 (s, 3H), 3.84–3.71 (m, 2H), 3.62 (dt, *J* = 19.6, 7.0 Hz, 1H), 2.48–2.26 (m, 2H), 1.82 (dd, *J* = 24.1, 11.9 Hz, 1H), 1.61 (s, 3H), 1.36 (s, 3H). ^13^C NMR (100 MHz, CDCl_3_) *δ* ppm: 156.2, 133.5, 132.0, 128.8, 127.3, 127.3, 126.2, 125.9, 123.7, 112.8, 105.6, 85.5, 83.7, 64.9, 55.3, 48.3, 47.0, 35.1, 27.9, 25.4. HRMS (ESI): calcd for C_20_H_24_NaO_4_^+^ [M + Na]^+^, 351.1567; found, 351.1569.

#### ((3a*R*,4*R*,6*S*,6a*S*)-2,2-Dimethyl-6-(naphthalen-1-yl)tetrahydro-4*H*-cyclopenta[*d*][1,3]dioxol-4-yl)methanol (10h)

Following the general procedure B for alkene 9h (206 mg, 0.7 mmol), the desired compound 10h (160 mg, 77% yield) was isolated as a colourless viscous oil. [*α*]^20^_D_ = −60.2 (*c* = 5.60, CH_3_OH). ^1^H NMR (400 MHz, CDCl_3_) *δ* ppm: 8.32 (d, *J* = 8.4 Hz, 1H), 7.89 (d, *J* = 7.9 Hz, 1H), 7.77 (d, *J* = 6.9 Hz, 1H), 7.60–7.43 (m, 4H), 4.77 (t, *J* = 6.4 Hz, 1H), 4.66–4.56 (m, 1H), 4.08 (dt, *J* = 12.1, 6.2 Hz, 1H), 3.82–3.64 (m, 2H), 2.51 (dt, *J* = 17.7, 6.0 Hz, 1H), 2.47–2.34 (m, 1H), 2.13 (s, 1H), 1.98–1.85 (m, 1H), 1.68 (s, 3H), 1.36 (s, 3H). ^13^C NMR (100 MHz, CDCl_3_) *δ* ppm: 138.3, 134.0, 132.3, 128.8, 127.2, 126.0, 125.6, 125.4, 124.1, 122.7, 113.0, 86.8, 83.2, 64.4, 48.0, 46.2, 35.1, 27.8, 25.2. HRMS (ESI): calcd for C_19_H_22_NaO_3_^+^ [M + Na]^+^, 321.1461; found, 321.1463.

#### ((3a*R*,4*R*,6*S*,6a*S*)-6-(2,4-Difluoro-5-methylphenyl)-2,2-dimethyltetrahydro-4*H*-cyclopenta[*d*][1,3]dioxol-4-yl)methanol (10i)

Following the general procedure B for alkene 9i (85 mg, 0.29 mmol), the desired compound 10i (85 mg, quant) was isolated as black solid. ^1^H NMR (400 MHz, CDCl_3_) *δ* ppm: 7.04 (t, *J* = 8.4 Hz, 1H), 6.73 (t, *J* = 10.0 Hz, 1H), 4.65 (t, *J* = 6.8 Hz, 1H), 4.57–4.43 (m, 1H), 3.74 (ddd, *J* = 17.3, 10.6, 6.3 Hz, 2H), 3.31 (dt, *J* = 12.9, 6.6 Hz, 1H), 2.36 (dt, *J* = 12.1, 6.0 Hz, 1H), 2.28–2.16 (m, 4H), 1.83 (s, 1H), 1.71 (q, *J* = 12.5 Hz, 1H), 1.56 (s, 3H), 1.32 (s, 3H). ^13^C NMR (100 MHz, CDCl_3_) *δ* ppm: 160.8 (dd, *J* = 56.6, 11.7 Hz), 158.3 (dd, *J* = 56.0, 11.7 Hz), 130.9 (t, *J* = 6.4 Hz), 124.2 (dd, *J* = 14.1, 4.1 Hz), 120.5 (dd, *J* = 17.1, 3.9 Hz), 113.4, 103.7 (t, *J* = 26.3 Hz), 85.9, 83.1, 64.4, 47.9, 45.5, 35.0 (d, *J* = 2.1 Hz), 27.8, 25.4, 14.1 (d, *J* = 3.0 Hz). ^19^F NMR (376 MHz, CDCl_3_) *δ* ppm: −115.25 to −116.78 (m), −117.45 to −118.18 (m). HRMS (ESI): calcd for C_16_H_20_F_2_NaO_3_^+^ [M + Na]^+^, 321.1273; found, 321.1273.

#### ((3a*R*,4*R*,6*S*,6a*S*)-2,2-Dimethyl-6-(pyridin-3-yl)tetrahydro-4*H*-cyclopenta[*d*][1,3]dioxol-4-yl)methanol (10j)

Following the general procedure B for alkene 9j (116 mg, 0.47 mmol), the desired compound 10j (95 mg, 81% yield) was isolated as a colourless oil. ^1^H NMR (400 MHz, CDCl_3_) *δ* ppm: 8.61–8.39 (m, 2H), 7.62 (d, *J* = 7.8 Hz, 1H), 7.29–7.22 (m, 1H), 4.61–4.45 (m, 2H), 3.86–3.71 (m, 2H), 3.23 (dt, *J* = 12.8, 6.5 Hz, 1H), 2.52–2.23 (m, 3H), 1.81–1.68 (m, 1H), 1.58 (s, 3H), 1.33 (s, 3H). ^13^C NMR (100 MHz, CDCl_3_) *δ* ppm: 148.6, 147.9, 137.6, 134.7, 123.4, 113.5, 86.7, 82.9, 63.9, 48.5, 47.4, 34.5, 27.7, 25.2. HRMS (ESI): calcd for C_14_H_20_NO_3_^+^ [M + H]^+^, 250.1438; found, 250.1439.

#### ((3a*R*,4*R*,6*S*,6a*S*)-6-(6-Fluoropyridin-3-yl)-2,2-dimethyltetrahydro-4*H*-cyclopenta[*d*][1,3]dioxol-4-yl)methanol (10k)

Following the general procedure B for alkene 9k (75 mg, 0.28 mmol), the desired compound 10k (69 mg, 93% yield) was isolated as a colourless viscous oil. ^1^H NMR (400 MHz, CDCl_3_) *δ* ppm: 8.10 (s, 1H), 7.74 (td, *J* = 8.2, 1.9 Hz, 1H), 6.90 (dd, *J* = 8.3, 2.0 Hz, 1H), 4.59–4.51 (m, 1H), 4.46 (t, *J* = 7.1 Hz, 1H), 3.77 (dt, *J* = 16.6, 10.5 Hz, 2H), 3.22 (dt, *J* = 12.9, 6.5 Hz, 1H), 2.60 (s, 1H), 2.44–2.28 (m, 2H), 1.79–1.66 (m, 1H), 1.57 (s, 3H), 1.33 (s, 3H). ^13^C NMR (100 MHz, CDCl_3_) *δ* ppm: 162.7 (d, *J* = 238.3 Hz), 145.8 (d, *J* = 14.1 Hz), 140.1 (d, *J* = 7.7 Hz), 135.4 (d, *J* = 4.5 Hz), 113.7, 109.4 (d, *J* = 37.1 Hz), 86.8, 82.9, 63.8, 47.8, 47.2, 34.5, 27.7, 25.2. ^19^F NMR (376 MHz, CDCl_3_) *δ* ppm: −71.47. Calcd for C_14_H_19_FNO_3_^+^ [M + H]^+^, 268.1271; found, 268.1340.

#### 4-((3a*S*,4*S*,6*R*,6a*R*)-6-(Hydroxymethyl)-2,2-dimethyltetrahydro-4*H*-cyclopenta[*d*][1,3]dioxol-4-yl)benzonitrile (10l)

Following the general procedure B for alkene 9l (274 mg, 1.02 mmol), the desired compound 10l (112 mg, 40% yield) was isolated as a yellow oil. ^1^H NMR (400 MHz, CDCl_3_) *δ* ppm: 7.60 (d, *J* = 8.1 Hz, 2H), 7.40 (d, *J* = 8.1 Hz, 2H), 4.67–4.40 (m, 2H), 3.89–3.64 (m, 2H), 3.27 (dt, *J* = 13.0, 6.6 Hz, 1H), 2.44–2.22 (m, 3H), 1.81–1.65 (m, 1H), 1.58 (s, 3H), 1.33 (s, 3H). ^13^C NMR (100 MHz, CDCl_3_) *δ* ppm: 147.9, 132.4, 128.0, 119.0, 113.7, 110.4, 86.7, 82.9, 64.0, 50.9, 47.2, 34.4, 27.7, 25.2. HRMS (ESI): calcd for C_16_H_20_NO_3_^+^ [M + H]^+^, 274.1438; found, 274.1439.

#### (1*S*,2*R*,3*R*,5*S*)-3-(Hydroxymethyl)-5-phenylcyclopentane-1,2-diol (11a)

Following the general procedure C for isopropylidene 10a (61 mg, 0.24 mmol), the desired compound 11a (50 mg, quantitative yield) was isolated as pale yellow solid.^20^^1^H NMR (400 MHz, DMSO) *δ* ppm: 7.31–7.23 (m, 4H), 7.17 (dd, *J* = 6.6, 5.4 Hz, 1H), 4.55 (t, *J* = 5.1 Hz, 1H), 4.48–4.33 (m, 2H), 3.78–3.64 (m, 2H), 3.37–3.48 (m, 2H), 2.96 (dd, *J* = 11.2, 7.7 Hz, 1H), 2.12–1.94 (m, 2H), 1.30–1.23 (m, 1H). ^13^C NMR (100 MHz, DMSO) *δ* ppm: 144.5, 128.5, 127.9, 126.2, 78.7, 74.0, 63.6, 49.6, 47.3, 32.3. HRMS (ESI): calcd for C_12_H_17_O_3_^+^ [M + H]^+^, 209.1172; found, 209.1176.

#### (1*R*,2*S*,3*S*,5*R*)-3-(4-Fluorophenyl)-5-(hydroxymethyl)cyclopentane-1,2-diol (11b)

Following the general procedure C for isopropylidene 10b (100 mg, 0.37 mmol), the desired compound 11b (54 mg, 64% yield) was isolated as white solid. [*α*]^20^_D_ = −31.3 (*c* = 0.10, CH_3_OH). ^1^H NMR (400 MHz, DMSO) *δ* ppm: 7.34–7.27 (m, 2H), 7.10 (t, *J* = 8.4 Hz, 2H), 4.57 (t, *J* = 5.1 Hz, 1H), 4.46 (d, *J* = 6.7 Hz, 1H), 4.40 (d, *J* = 3.8 Hz, 1H), 3.76–3.62 (m, 2H), 3.48–3.37 (m, 2H), 2.99 (dd, *J* = 18.6, 8.8 Hz, 1H), 2.06–1.91 (m, 2H), 1.32–1.25 (m, 1H). ^13^C NMR (100 MHz, DMSO) *δ* ppm: 161.1 (d, *J* = 241.0 Hz), 140.5 (d, *J* = 3.0 Hz), 129.6 (d, *J* = 7.8 Hz), 115.1 (d, *J* = 20.8 Hz), 78.7 (s), 73.9 (s), 63.6 (s), 48.7 (s), 47.21 (s), 32.2 (s). ^19^F NMR (376 MHz, DMSO) *δ* ppm: −117.72. HRMS (ESI): calcd for C_12_H_16_FO_3_^+^ [M + H]^+^, 227.1079; found, 227.1080.

#### (1*S*,2*R*,3*R*,5*S*)-3-(Hydroxymethyl)-5-(4-methoxyphenyl)cyclopentane-1,2-diol (11c)

Following the general procedure C for isopropylidene 10c (80 mg, 0.29 mmol), the desired compound 11c (69 mg, quantitative yield) was isolated as white solid. [*α*]^20^_D_ = −44.0 (*c* = 1.00, CH_3_OH). ^1^H NMR (400 MHz, DMSO) *δ* ppm: 7.18 (d, *J* = 7.6 Hz, 2H), 6.85 (d, *J* = 7.6 Hz, 2H), 4.54 (t, *J* = 4.8 Hz, 1H), 4.40–4.31 (m, 2H), 3.75–3.61 (m, 5H), 3.48–3.35 (m, 2H), 2.97–2.85 (m, 1H), 2.03–1.96 (m, 2H), 1.27–1.18 (m, 1H). ^13^C NMR (100 MHz, DMSO) *δ* ppm: 158.0, 136.4, 128.8, 114.0, 78.7, 73.9, 63.6, 55.5, 48.7, 47.2, 32.3. HRMS (ESI): calcd for C_13_H_19_O_4_^+^ [M + H]^+^, 239.1278; found, 239.1278.

#### (1*S*,2*R*,3*R*,5*S*)-3-(Hydroxymethyl)-5-(4-(trifluoromethoxy)phenyl)cyclopentane-1,2-diol (11d)

Following the general procedure C for isopropylidene 10d (91 mg, 0.28 mmol), the desired compound 11d (78 mg, 96% yield) was isolated as white solid. [*α*]^20^_D_ = −33.8 (*c* = 1.00, CH_3_OH). ^1^H NMR (400 MHz, DMSO) *δ* ppm: 7.40 (d, *J* = 8.6 Hz, 2H), 7.27 (d, *J* = 8.2 Hz, 2H), 4.72–4.38 (m, 3H), 3.79–3.62 (m, 2H), 3.47–3.36 (m, 2H), 3.04 (dt, *J* = 11.4, 8.2 Hz, 1H), 2.14–1.94 (m, 2H), 1.35–1.26 (m, 1H). ^13^C NMR (100 MHz, DMSO) *δ* ppm: 147.01 (q, *J* = 1.9 Hz), 143.9, 129.6, 121.2, 120.6 (q, *J* = 254.0 Hz), 78.7, 73.9, 63.6, 48.7, 47.2, 32.0. ^19^F NMR (376 MHz, DMSO) *δ* ppm: −56.83. HRMS (ESI): calcd for C_13_H_16_F_3_O_4_^+^ [M + H]^+^, 293.0995; found, 293.0998.

#### (1*S*,2*R*,3*R*,5*S*)-3-(Hydroxymethyl)-5-(2-methylpyridin-3-yl)cyclopentane-1,2-diol (11e)

Following the general procedure C for isopropylidene 10e (69 mg, 0.26 mmol), the desired compound 11e (21 mg, 36% yield) was isolated as pale yellow highly viscous oil. ^1^H NMR (400 MHz, DMSO) *δ* ppm: 8.24 (d, *J* = 4.5 Hz, 1H), 7.64 (d, *J* = 7.8 Hz, 1H), 7.21–7.15 (m, 1H), 4.60 (t, *J* = 4.7 Hz, 1H), 4.48 (dd, *J* = 23.4, 4.7 Hz, 2H), 3.84 (dd, *J* = 13.2, 6.2 Hz, 1H), 3.76 (s, 1H), 3.43 (dd, *J* = 11.4, 5.7 Hz, 2H), 3.26 (dd, *J* = 18.3, 8.9 Hz, 1H), 2.51 (s, 5H), 2.08 (dt, *J* = 18.3, 9.5 Hz, 2H), 1.15 (dd, *J* = 18.9, 11.3 Hz, 1H). ^13^C NMR (100 MHz, DMSO) *δ* ppm: 157.2, 146.3, 137.6, 133.7, 121.9, 78.2, 73.8, 63.5, 47.1, 44.6, 31.6, 23.0. HRMS (ESI): calcd for C_12_H_18_NO_3_^+^ [M + H]^+^, 224.1281; found, 224.1282.

#### (1*S*,2*R*,3*R*,5*S*)-3-(Hydroxymethyl)-5-(naphthalen-2-yl)cyclopentane-1,2-diol (11f)

Following the general procedure C for isopropylidene 10f (39 mg, 0.13 mmol), the desired compound 11f (24 mg, 73% yield) was isolated as white solid. [*α*]^20^_D_ = −28.4 (*c* = 1.00, CH_3_OH). ^1^H NMR (400 MHz, DMSO) *δ* ppm: 7.91–7.80 (m, 3H), 7.76 (s, 1H), 7.53–7.40 (m, 3H), 4.68–4.39 (m, 3H), 3.94–3.75 (m, 2H), 3.56–3.42 (m, 2H), 3.24–3.08 (m, 1H), 2.21–1.98 (m, 2H), 1.51–1.38 (m, 1H). ^13^C NMR (100 MHz, DMSO) *δ* ppm: 142.1, 133.6, 132.2, 128.0, 127.8, 126.9, 126.3, 125.9, 125.6, 78.6, 74.1, 63.6, 49.7, 47.3, 32.2. HRMS (ESI): calcd for C_16_H_19_O_3_^+^ [M + H]^+^, 259.1329; found, 259.1331.

#### (1*S*,2*R*,3*R*,5*S*)-3-(Hydroxymethyl)-5-(3-methoxynaphthalen-2-yl)cyclopentane-1,2-diol (11g)

Following the general procedure C for isopropylidene 10g (250 mg, 0.76 mmol), the desired compound 11g (124 mg, 57% yield) was isolated as white foam after purification by flash chromatography using CH_2_Cl_2_ : MeOH (50 : 5) to (45 : 5). [*α*]^20^_D_ = −70.4 (*c* = 1.00, CH_3_OH). ^1^H NMR (400 MHz, MeOD) *δ* ppm: 7.80–7.63 (m, 3H), 7.36 (t, *J* = 7.5 Hz, 1H), 7.28 (t, *J* = 7.5 Hz, 1H), 7.18 (s, 1H), 4.29 (dd, *J* = 7.5, 5.7 Hz, 1H), 4.02 (t, *J* = 5.0 Hz, 1H), 3.90 (s, 3H), 3.75–3.51 (m, 3H), 2.36–2.16 (m, 2H), 1.44 (dt, *J* = 10.4, 8.0 Hz, 1H). ^13^C NMR (100 MHz, MeOD) *δ* ppm: 156.6, 133.5, 132.8, 129.0, 126.9, 126.3, 126.0, 125.3, 123.2, 104.9, 76.3, 74.2, 63.7, 54.4, 46.7, 44.8, 31.0. HRMS (ESI): calcd for C_17_H_21_O_4_^+^ [M + H]^+^, 289.1434; found, 289.1432.

#### (1*S*,2*R*,3*R*,5*S*)-3-(Hydroxymethyl)-5-(naphthalen-1-yl)cyclopentane-1,2-diol (11h)

Following the general procedure C for isopropylidene 10h (91 mg, 0.28 mmol), the desired compound 11h (78 mg, 96% yield) was isolated as white solid. ^1^H NMR (400 MHz, MeOD) *δ* ppm: 8.26 (d, *J* = 8.4 Hz, 1H), 7.84 (d, *J* = 7.9 Hz, 1H), 7.71 (d, *J* = 8.0 Hz, 1H), 7.53–7.42 (m, 4H), 4.26 (dd, *J* = 7.6, 5.6 Hz, 1H), 4.09–3.96 (m, 2H), 3.74–3.58 (m, 2H), 2.44 (dt, *J* = 12.9, 8.2 Hz, 1H), 2.37–2.26 (m, 1H), 1.48–1.36 (m, 1H). ^13^C NMR (100 MHz, MeOD) *δ* ppm: 139.2, 134.1, 132.7, 128.3, 126.3, 125.4, 125.3, 125.0, 123.5, 122.1, 77.2, 74.2, 63.5, 46.5, 44.1, 32.1. HRMS (ESI): calcd for C_16_H_19_O_3_^+^ [M + H]^+^, 259.1329; found, 259.1329.

#### (1*R*,2*S*,3*S*,5*R*)-3-(2,4-Difluoro-5-methylphenyl)-5-(hydroxymethyl)cyclopentane-1,2-diol (11i)

Following the general procedure C for isopropylidene 10i (78 mg, 0.26 mmol), the desired compound 11i (43 mg, 64% yield) was isolated as white solid. ^1^H NMR (400 MHz, MeOD) *δ* ppm: 7.10 (t, *J* = 8.5 Hz, 1H), 6.69 (t, *J* = 10.1 Hz, 1H), 3.98–3.73 (m, 2H), 3.57–3.40 (m, 2H), 3.24–3.18 (m, 1H), 2.12–2.04 (m, 4H), 1.31–1.22 (m, 1H). ^13^C NMR (100 MHz, MeOD) *δ* ppm: 161.5–159.1 (m), 158.2 (dd, *J* = 13.3, 11.9 Hz), 130.5 (t, *J* = 6.3 Hz), 125.3 (dd, *J* = 14.4, 4.1 Hz), 120.0 (dd, *J* = 17.0, 3.9 Hz), 102.5 (t, *J* = 26.8 Hz), 76.9, 73.7, 63.4, 46.8, 42.6, 30.9, 12.5 (d, *J* = 3.2 Hz). ^19^F NMR (376 MHz, MeOD) *δ* ppm: −118.98 to −119.60 (m), −120.00 (dd, *J* = 16.3, 8.6 Hz). HRMS (ESI): calcd for C_13_H_17_F_2_O_3_^+^ [M + H]^+^, 259.1140; found, 259.1138.

#### (1*S*,2*R*,3*R*,5*S*)-3-(Hydroxymethyl)-5-(pyridin-3-yl)cyclopentane-1,2-diol (11j)

Following the general procedure C for isopropylidene 10j (79 mg, 0.31 mmol), the desired compound 11j (9 mg, 13% yield) was isolated as white solid. ^1^H NMR (400 MHz, MeOD) *δ* ppm: 8.51 (s, 1H), 8.40 (d, *J* = 4.7 Hz, 1H), 7.84 (d, *J* = 8.0 Hz, 1H), 7.41 (dd, *J* = 7.8, 4.9 Hz, 1H), 4.04–3.86 (m, 2H), 3.66 (d, *J* = 5.3 Hz, 2H), 3.20 (dt, *J* = 16.3, 7.0 Hz, 1H), 2.31–2.13 (m, 2H), 1.54–1.44 (m, 1H). ^13^C NMR (100 MHz, MeOD) *δ* ppm: 148.3, 146.5, 139.5, 135.7, 123.8, 78.4, 73.9, 63.3, 46.9, 46.7, 30.9. HRMS (ESI): calcd for C_11_H_16_NO_3_^+^ [M + H]^+^, 210.1125; found, 210.1125.

#### (1*R*,2*S*,3*S*,5*R*)-3-(6-Fluoropyridin-3-yl)-5-(hydroxymethyl)cyclopentane-1,2-diol (11k)

Following the general procedure C for isopropylidene 10k (62 mg, 0.23 mmol), the desired compound 11k (26 mg, 50% yield) was isolated as colourless oil. ^1^H NMR (400 MHz, MeOD) *δ* ppm: 8.13 (s, 1H), 7.93 (td, *J* = 8.2, 2.3 Hz, 1H), 7.02 (dd, *J* = 8.5, 2.1 Hz, 1H), 4.01–3.81 (m, 2H), 3.63 (d, *J* = 5.4 Hz, 2H), 3.22–3.11 (m, 1H), 2.28–2.12 (m, 2H), 1.50–1.37 (m, 1H). ^13^C NMR (100 MHz, MeOD) *δ* ppm: 162.5 (d, *J* = 237.4 Hz), 145.9 (d, *J* = 13.4 Hz), 140.7 (d, *J* = 7.9 Hz), 136.8 (d, *J* = 4.4 Hz), 108.9 (d, *J* = 36.7 Hz), 78.4, 73.8, 63.4, 46.9, 45.9, 30.9. ^19^F NMR (376 MHz, MeOD) *δ* ppm: −75.20. HRMS (ESI): calcd for C_11_H_15_FNO_3_^+^ [M + H]^+^, 228.0958; found, 228.1030.

#### 4-((1*S*,2*S*,3*R*,4*R*)-2,3-Dihydroxy-4-(hydroxymethyl)cyclopentyl)benzonitrile (11l)

Following the general procedure C for isopropylidene 10l (10 mg, 0.037 mmol), the desired compound 11l (8 mg, quant yield) was isolated as colourless oi. ^1^H NMR (400 MHz, MeOD) *δ* ppm: 7.68 (d, *J* = 8.2 Hz, 2H), 7.52 (d, *J* = 8.2 Hz, 2H), 3.99–3.87 (m, 2H), 3.70–3.60 (m, 2H), 3.28–3.16 (m, 1H), 2.27–2.12 (m, 2H), 1.52–1.43 (m, 1H). ^13^C NMR (100 MHz, MeOD) *δ* ppm: 149.4, 131.8, 128.4, 118.5, 109.5, 78.4, 74.0, 63.3, 49.5, 46.9, 31.2. HRMS (ESI): calcd for C_13_H_16_NO_3_^+^ [M + H]^+^, 234.1125; found, 234.1128.

## Conflicts of interest

There are no conflicts to declare.

## Supplementary Material

RA-013-D3RA04937J-s001
