# Supplementary material for: Cobalt-assisted route to rare carbocyclic C-ribonucleosides
Source: RSC Adv. 2023 Oct 20;13(44):30777–86. doi: 10.1039/d3ra04937j (PMC10587889; doi:10.1039/d3ra04937j)
Supplement: RA-013-D3RA04937J-s001 [file RA-013-D3RA04937J-s001.pdf]

Electronic Supplementary Material (ESI) for RSC Advances.
This journal is © The Royal Society of Chemistry 2023

Cobalt-assisted Route to Rare Carbocyclic C-Ribonucleosides

A. C. Ojeda-Porras,[a] V. Roy,*[,a] O. Bourzikat,[a] P. Favetta[a] and L. A. Agrofoglio*[,a]

a. Université d'Orléans et CNRS, ICOA, UMR 7311, F-45067, Orléans, France.
E-mail : luigi.agrofoglio@univ-orleans.fr

Contents

I. NMR Spectra of all synthesized compounds..2

II. 3D view of best conformer of compounds **2** and **3** using Cheminfo.org ………...46

(3aR,6aR)-2,2-dimethyl-3a,6a-dihydro-4H-cyclopenta[d][1,3]dioxol-4-one (1)

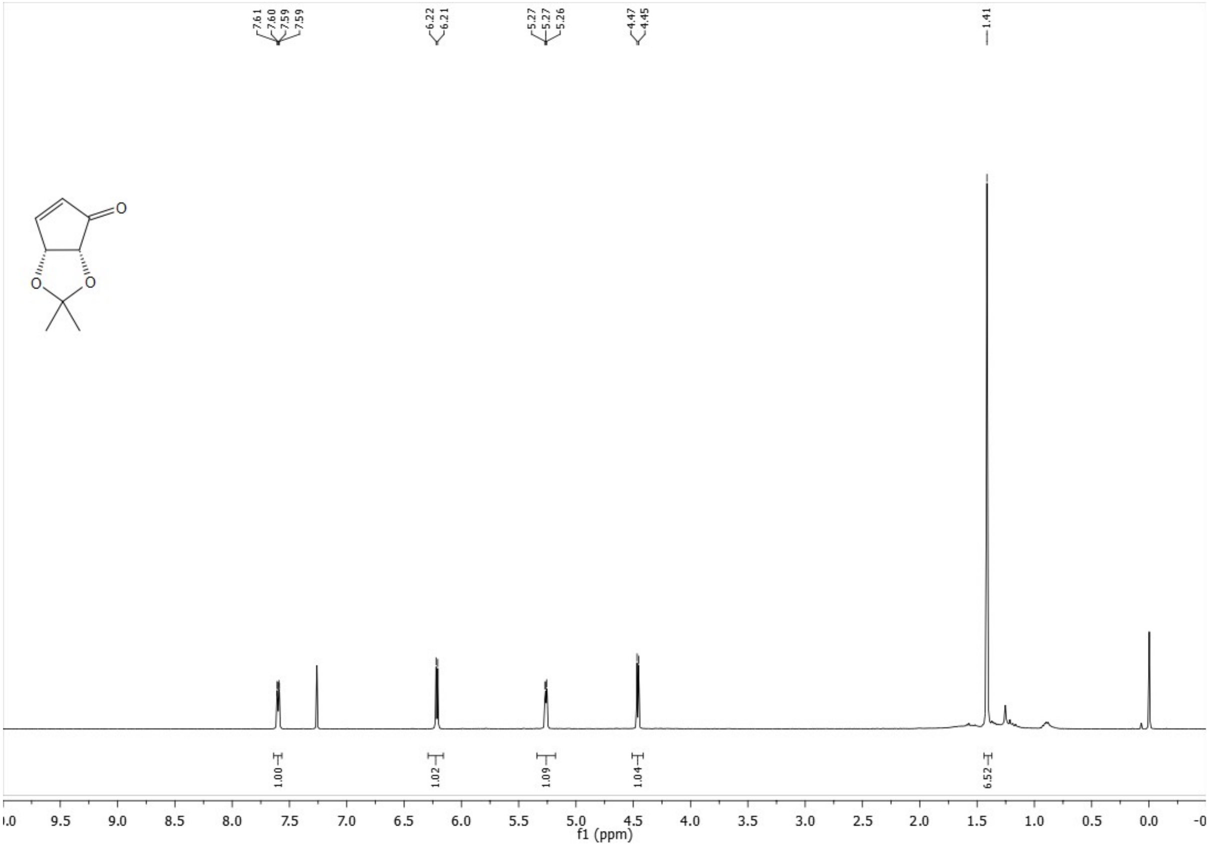

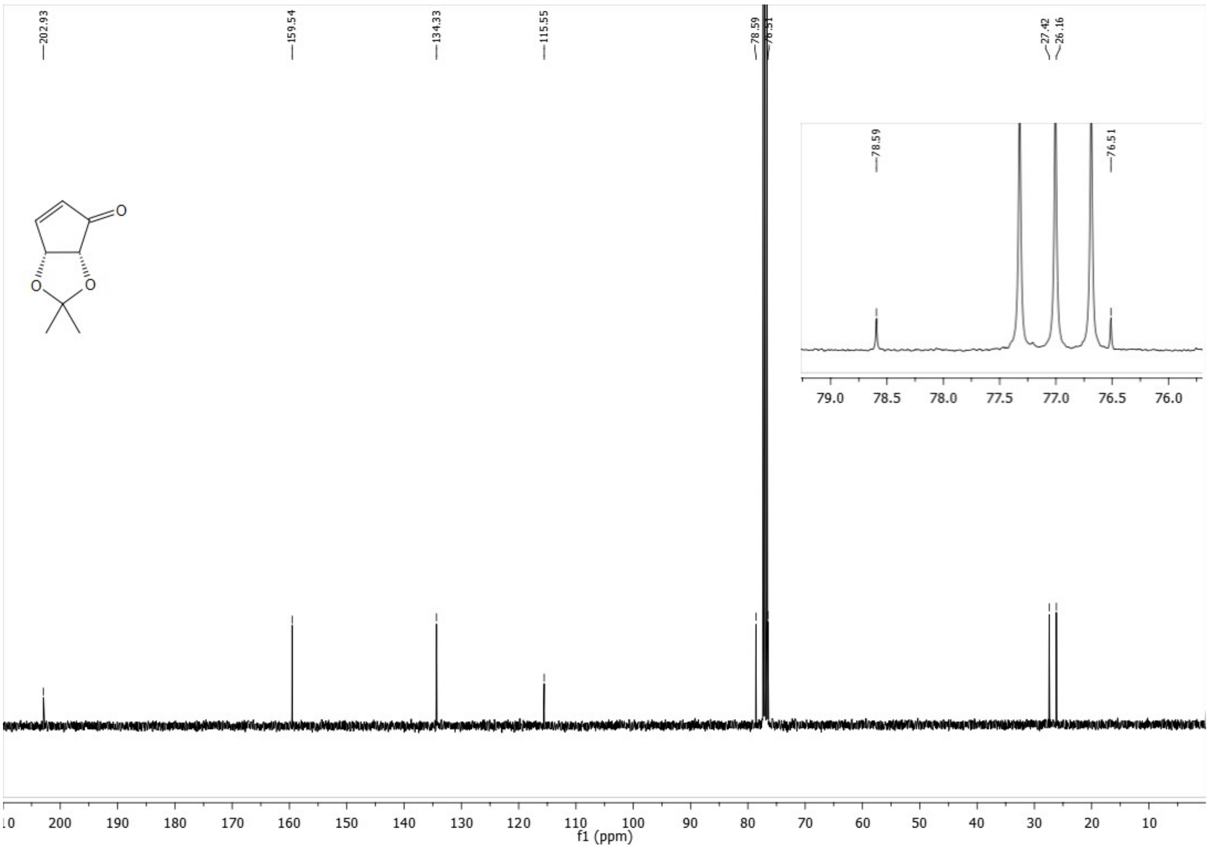

(3aR,6R,6aR)-2,2-dimethyl-6-vinyltetrahydro-4H-cyclopenta[d][1,3]dioxol-4-one (2)

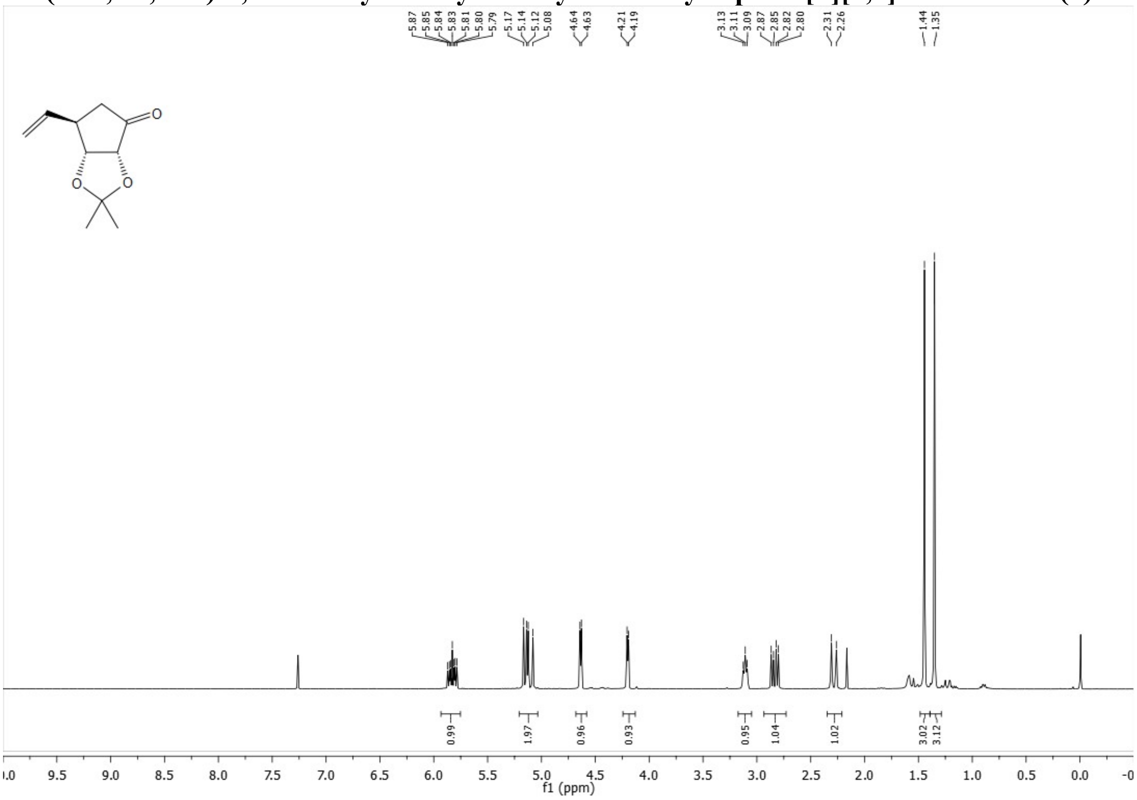

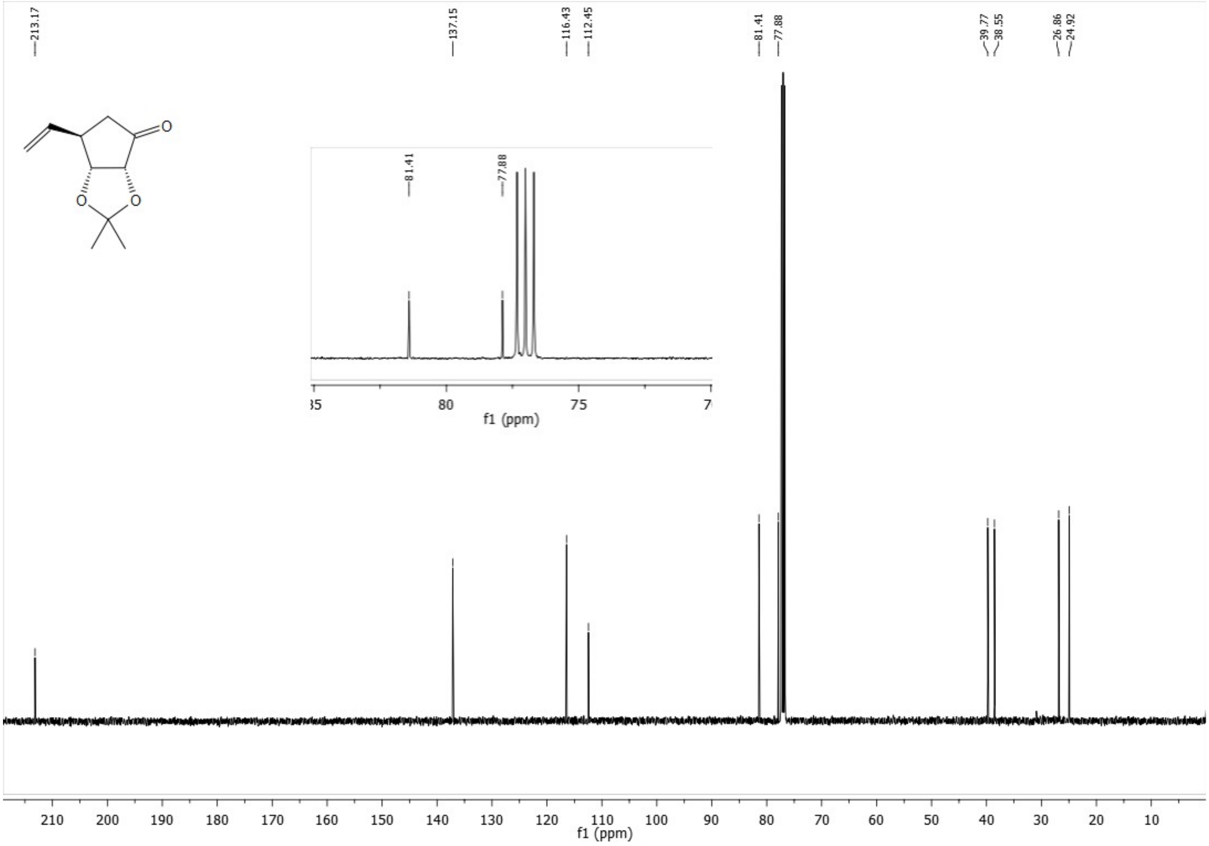

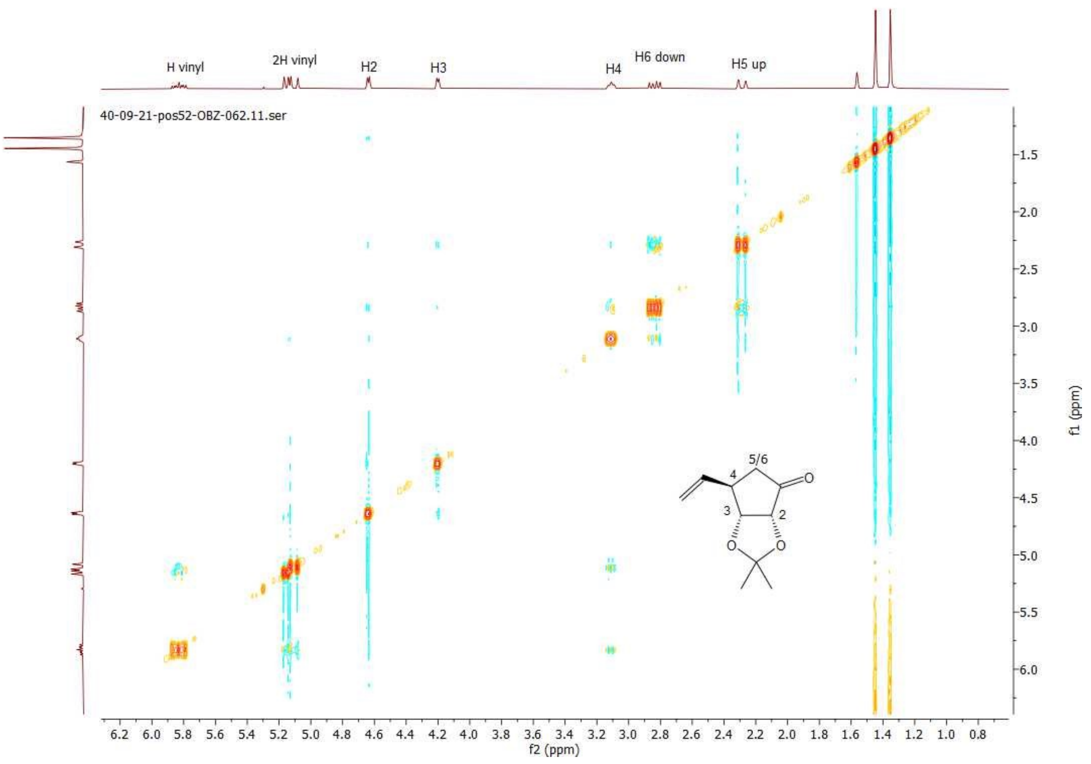

(3aS,4S,6R,6aR)-2,2-dimethyl-6-vinyltetrahydro-4H-cyclopenta[d][1,3]dioxol-4-ol (3)

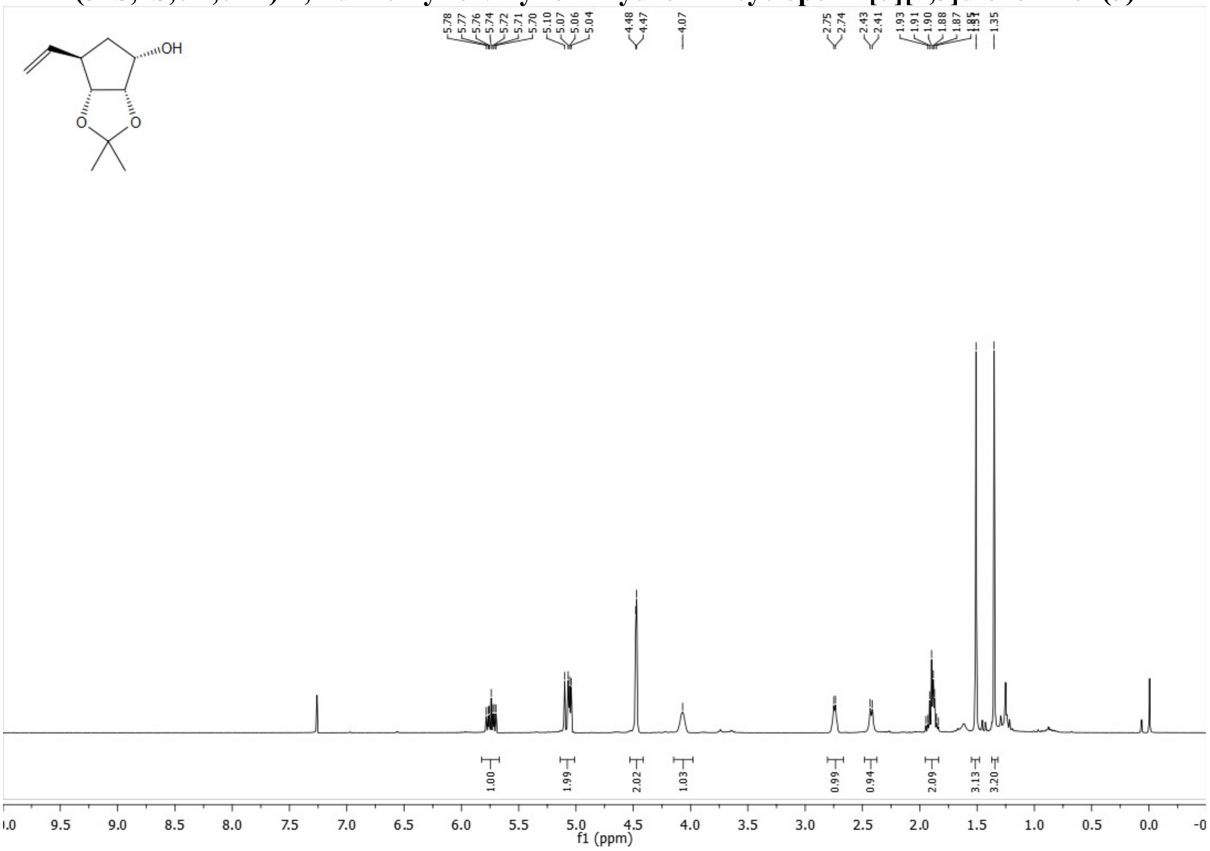

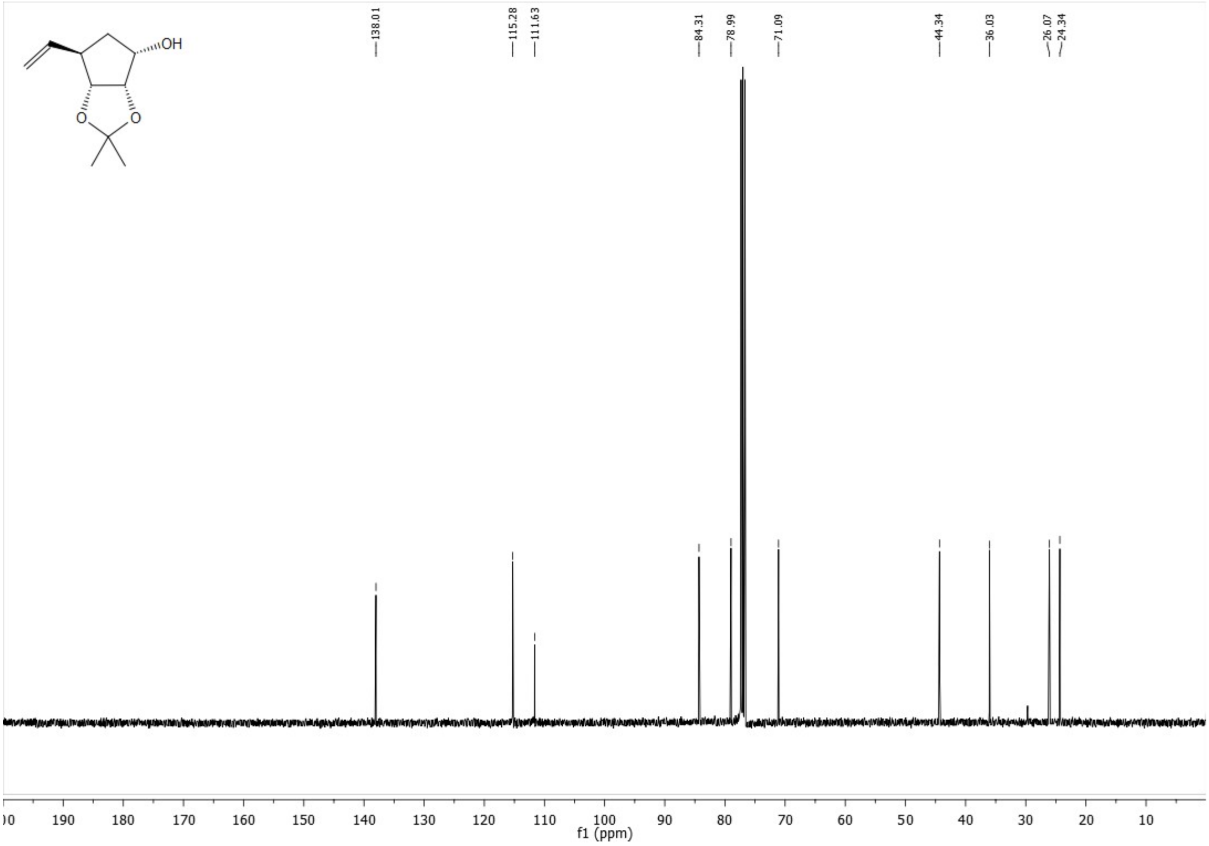

(3aS,4S,6R,6aR)-2,2-dimethyl-6-vinyltetrahydro-4H-cyclopenta[d][1,3]dioxol-4-ol (3)
[$CDCl_3$+D_2O]

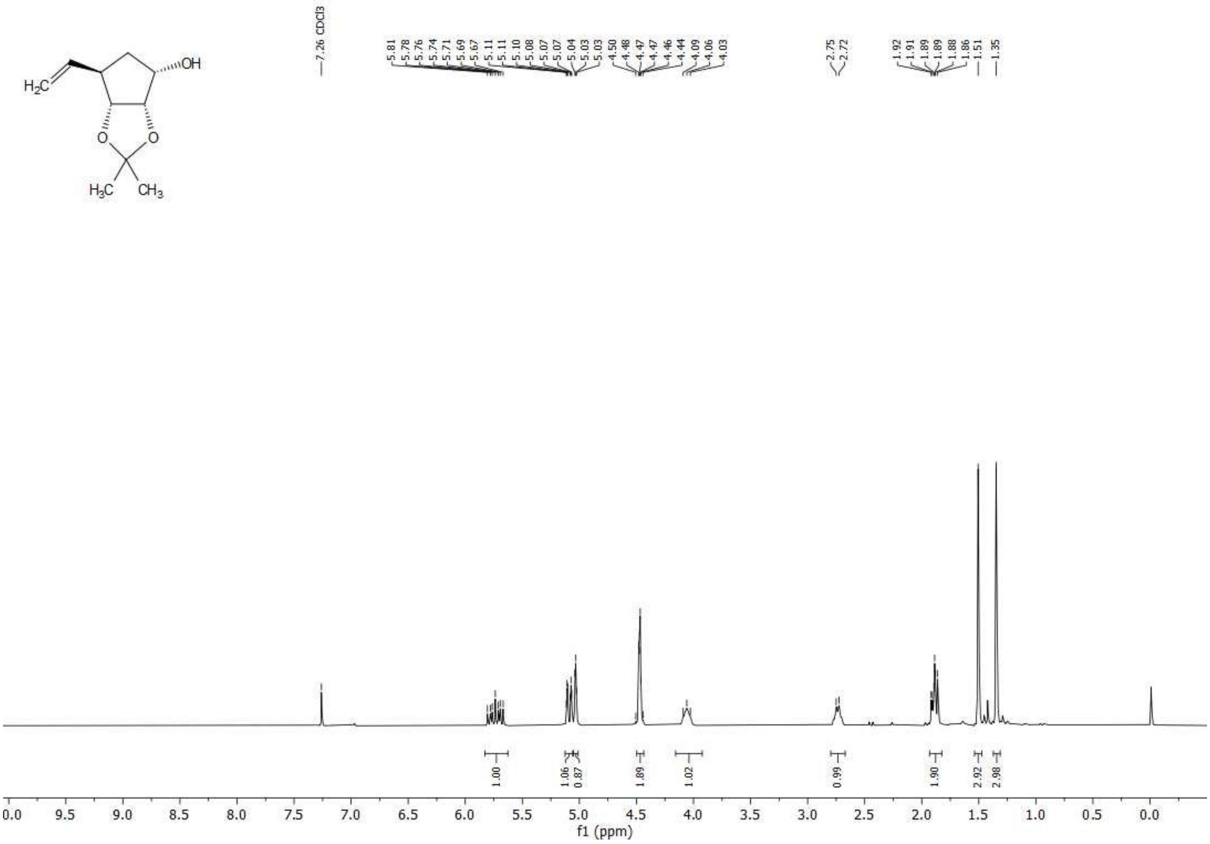

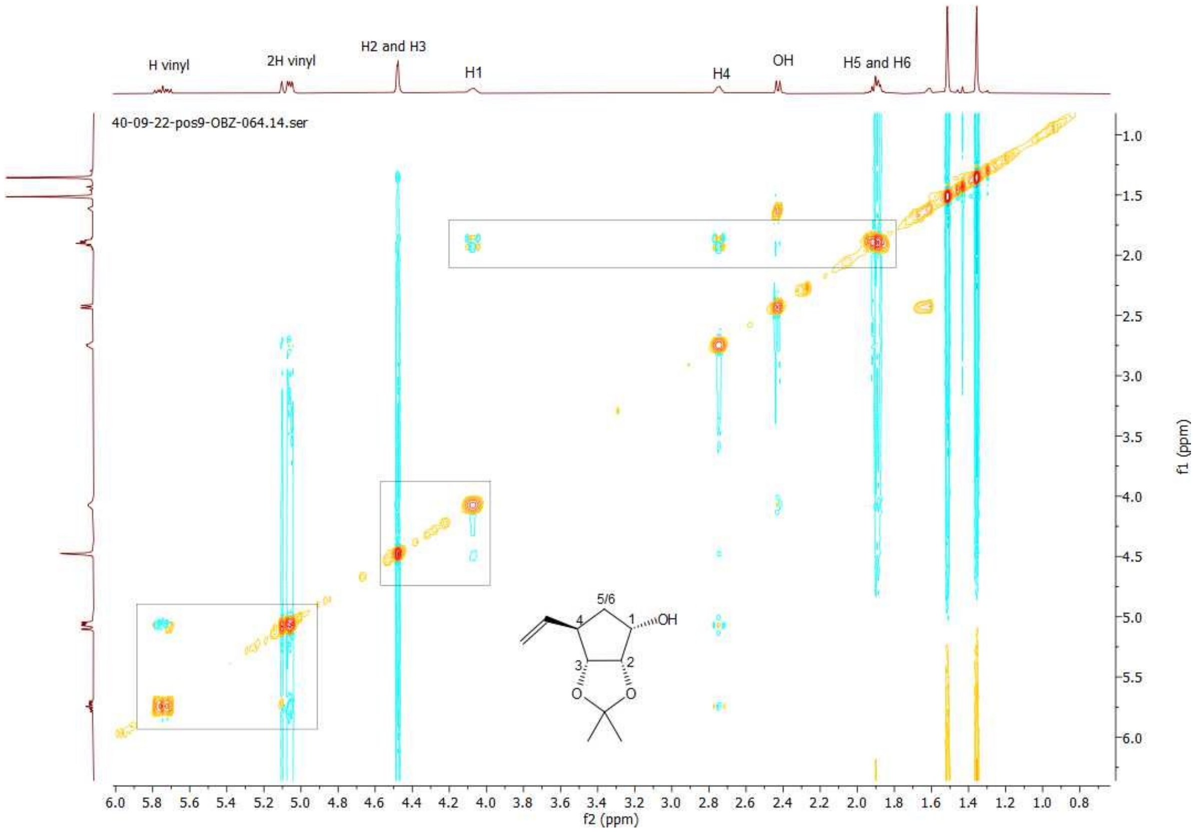

(3aR,4R,6R,6aR)-4-iodo-2,2-dimethyl-6-vinyltetrahydro-4H-cyclopenta[d][1,3]dioxole (4)

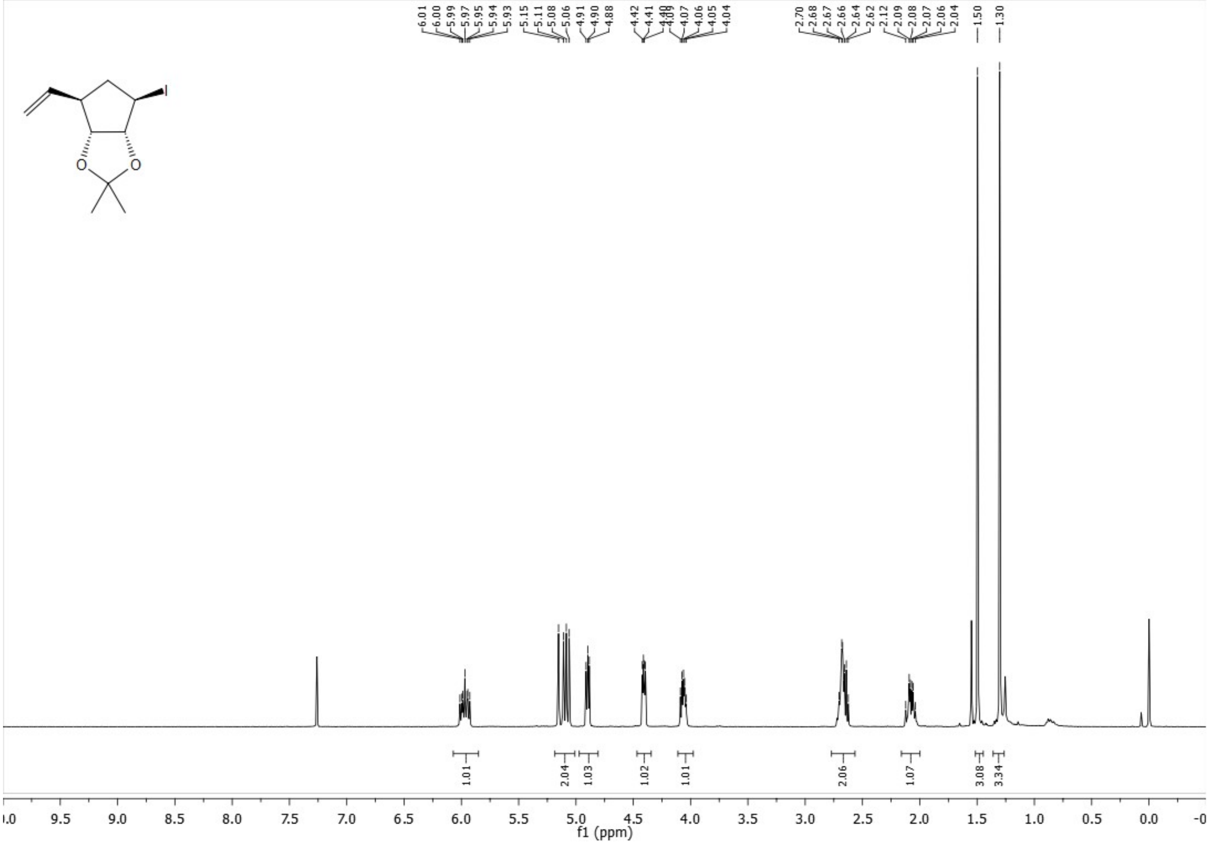

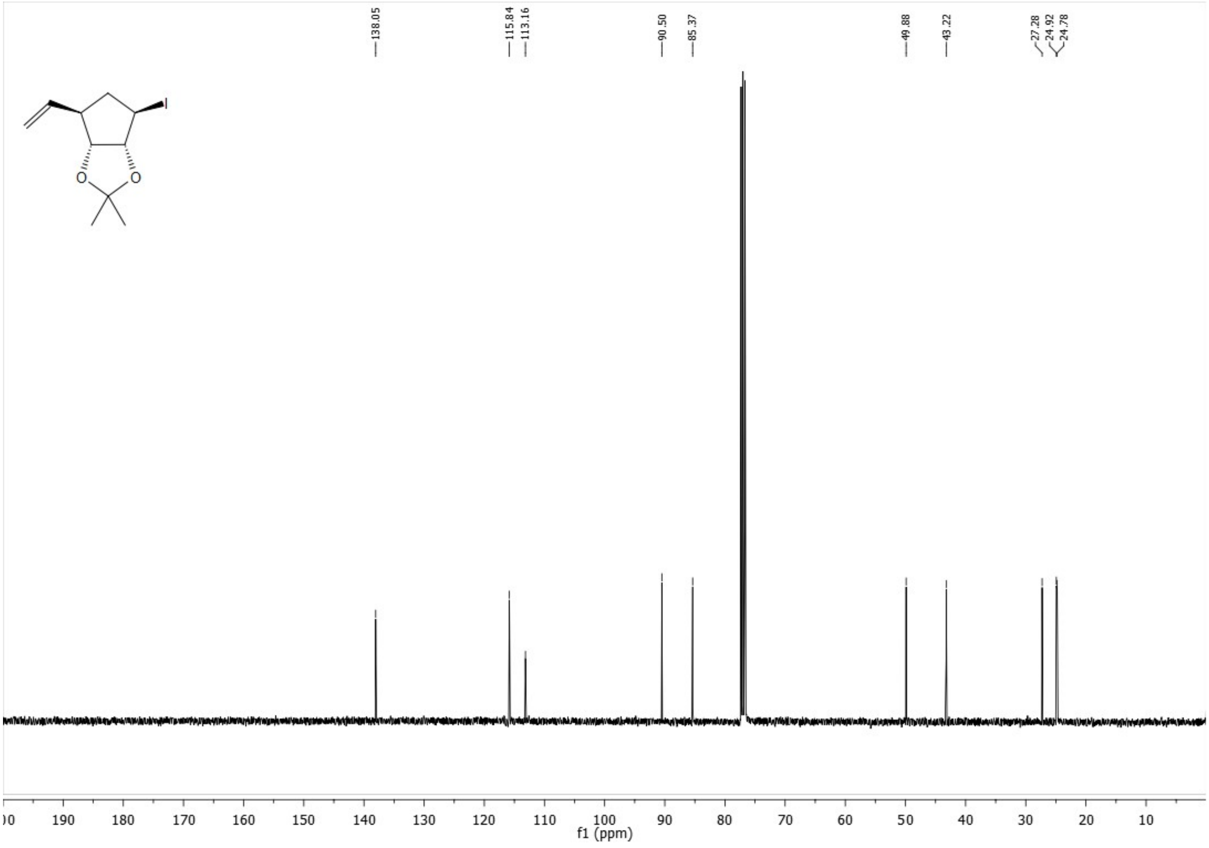
138.05
115.84
113.16
90.50
85.37
49.88
43.22
27.28
24.92
24.78
I
O
O
190
180
170
160
150
140
130
120
110
100
90
80
70
60
50
40
30
20
10
f1 (ppm)

(±)-2-iodocyclopentan-1-ol (6)

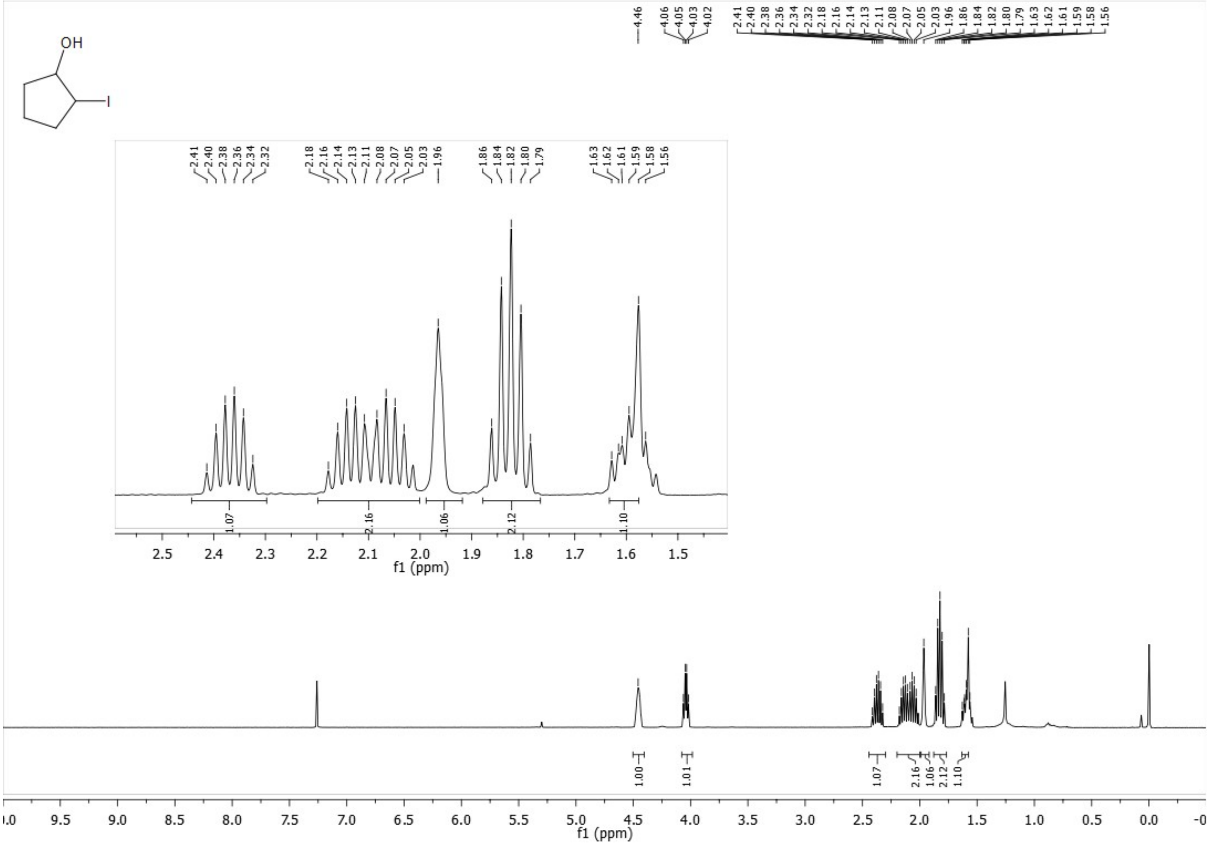

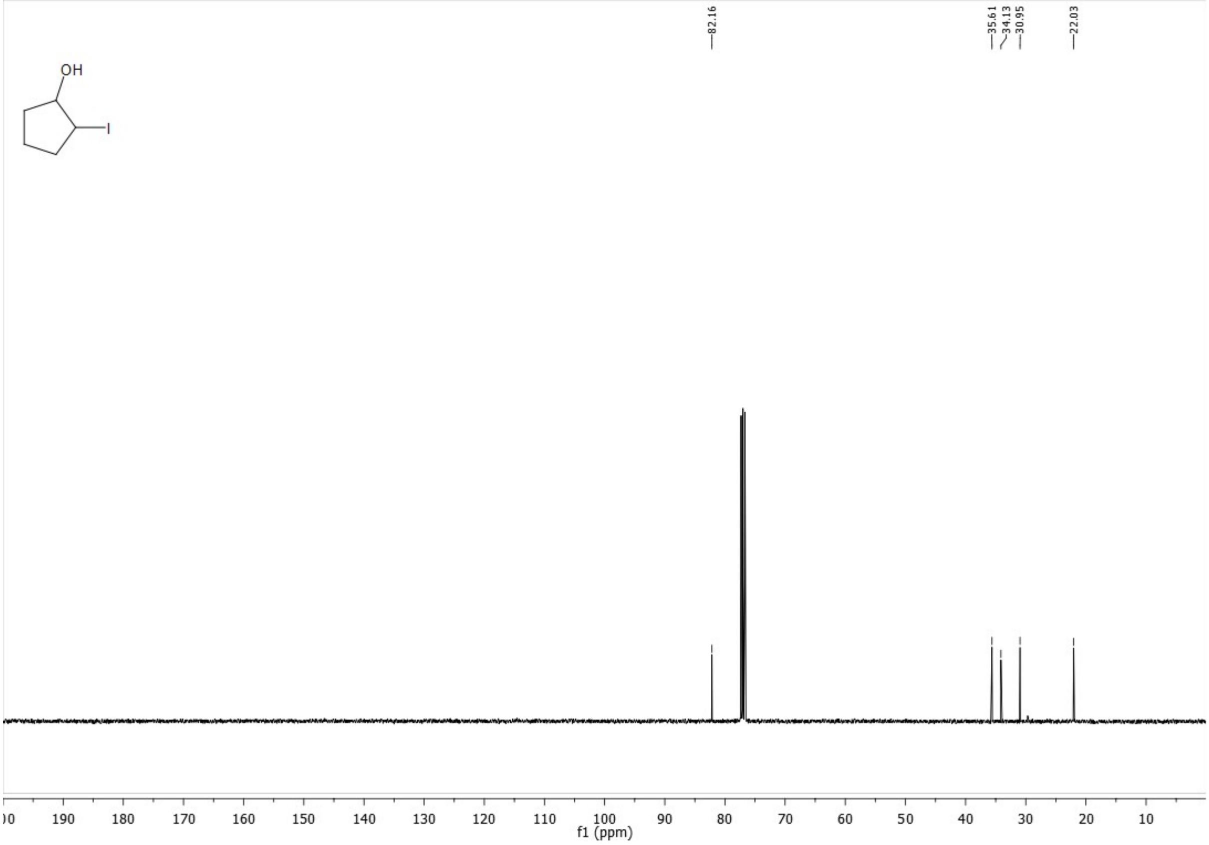

(±)-*tert*-butyl((2-iodocyclopentyl)oxy)dimethylsilane (7)

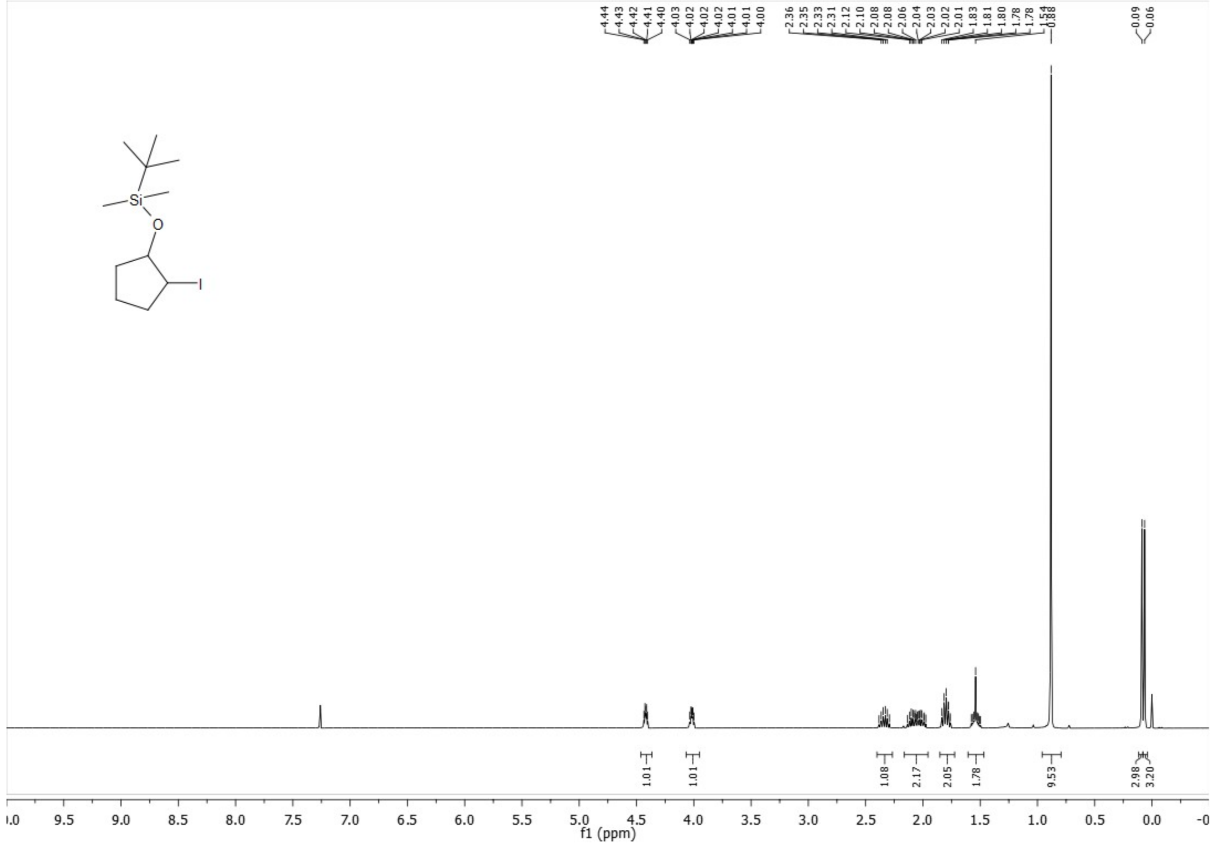

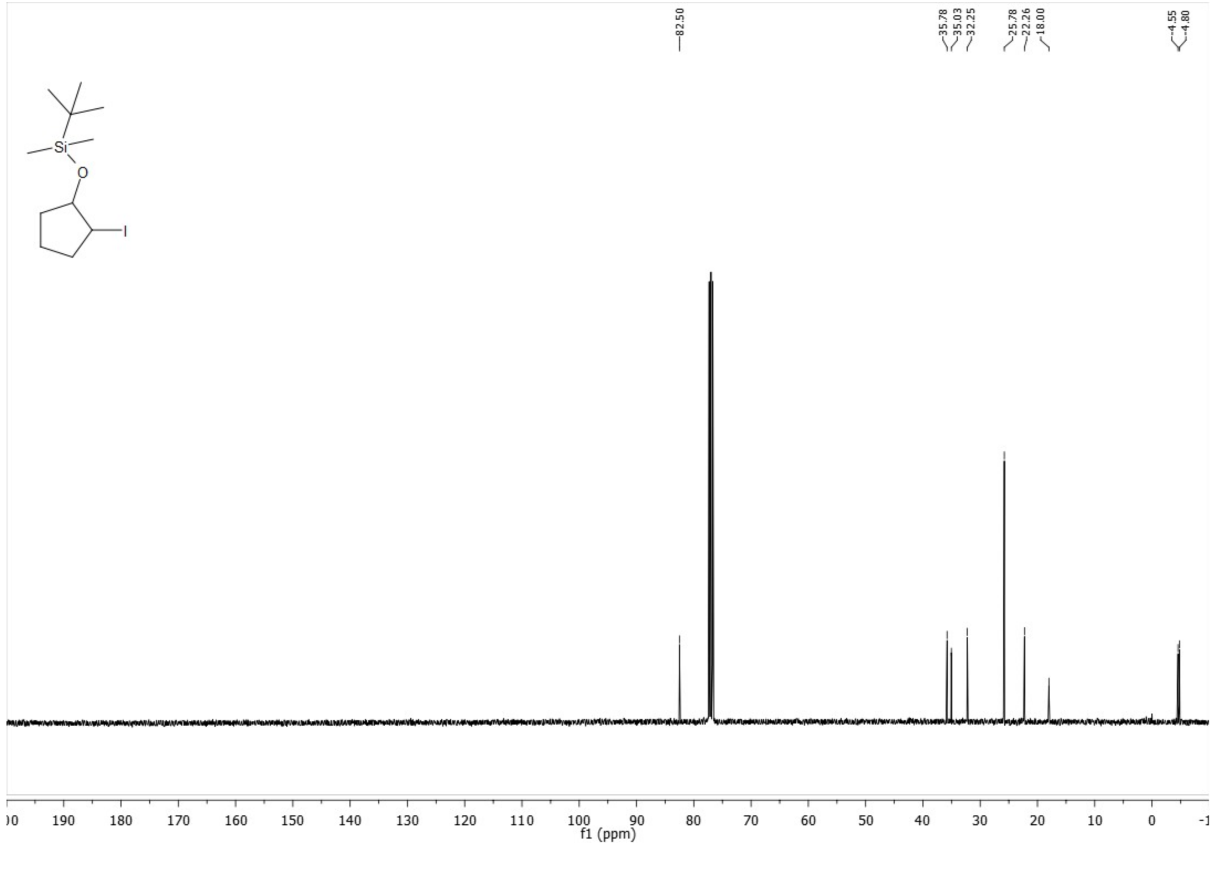

(±)-*tert*-butyldimethyl((2-phenylcyclopentyl)oxy)silane (8)

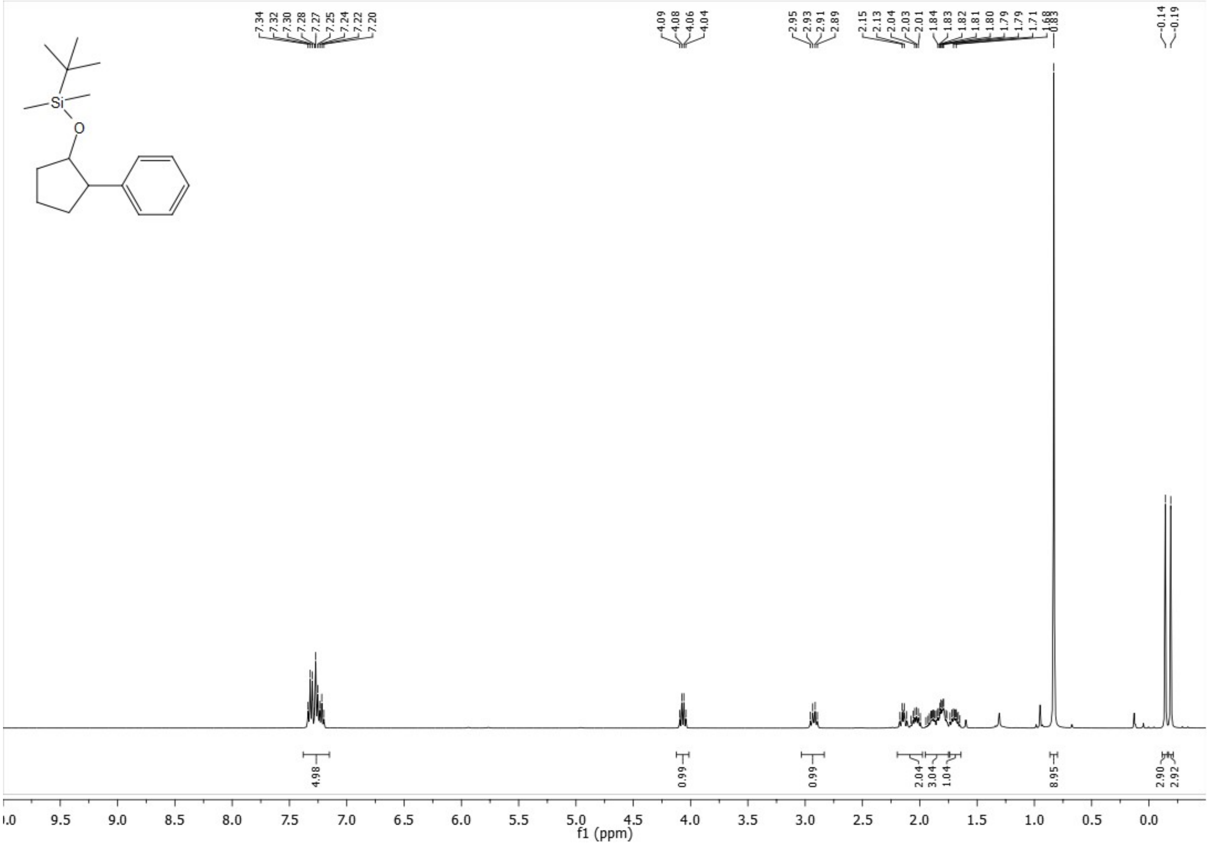

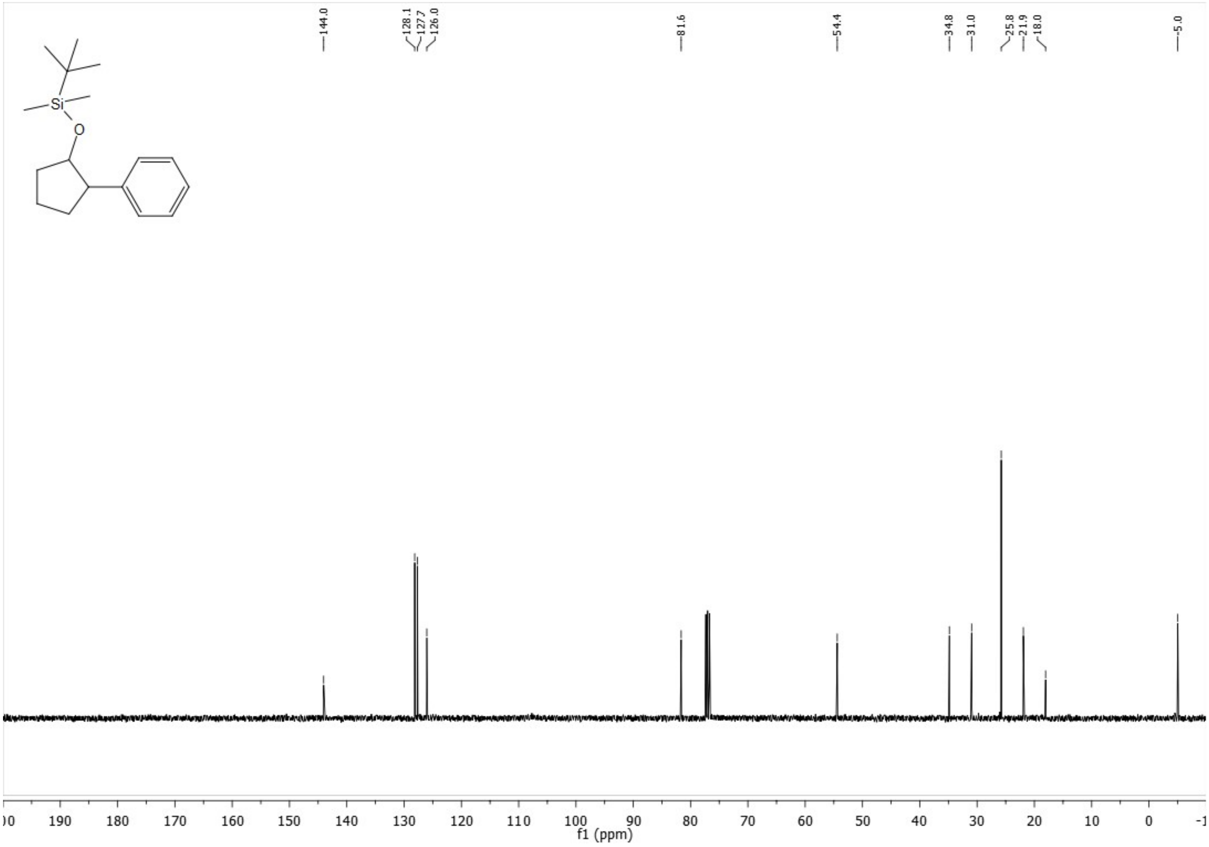

(3aS,4S,6R,6aR)-2,2-dimethyl-4-phenyl-6-vinyltetrahydro-4H-cyclopenta[d][1,3]dioxole (9a)

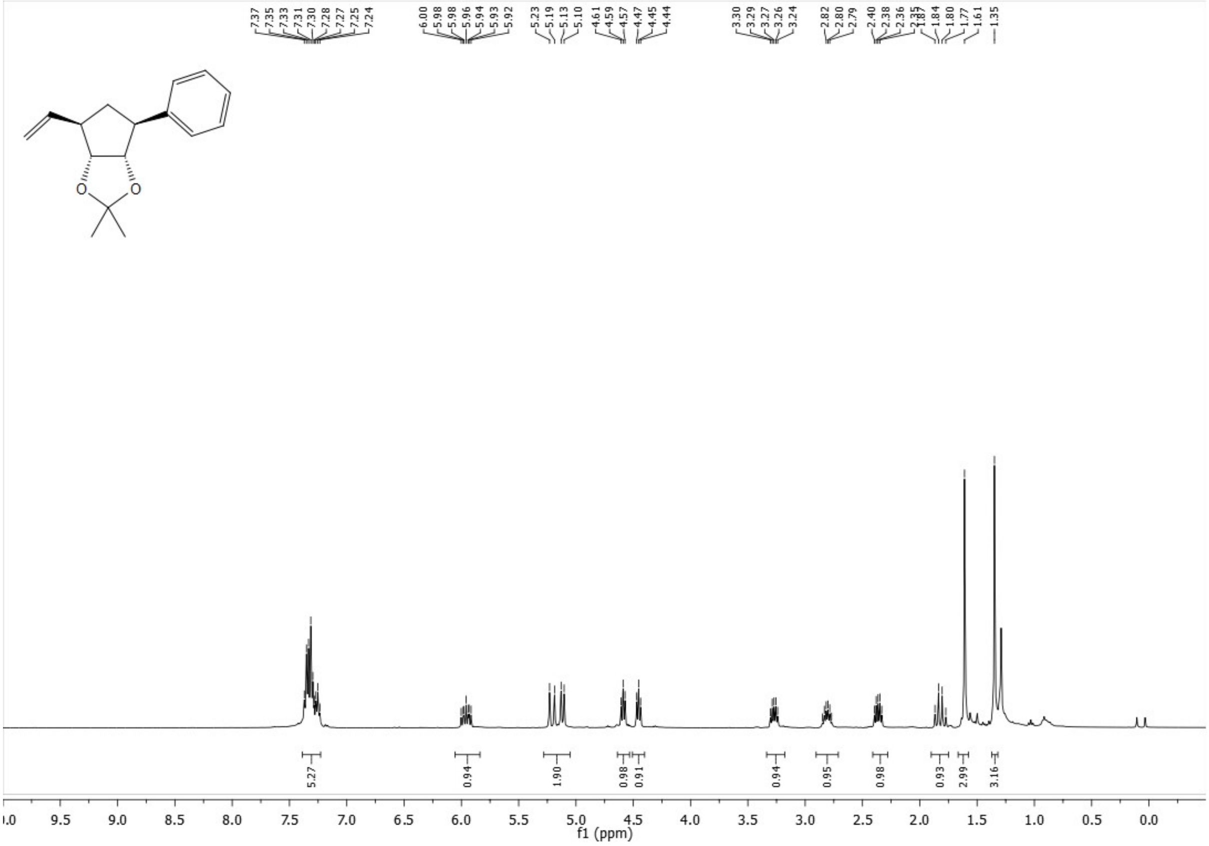

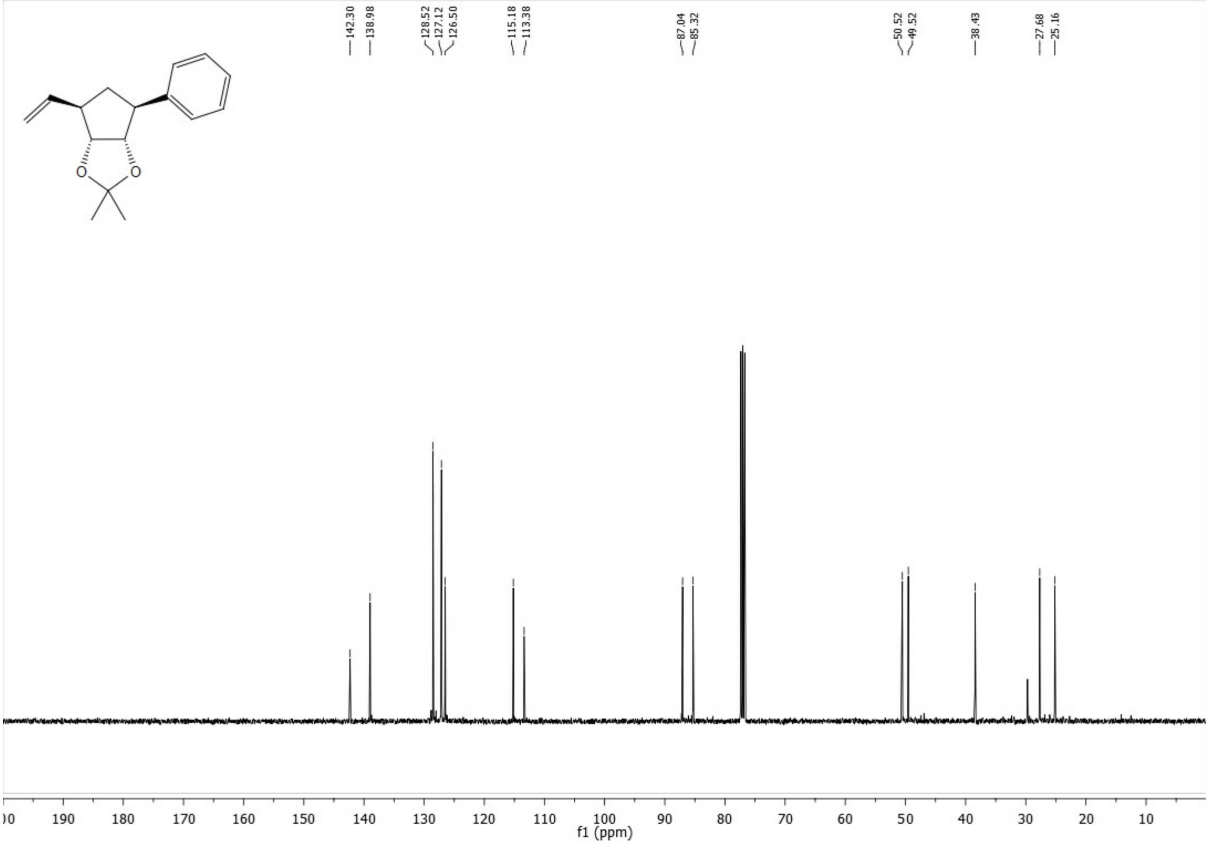

(3aS,4S,6R,6aR)-4-(4-fluorophenyl)-2,2-dimethyl-6-vinyltetrahydro-4H-cyclopenta[d][1,3]dioxole (9b)

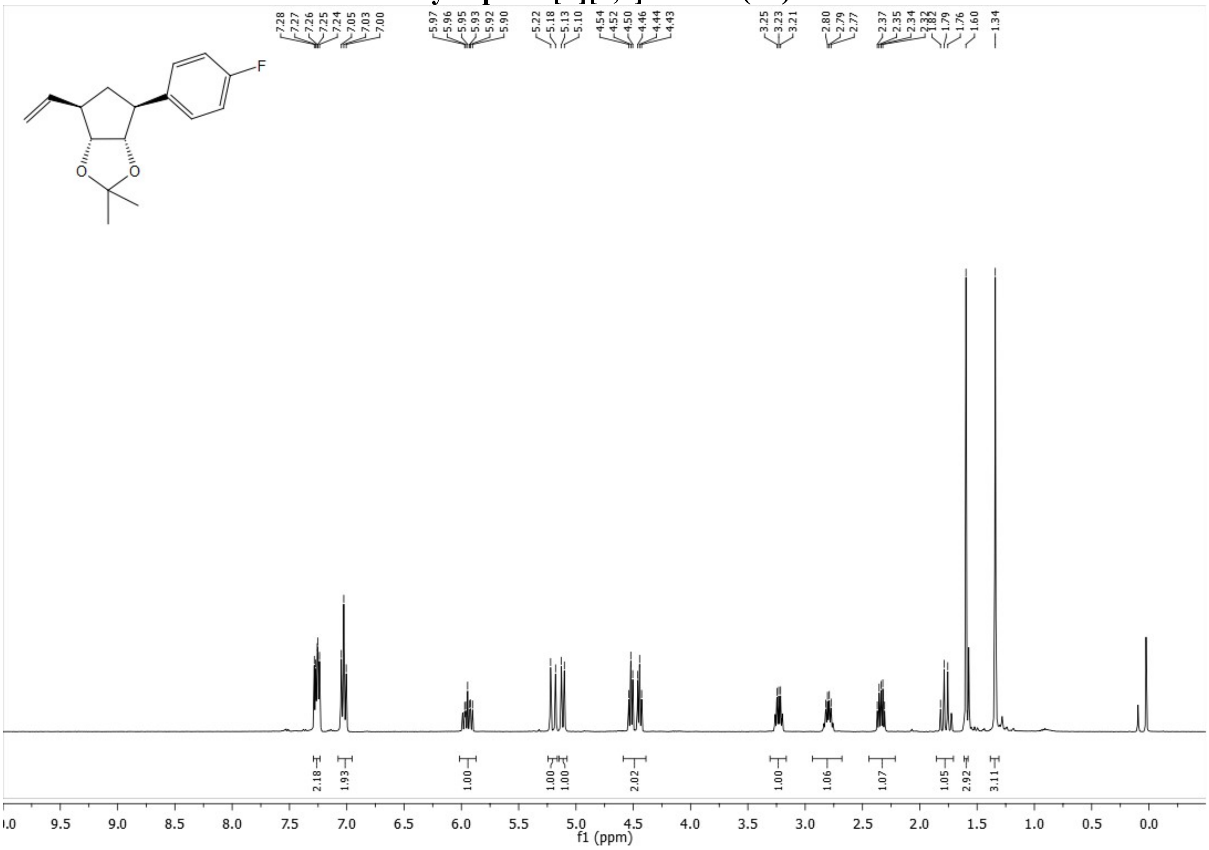

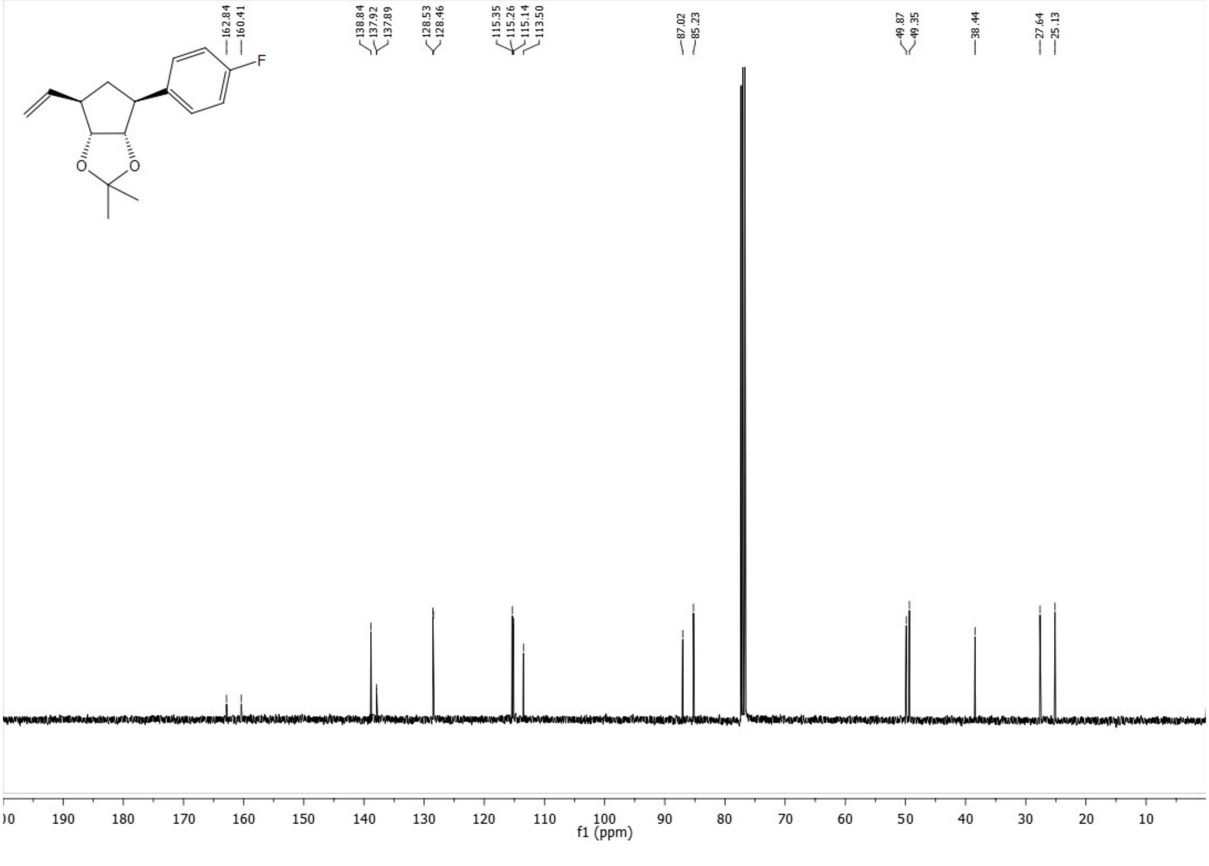

(3aS,4S,6R,6aR)-4-(4-methoxyphenyl)-2,2-dimethyl-6-vinyltetrahydro-4H-cyclopenta[d][1,3]dioxole (9c)

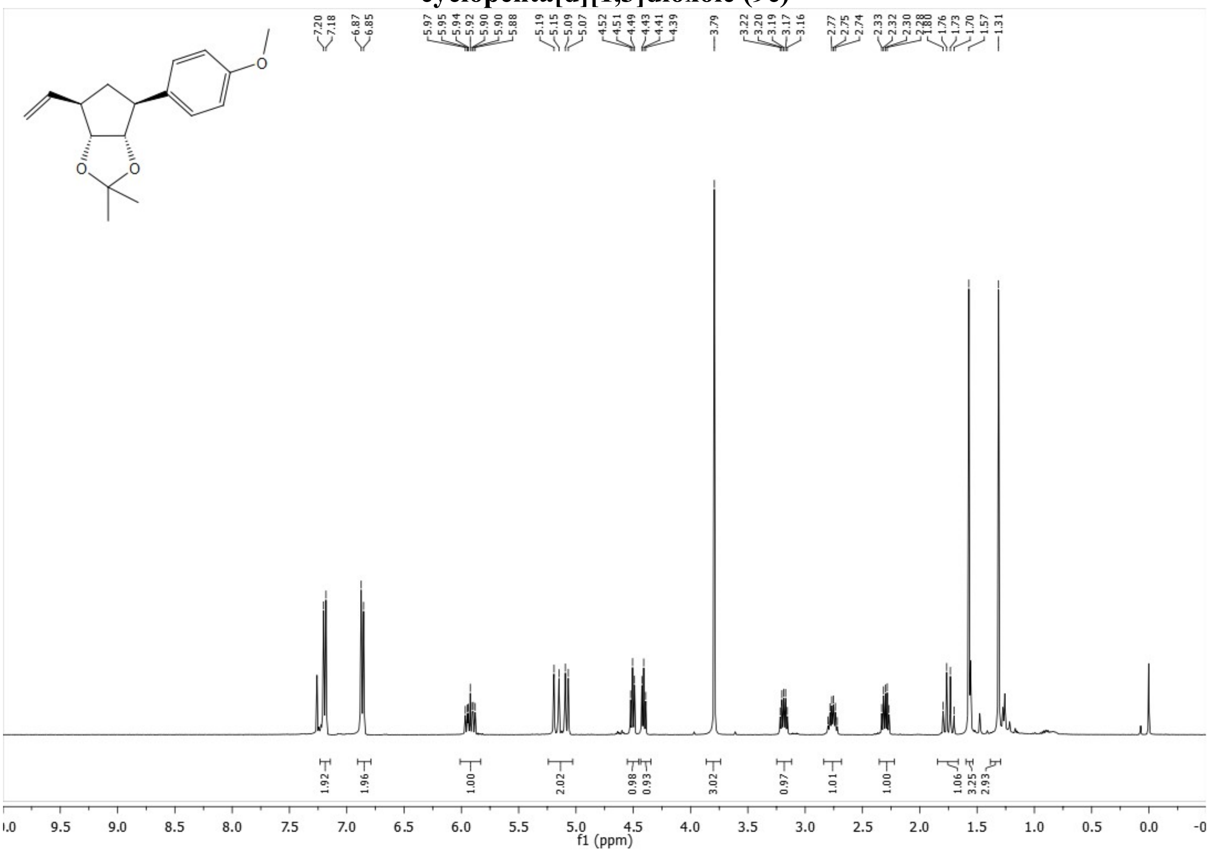

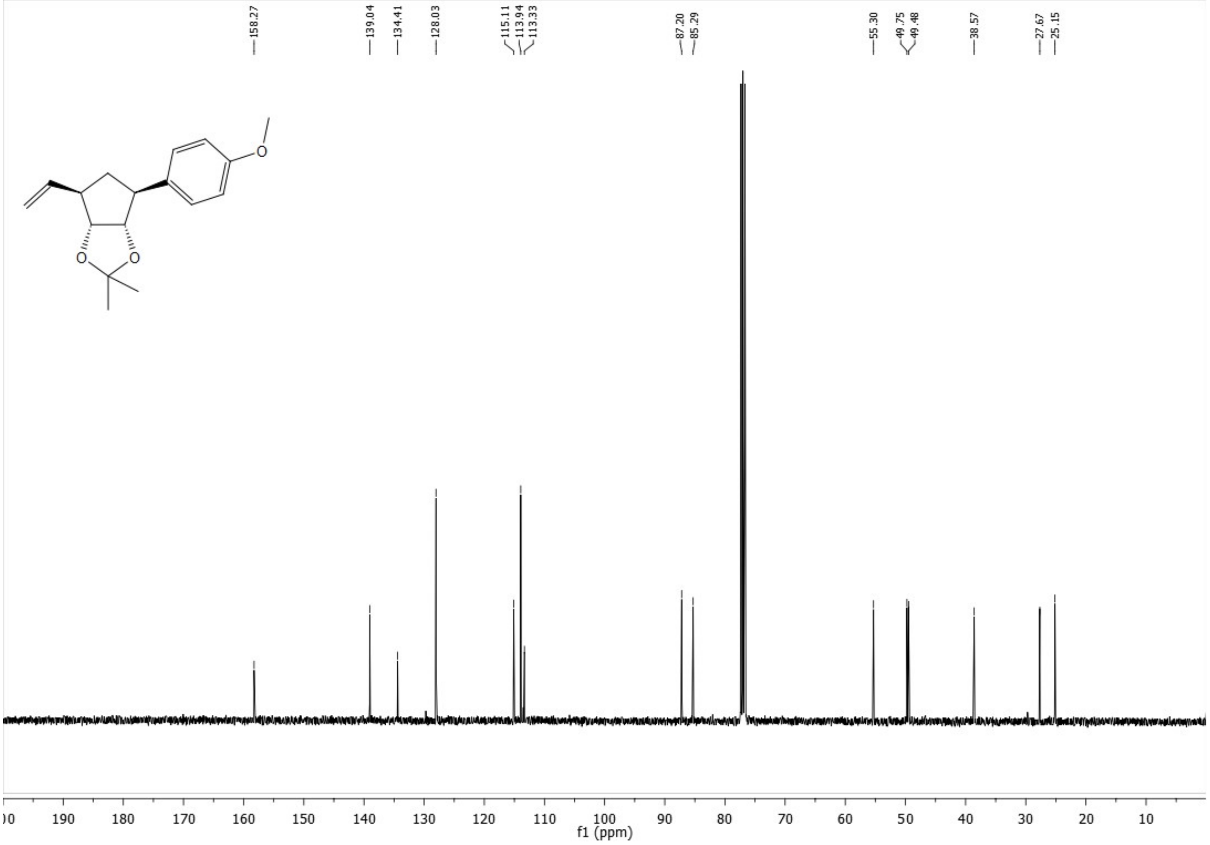

(3aS,4S,6R,6aR)-2,2-dimethyl-4-(4-(trifluoromethoxy)phenyl)-6-vinyltetrahydro-4H-cyclopenta[d][1,3]dioxole (9d)

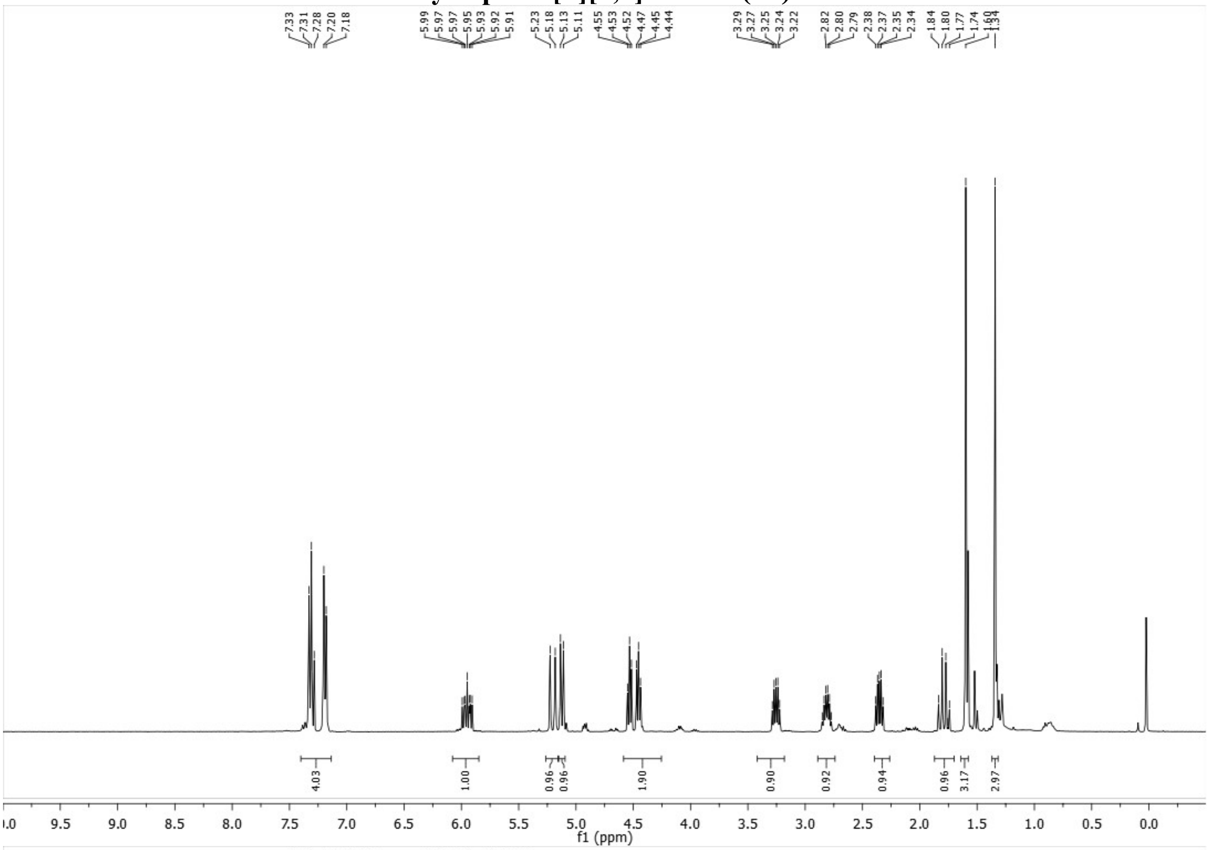

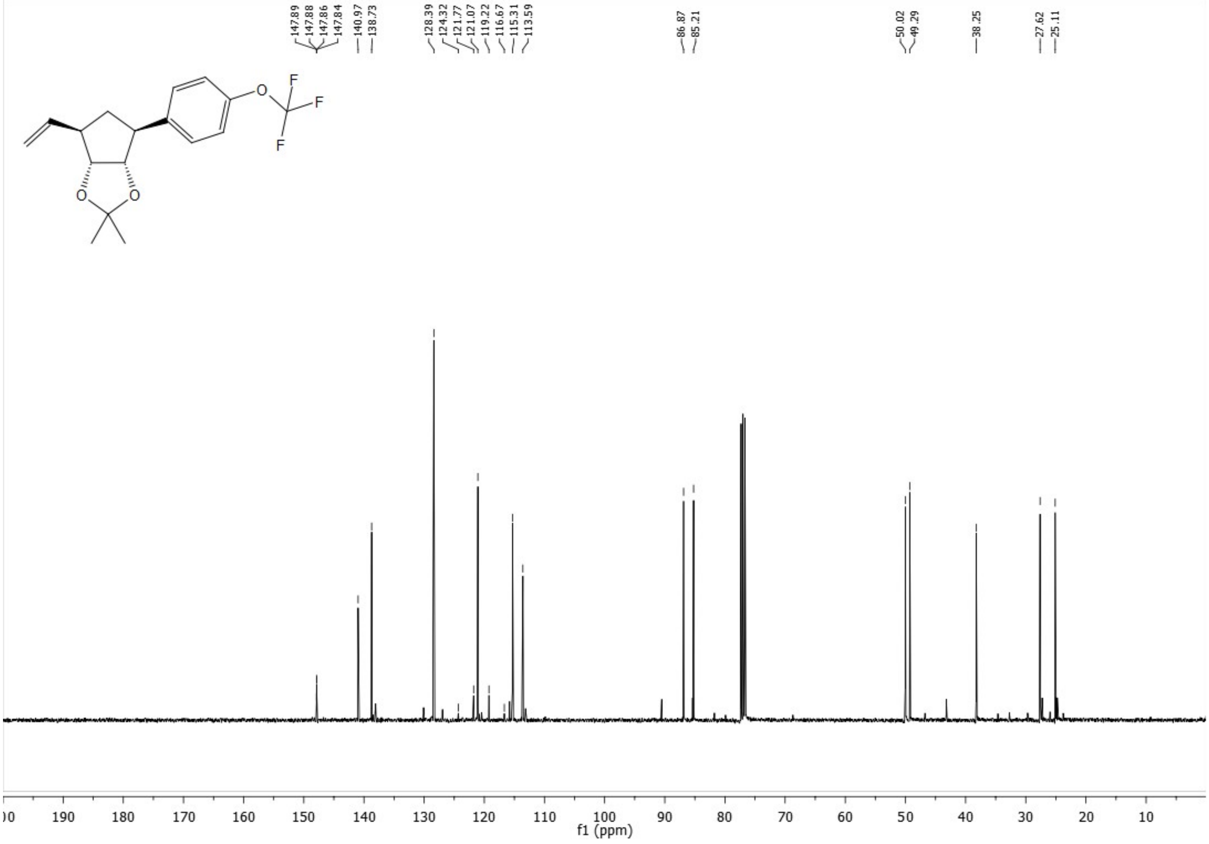

3-((3aS,4S,6R,6aR)-2,2-dimethyl-6-vinyltetrahydro-4H-cyclopenta[d][1,3]dioxol-4-yl)-2-methylpyridine (9e)

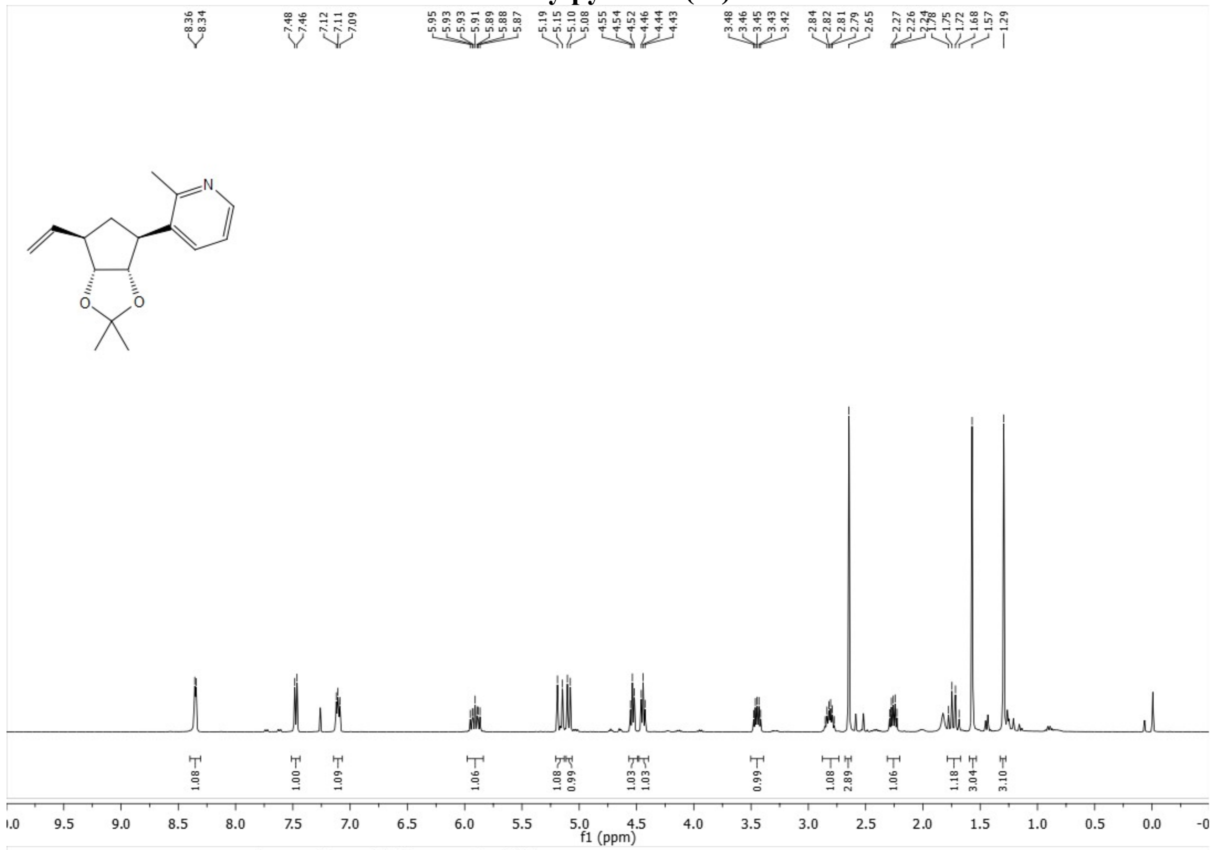

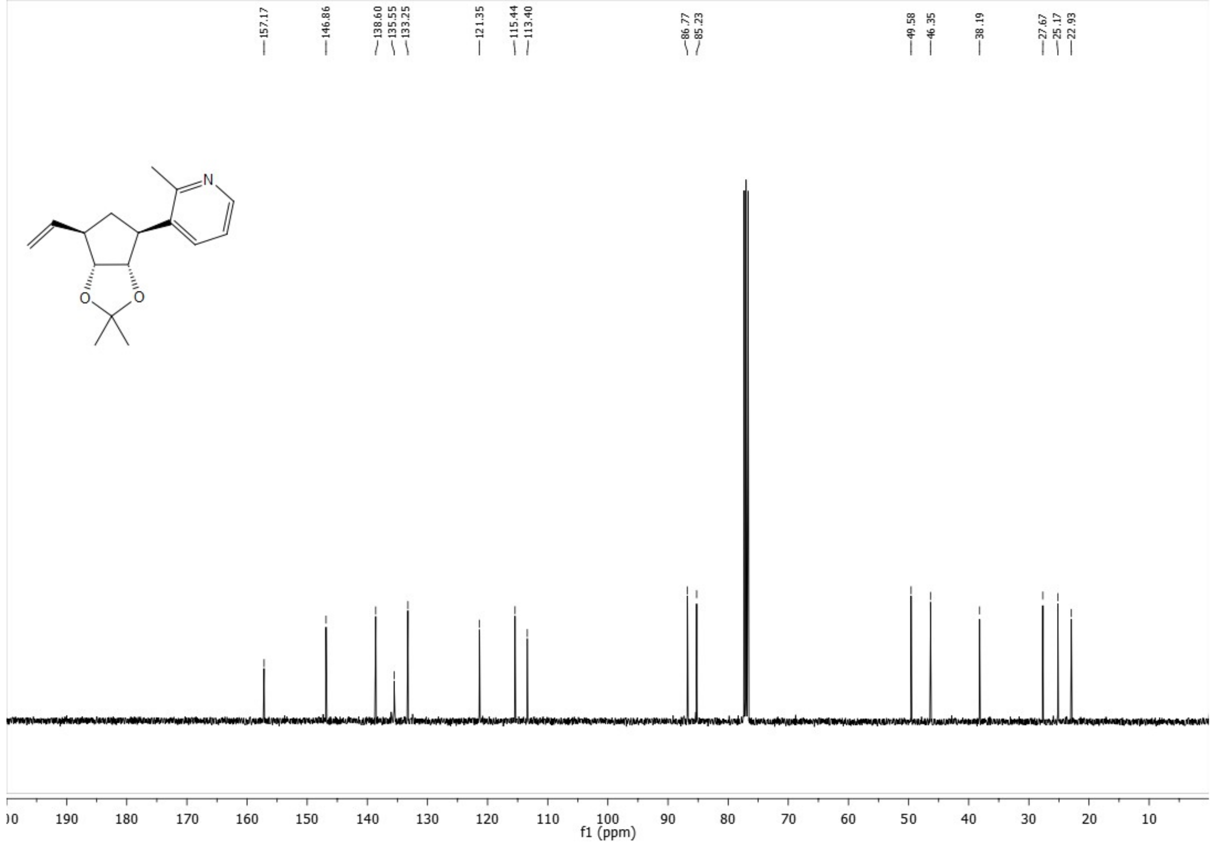

(3aS,4S,6R,6aR)-2,2-dimethyl-4-(naphthalen-2-yl)-6-vinyltetrahydro-4H-cyclopenta[d][1,3]dioxole (9f)

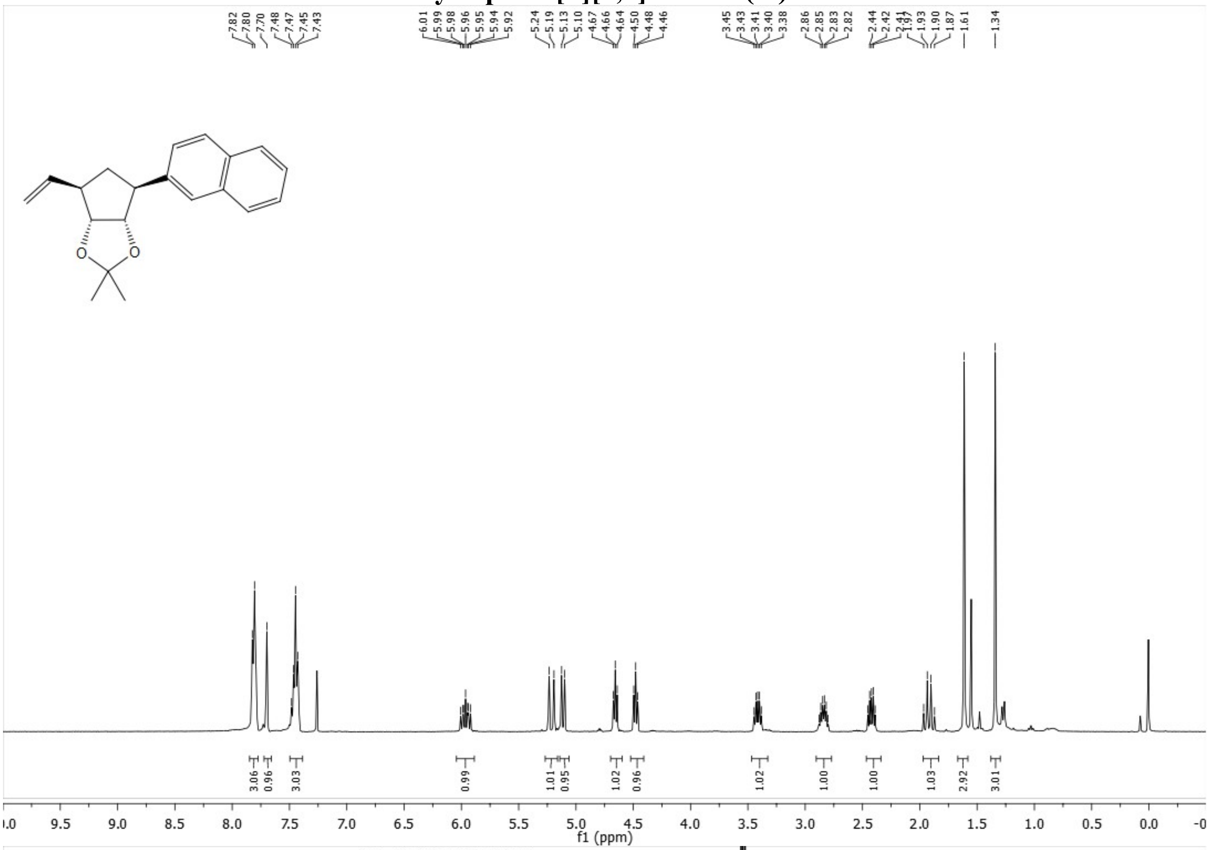

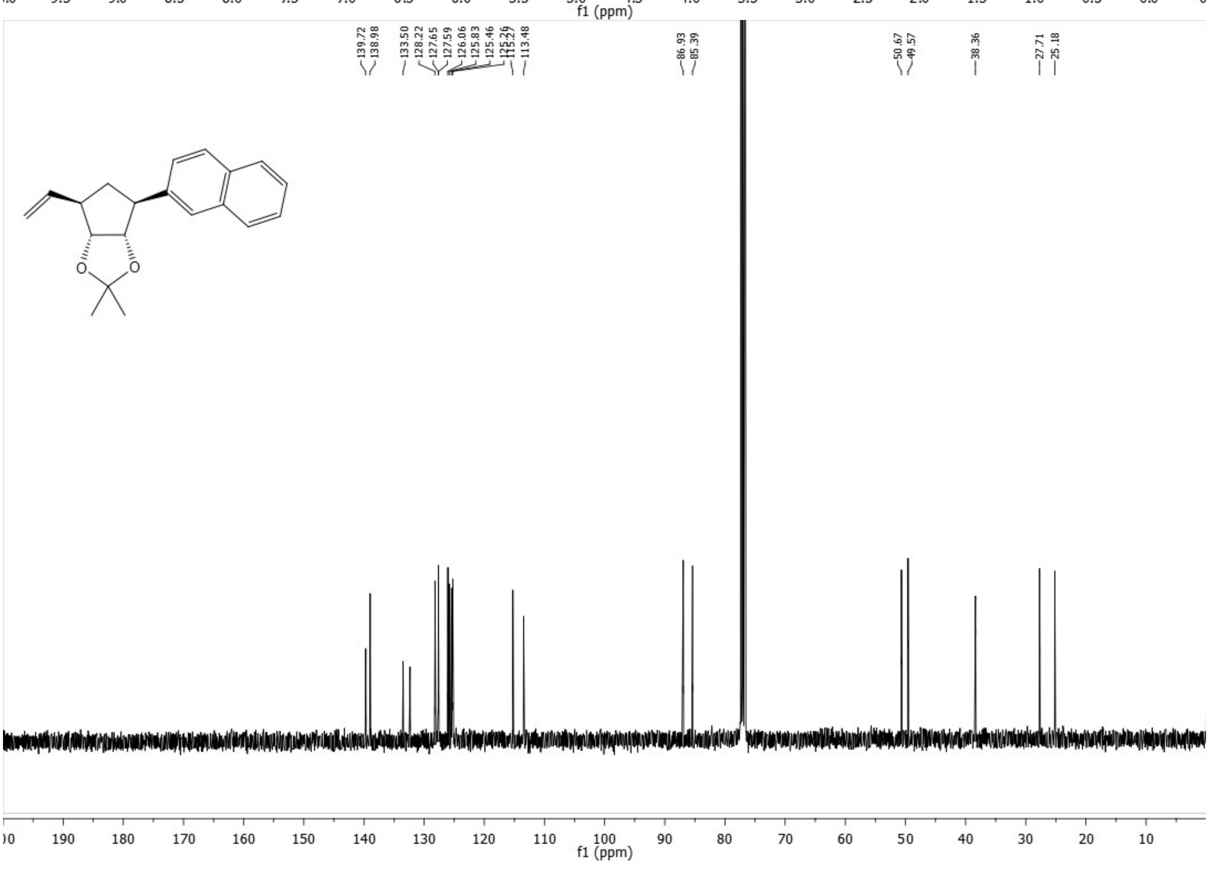

(3aS,4S,6R,6aR)-4-(3-methoxynaphthalen-2-yl)-2,2-dimethyl-6-vinyltetrahydro-4H-cyclopenta[d][1,3]dioxole (9g)

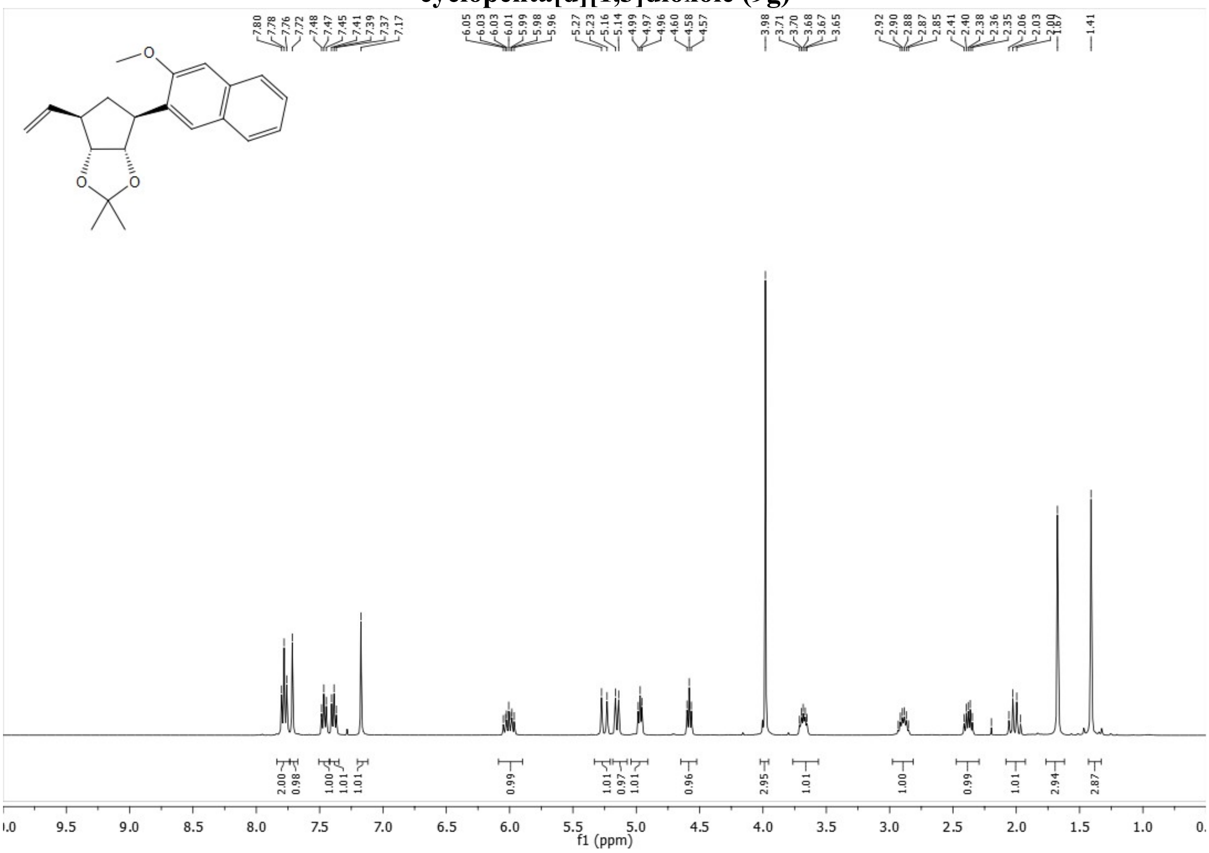

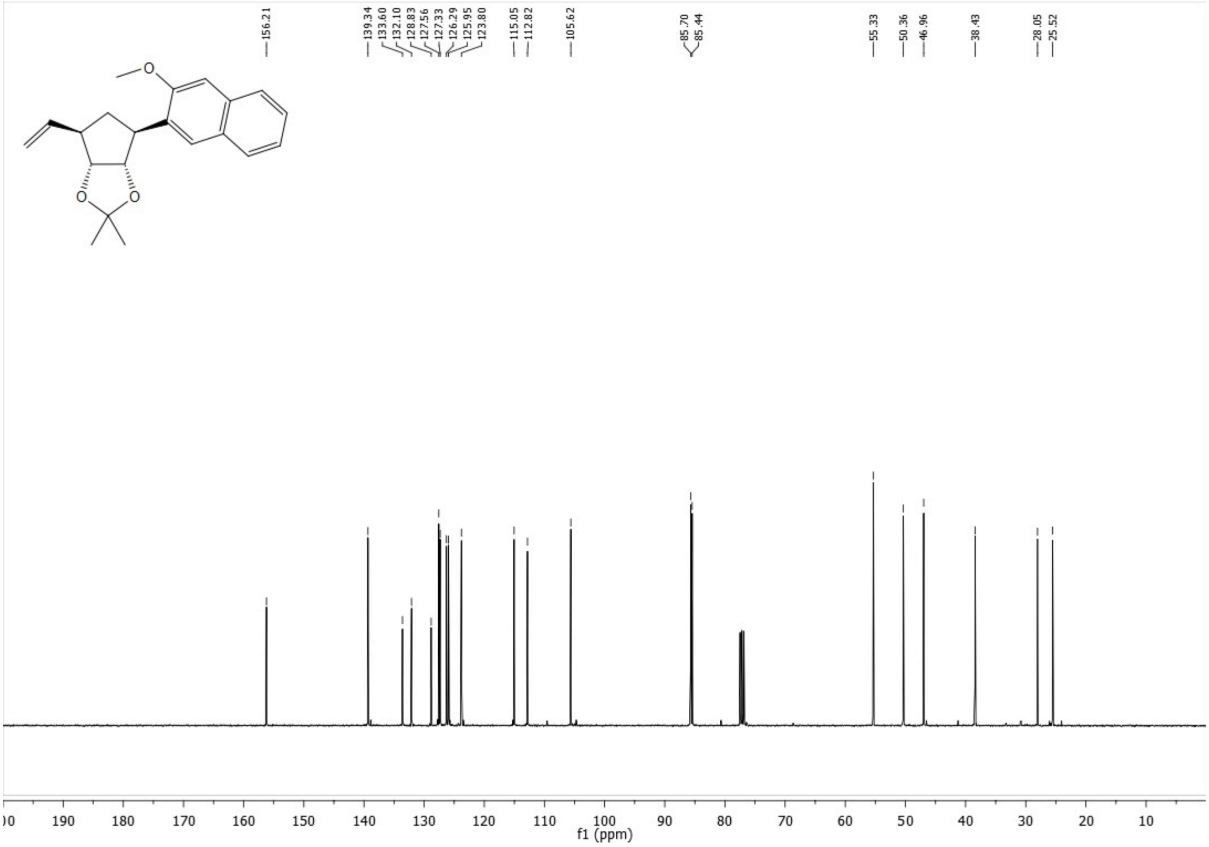

(3aS,4S,6R,6aR)-2,2-dimethyl-4-(naphthalen-1-yl)-6-vinyltetrahydro-4H-cyclopenta[d][1,3]dioxole (9h)

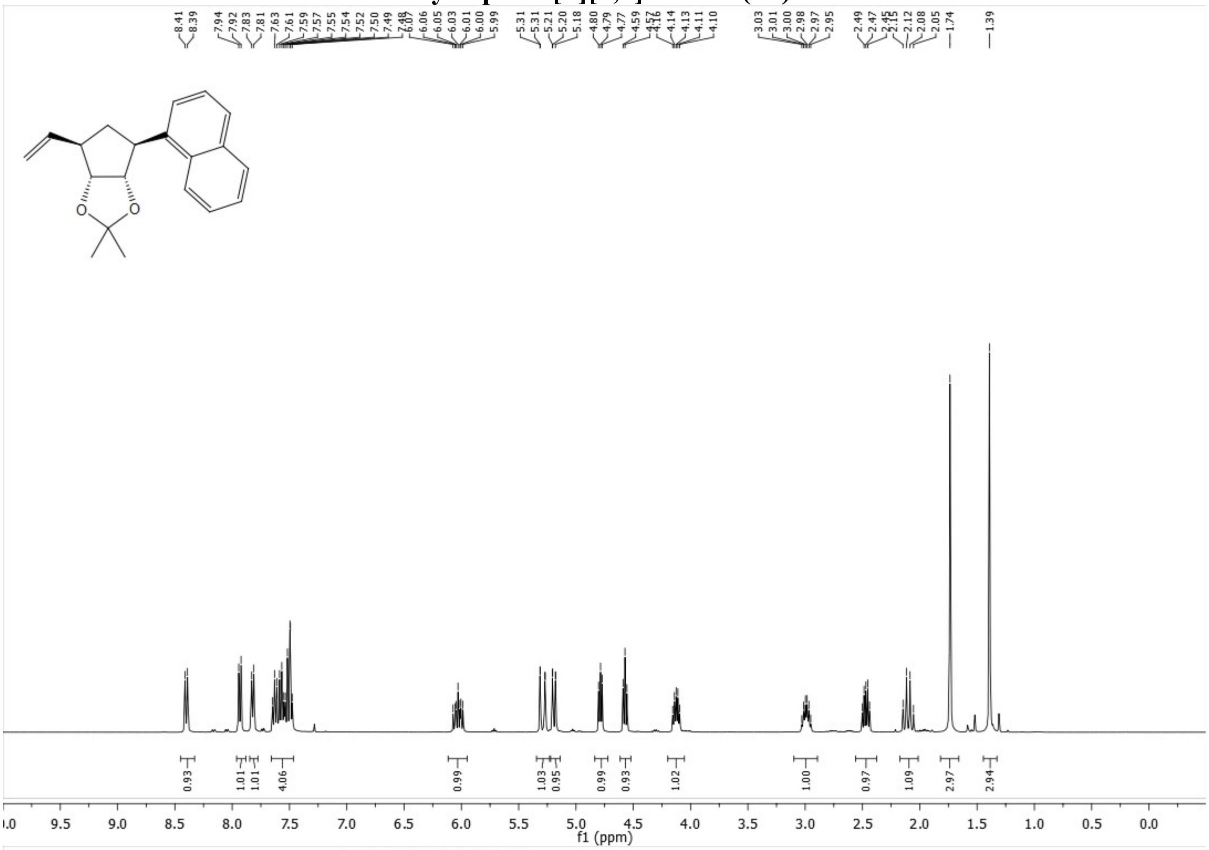

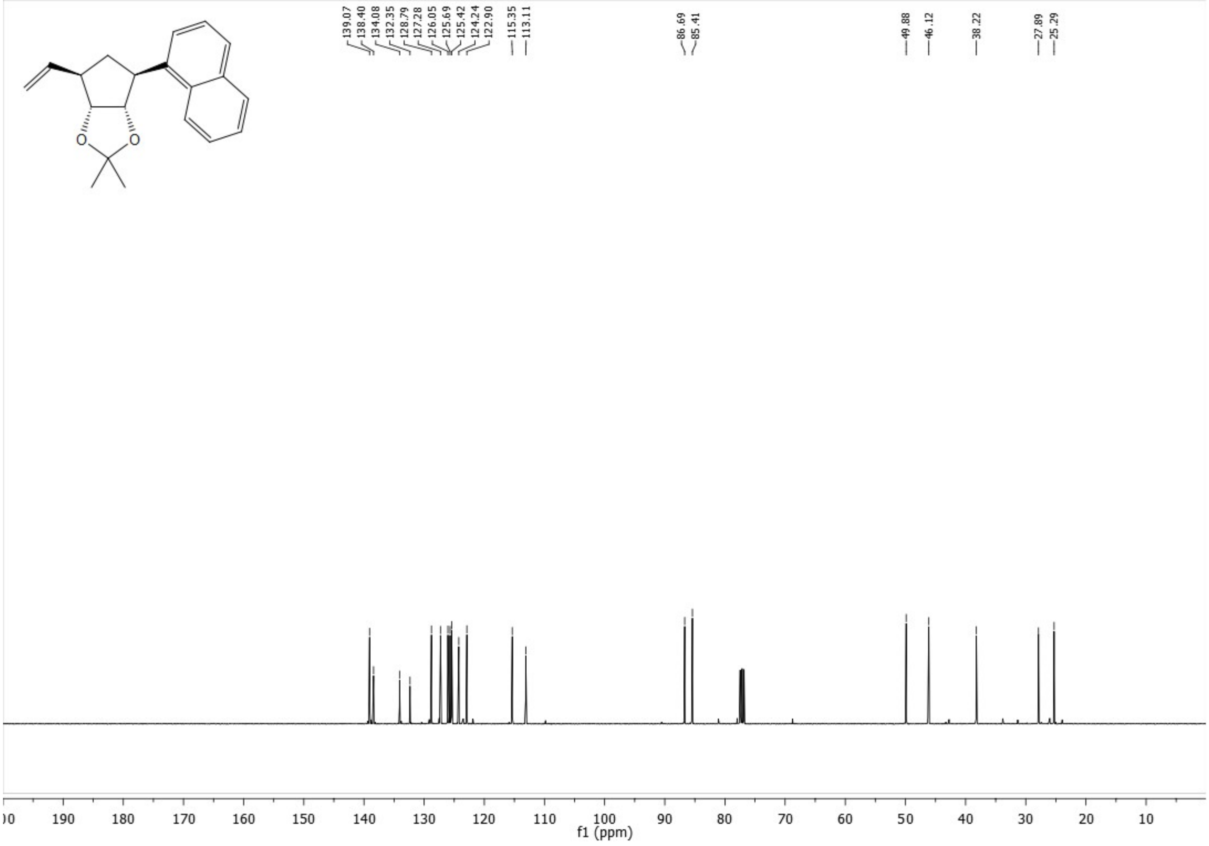

(3aS,4S,6R,6aR)-4-(2,4-difluoro-5-methylphenyl)-2,2-dimethyl-6-vinyltetrahydro-4H-cyclopenta[d][1,3]dioxole (9i)

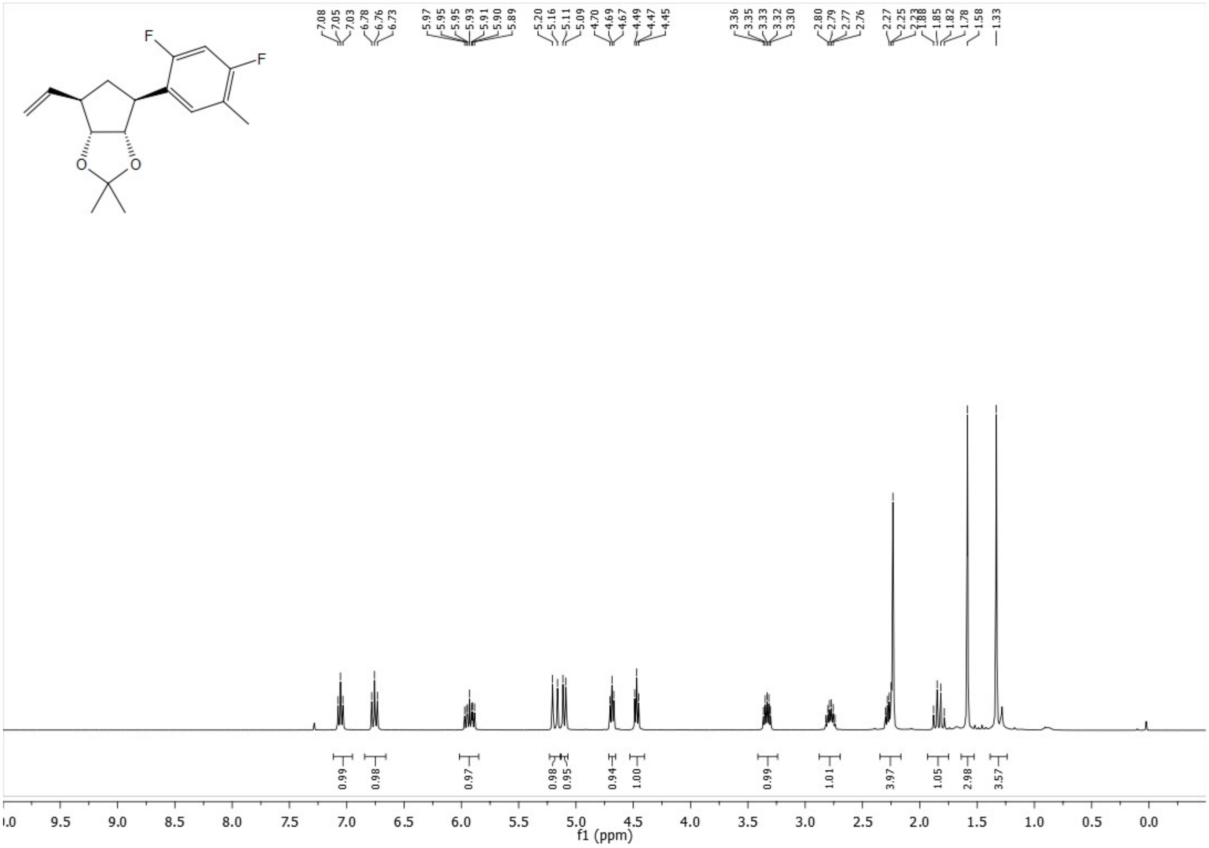

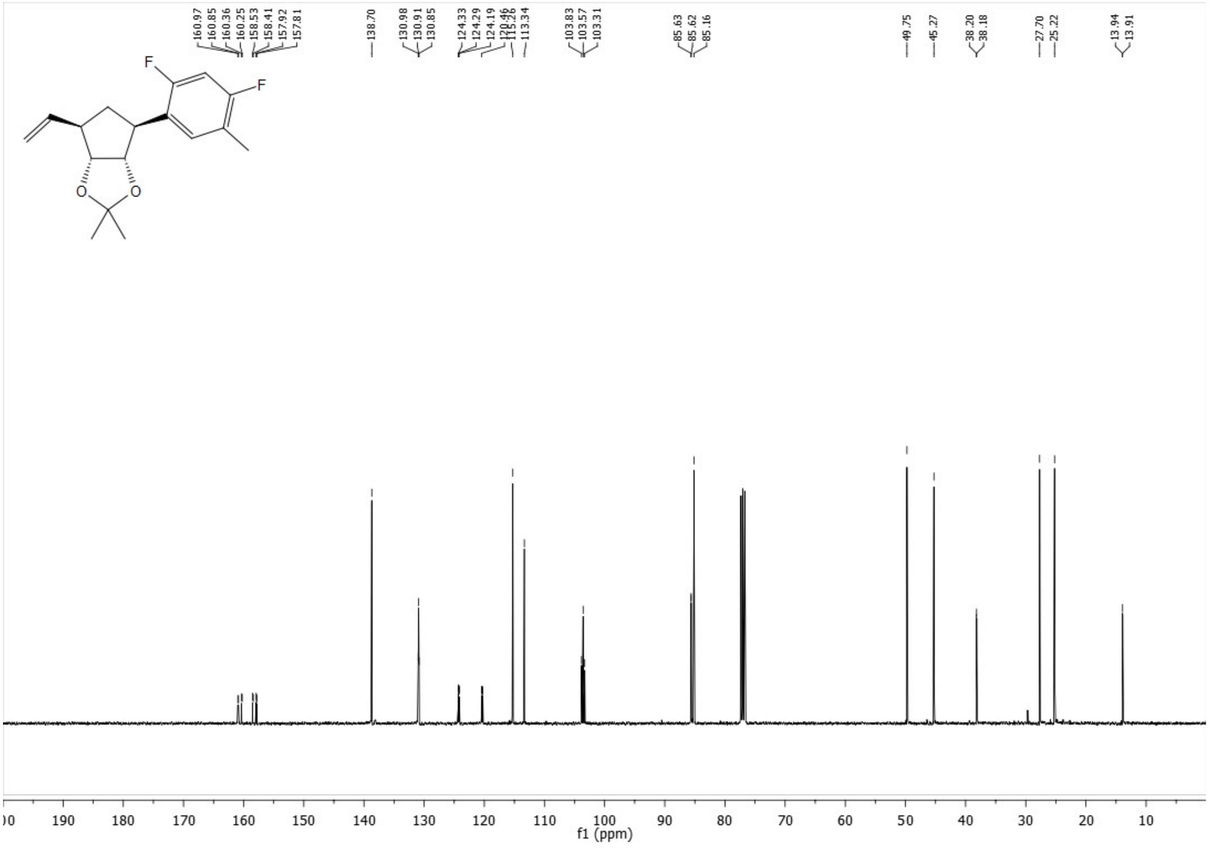

3-((3aS,4S,6R,6aR)-2,2-dimethyl-6-vinyltetrahydro-4H-cyclopenta[d][1,3]dioxol-4-yl)pyridine (9j)

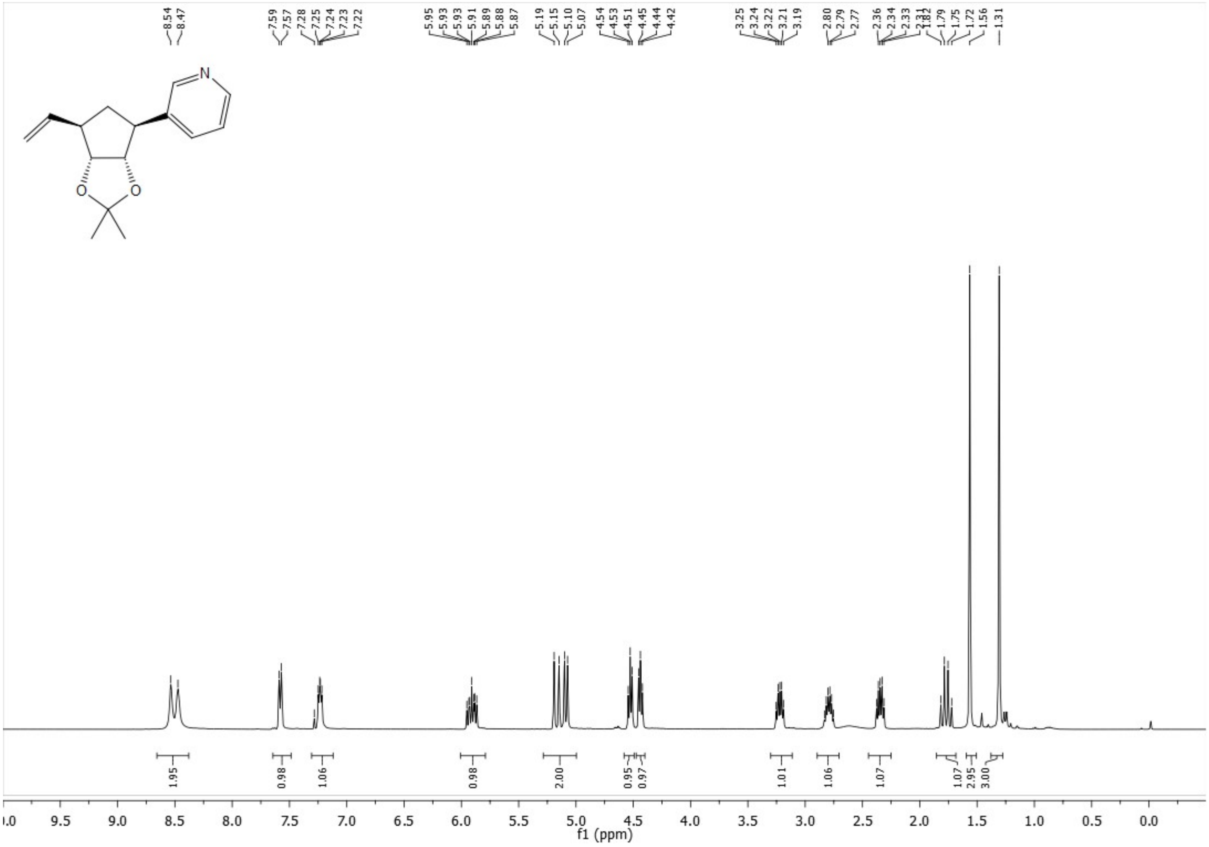

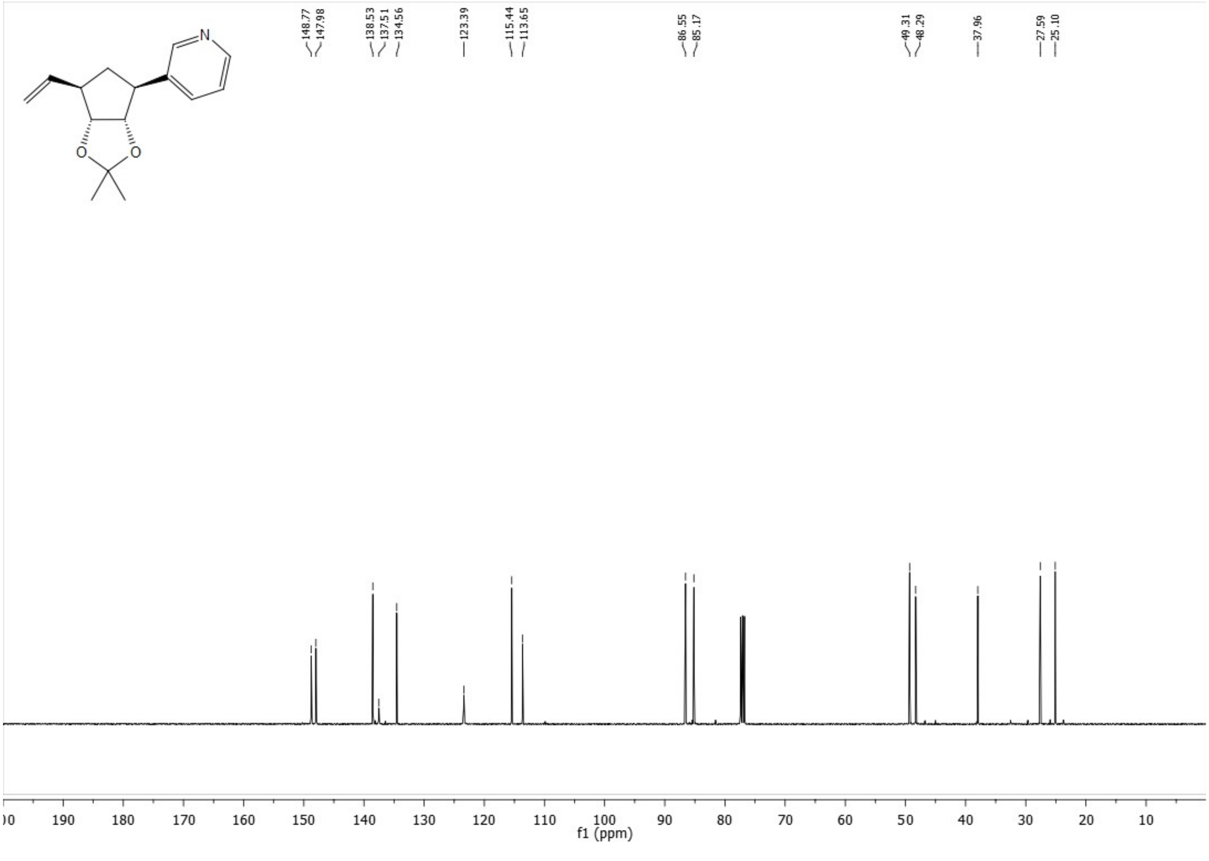

5-((3aS,4S,6R,6aR)-2,2-dimethyl-6-vinyltetrahydro-4H-cyclopenta[d][1,3]dioxol-4-yl)-2-fluoropyridine (9k)

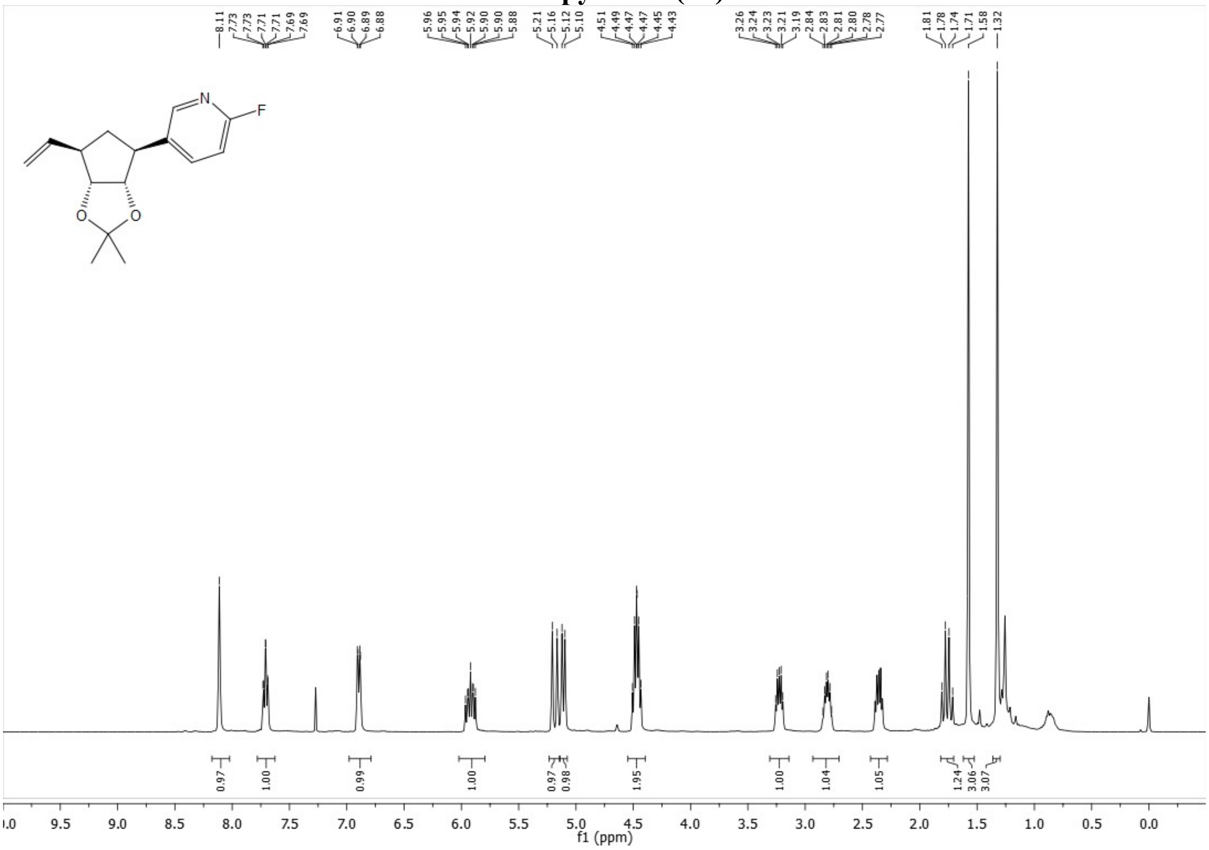

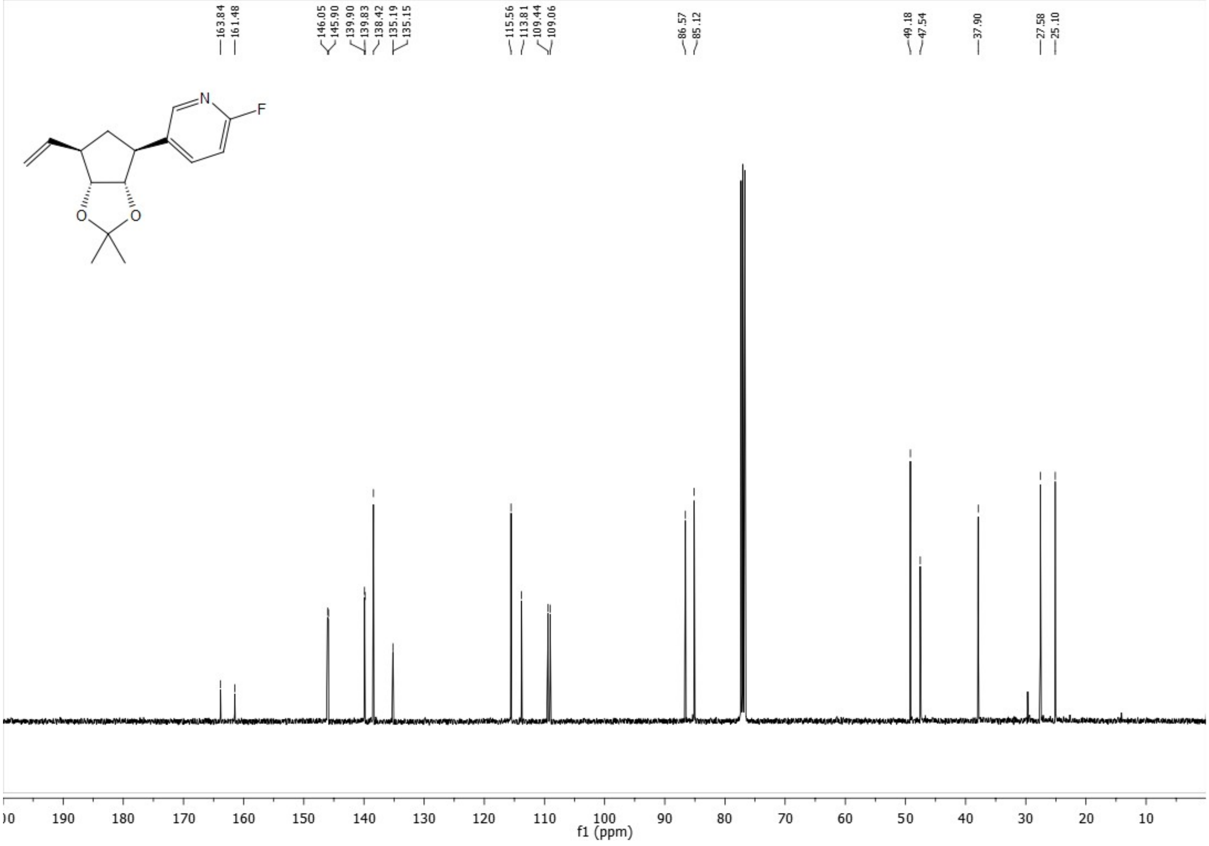

4-((3aS,4S,6R,6aR)-2,2-dimethyl-6-vinyltetrahydro-4H-cyclopenta[d][1,3]dioxol-4-yl)benzonitrile (9l)

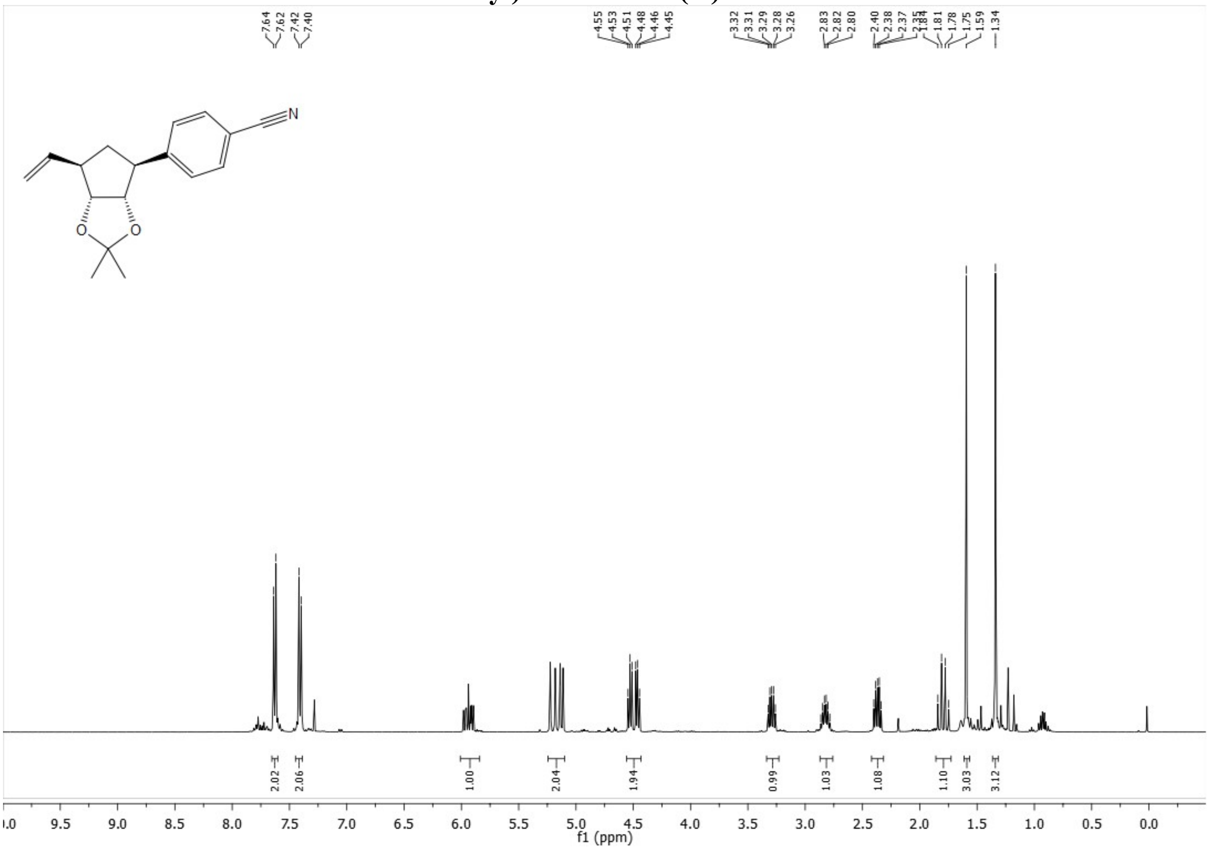

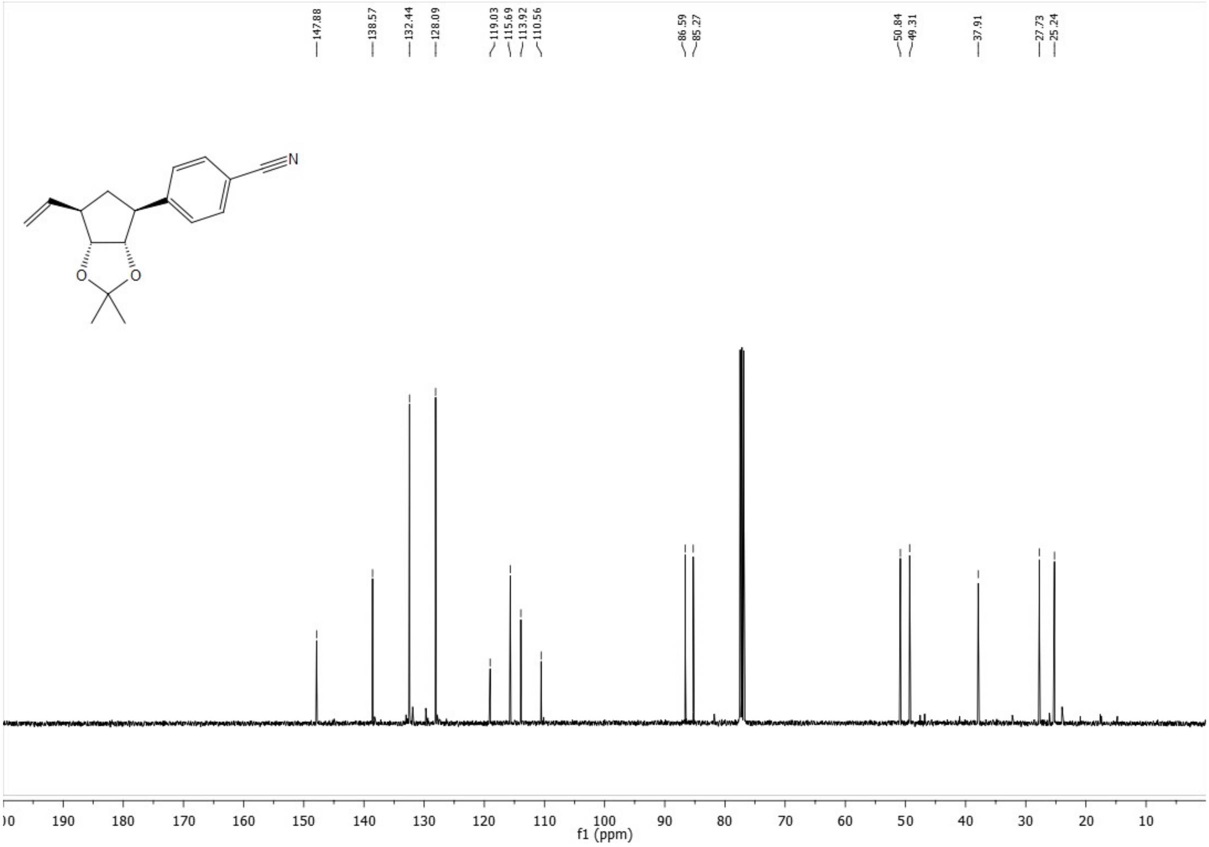

((3aR,4R,6S,6aS)-2,2-dimethyl-6-phenyltetrahydro-4H-cyclopenta[d][1,3]dioxol-4-yl)methanol (10a)

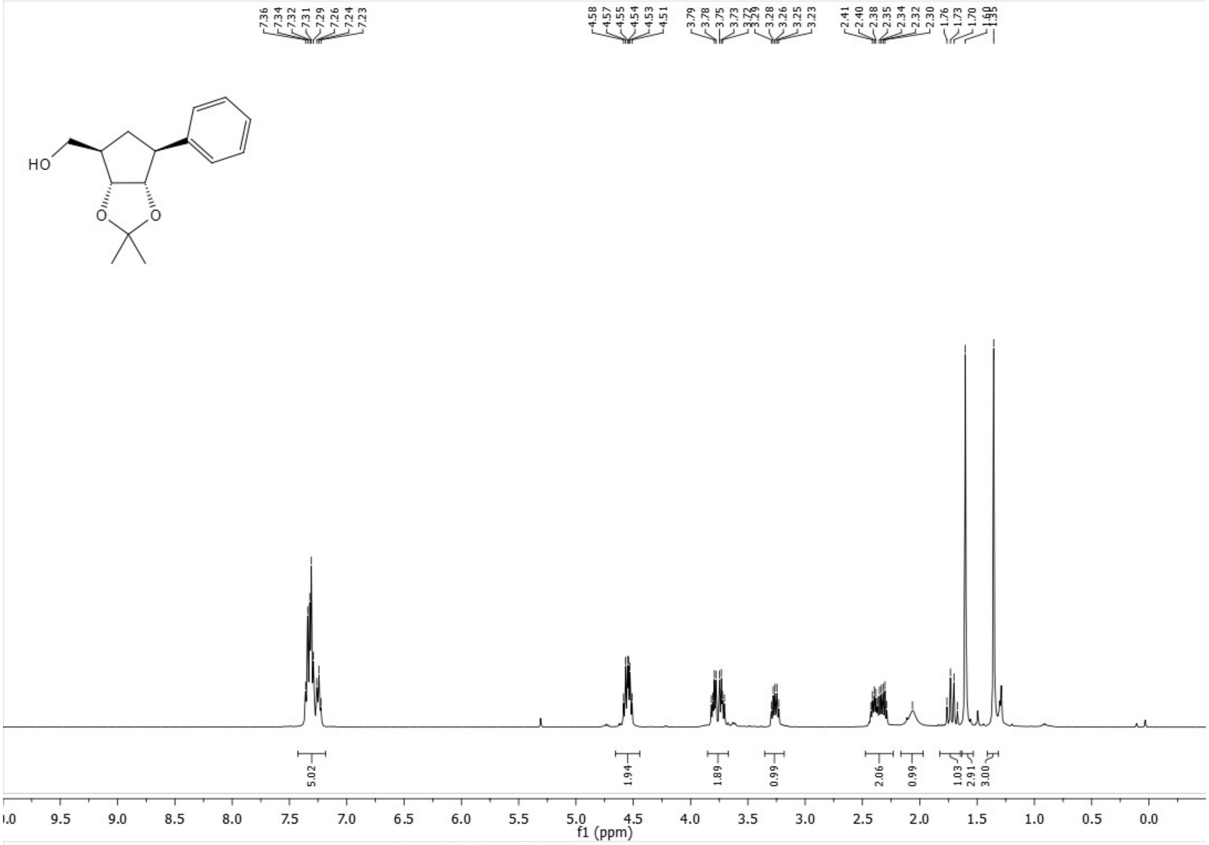

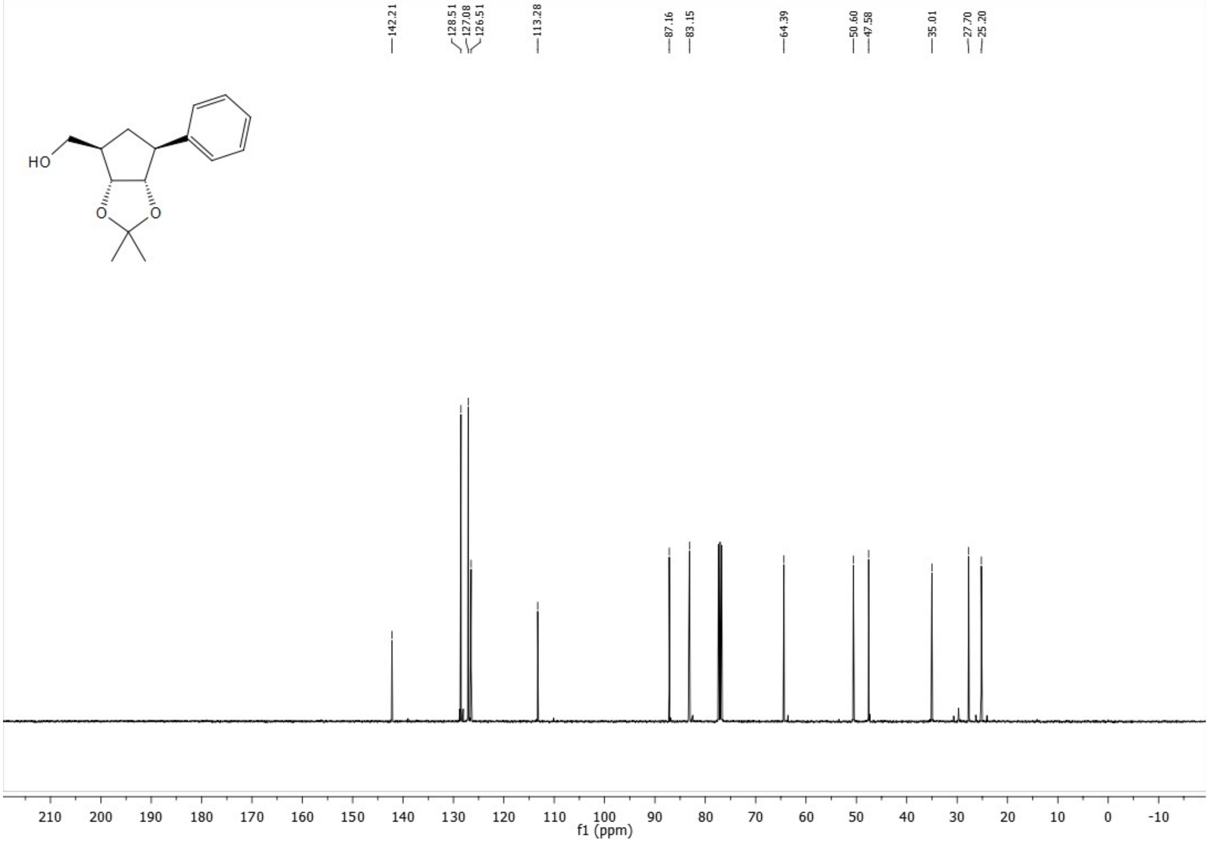

((3aR,4R,6S,6aS)-6-(4-fluorophenyl)-2,2-dimethyltetrahydro-4H-cyclopenta[d][1,3]dioxol-4-yl)methanol (10b)

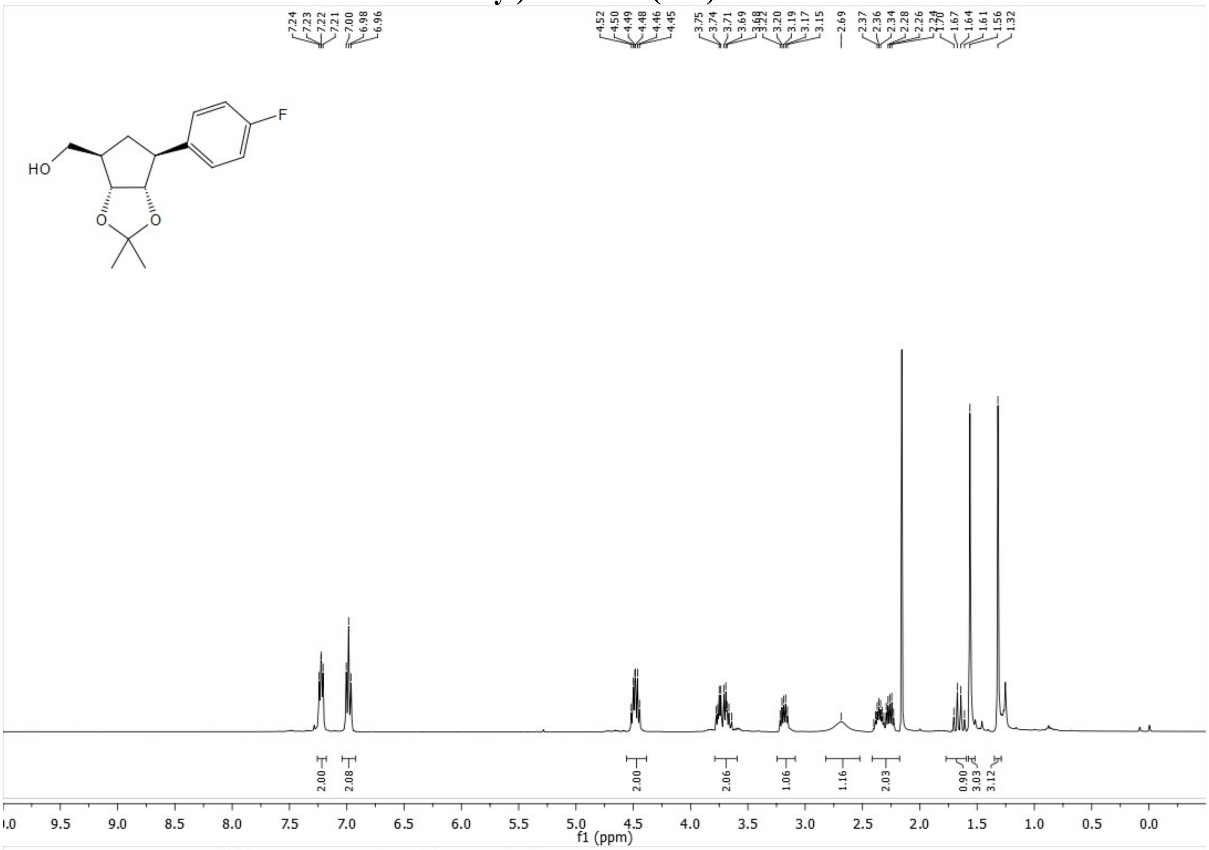

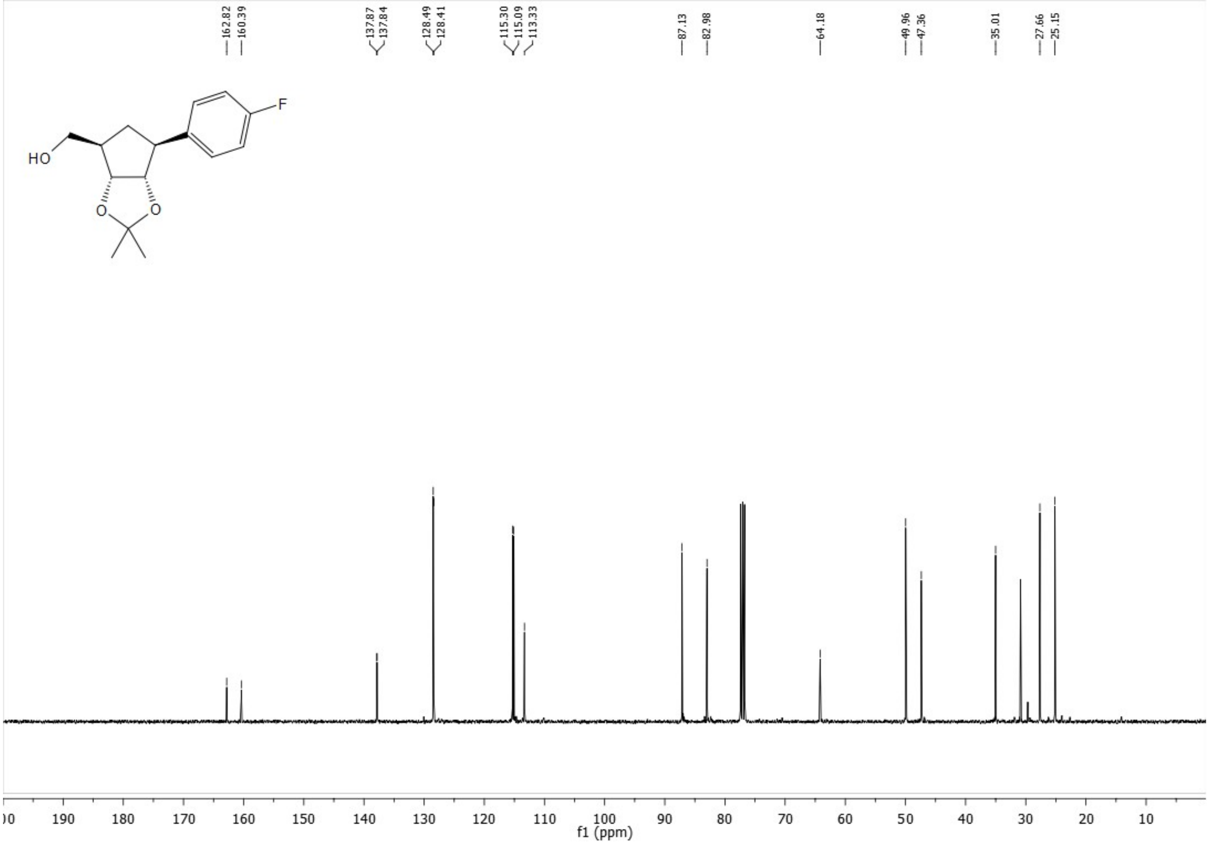

((3aR,4R,6S,6aS)-6-(4-methoxyphenyl)-2,2-dimethyltetrahydro-4H-cyclopenta[d][1,3]dioxol-4-yl)methanol (10c)

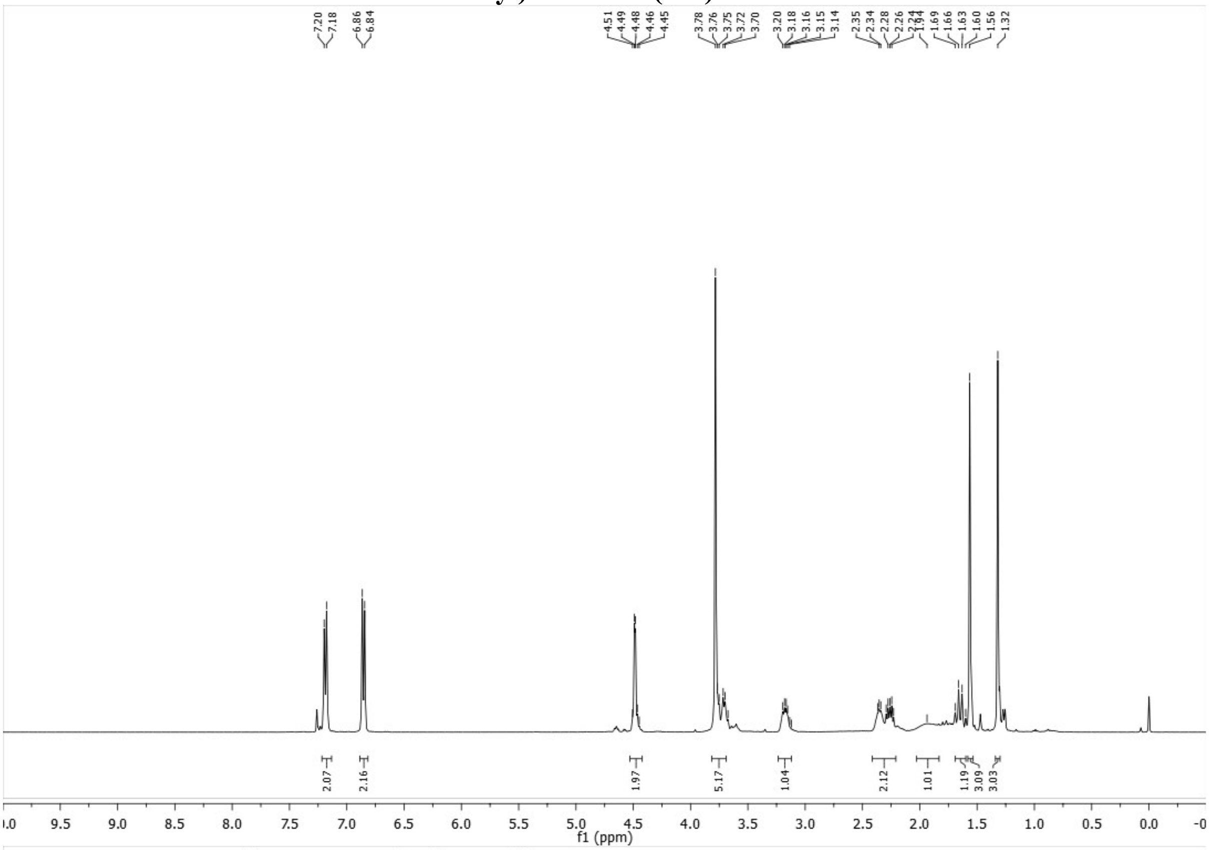

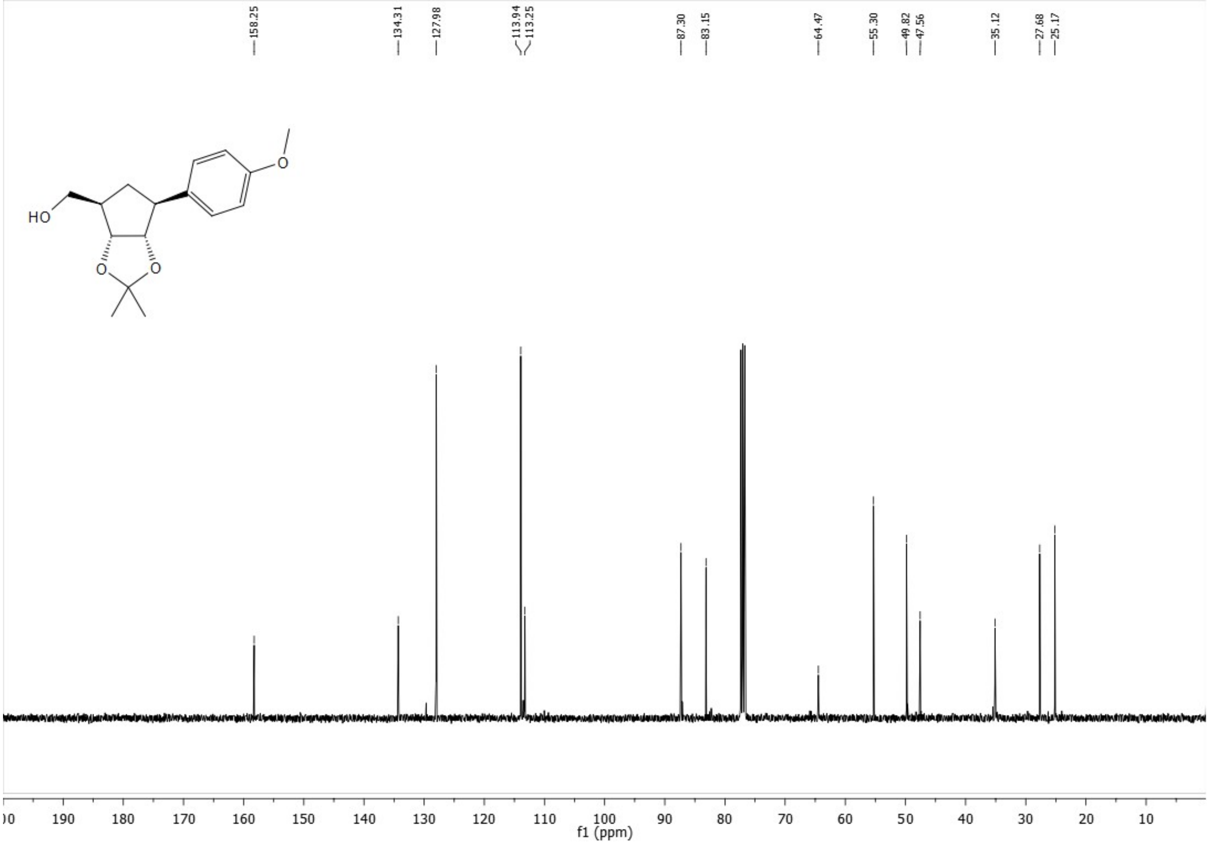

((3aR,4R,6S,6aS)-2,2-dimethyl-6-(4-(trifluoromethoxy)phenyl)tetrahydro-4H-cyclopenta[d][1,3]dioxol-4-yl)methanol (10d)

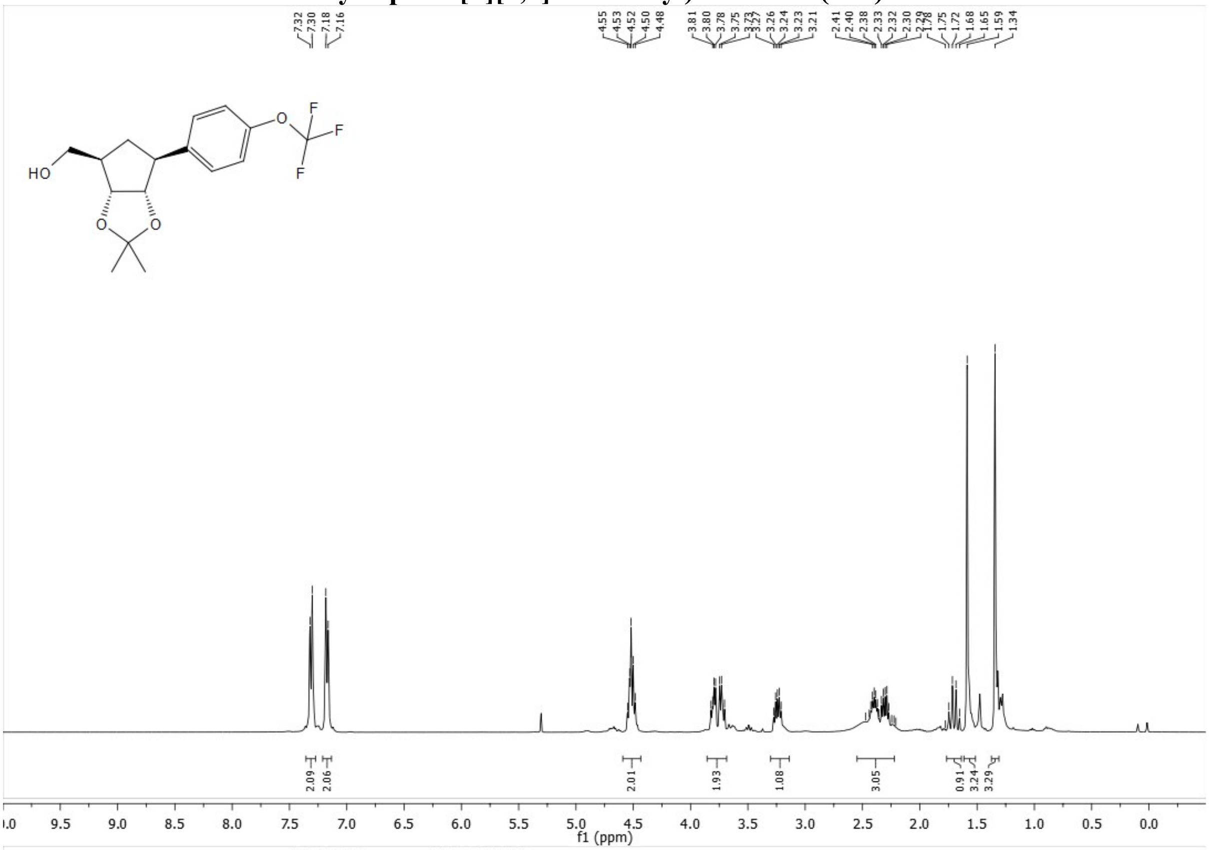

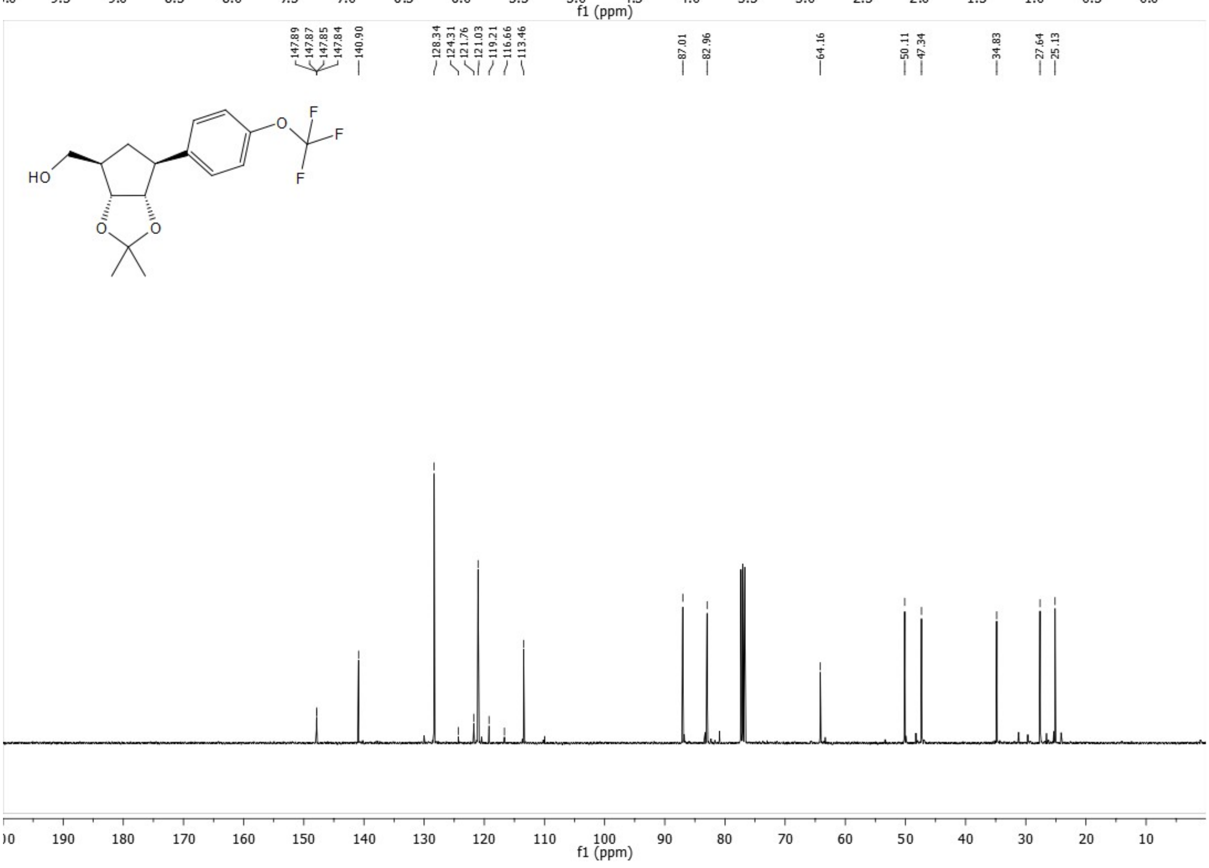

((3aR,4R,6S,6aS)-2,2-dimethyl-6-(2-methylpyridin-3-yl)tetrahydro-4H-cyclopenta[d][1,3]dioxol-4-yl)methanol (10e)

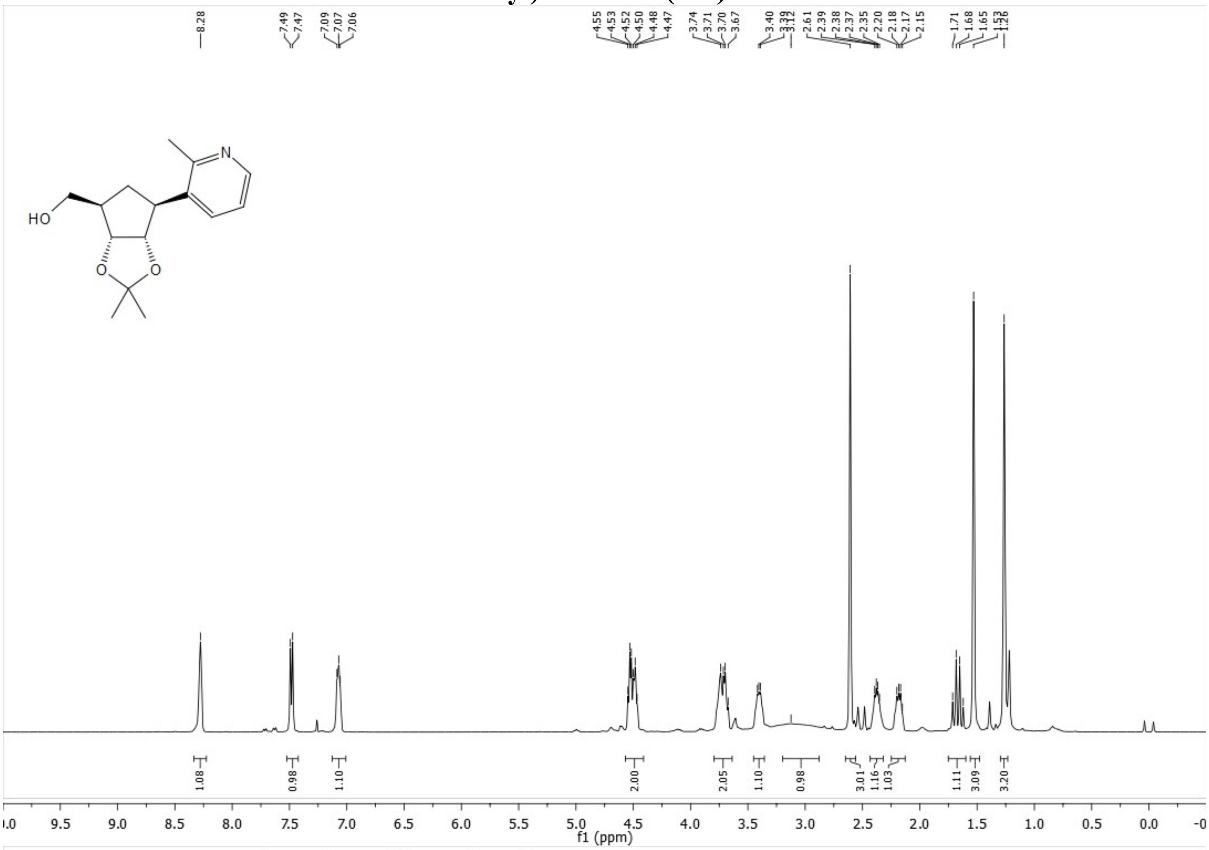

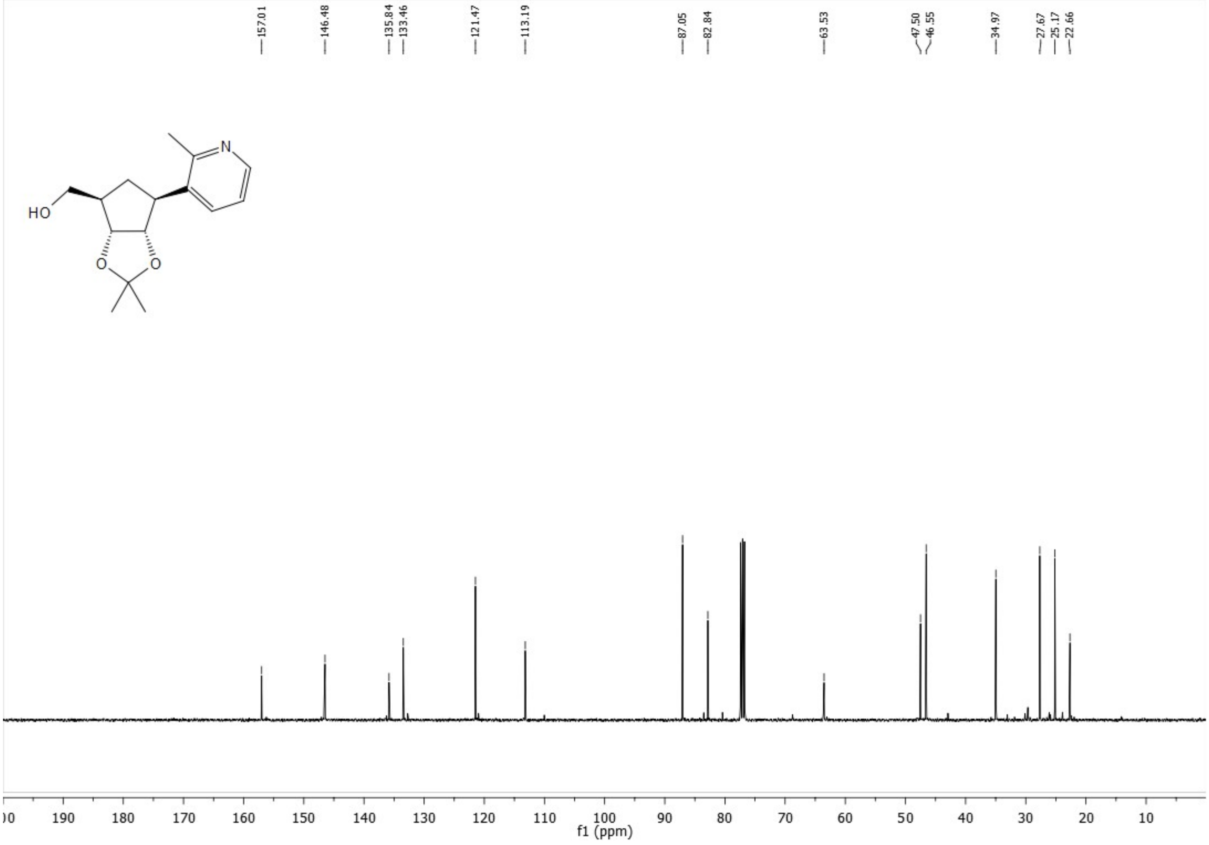

((3aR,4R,6S,6aS)-2,2-dimethyl-6-(naphthalen-2-yl)tetrahydro-4H-cyclopenta[d][1,3]dioxol-4-yl)methanol (10f)

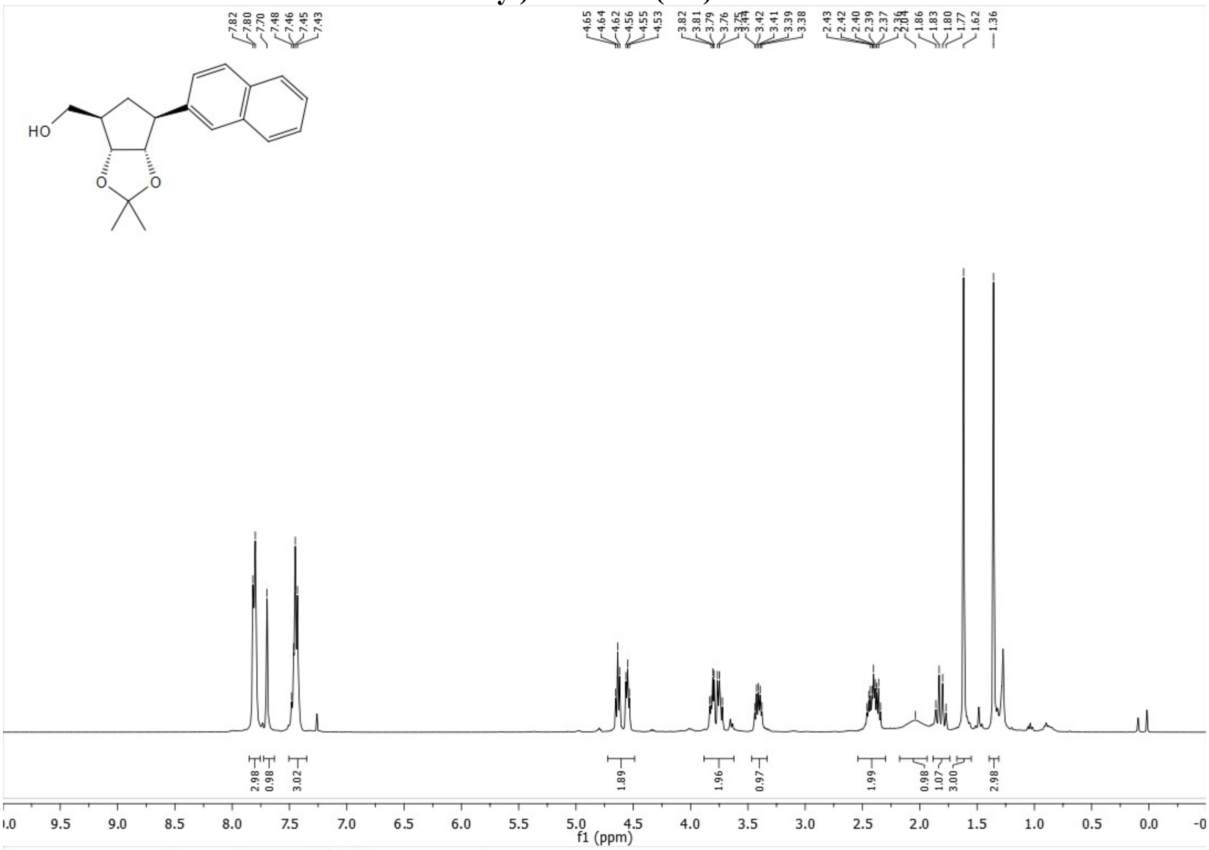

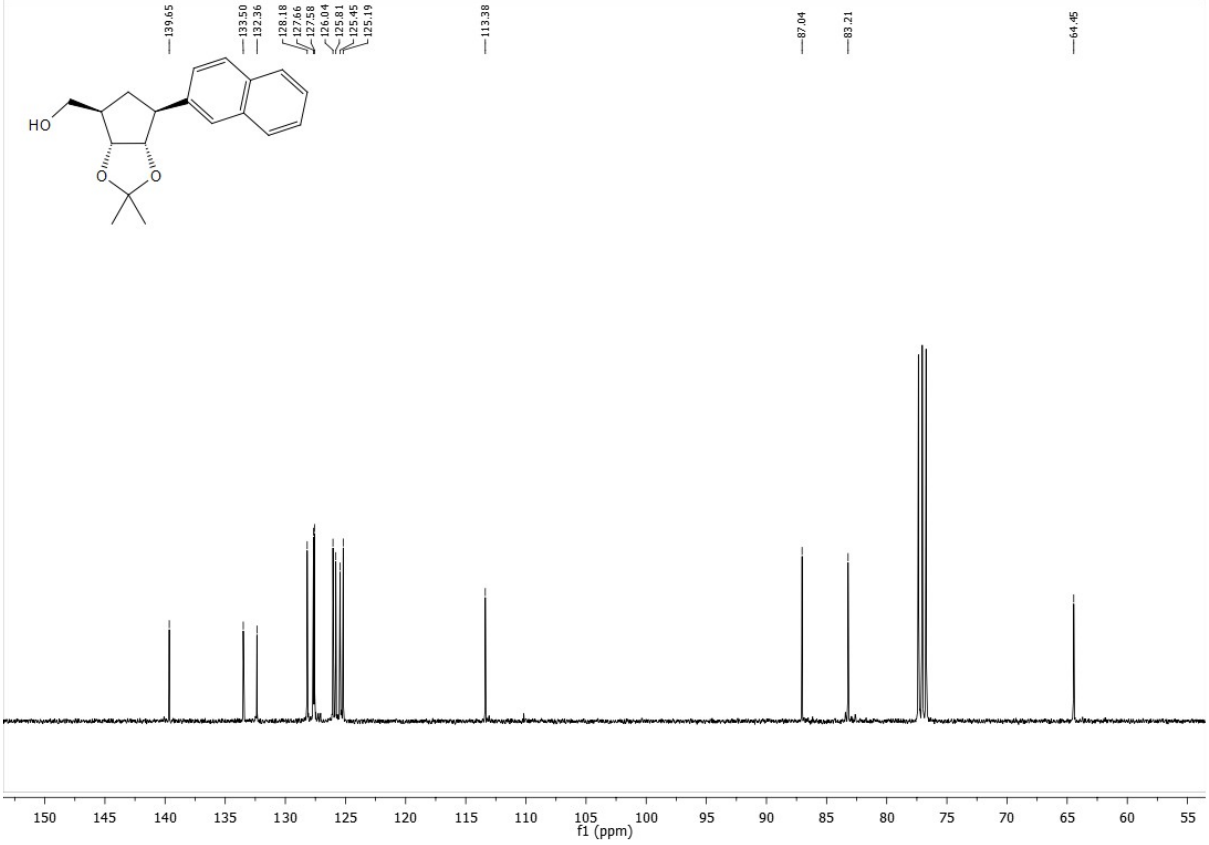

((3aR,4R,6S,6aS)-6-(3-methoxynaphthalen-2-yl)-2,2-dimethyltetrahydro-4H-cyclopenta[d][1,3]dioxol-4-yl)methanol (10g)

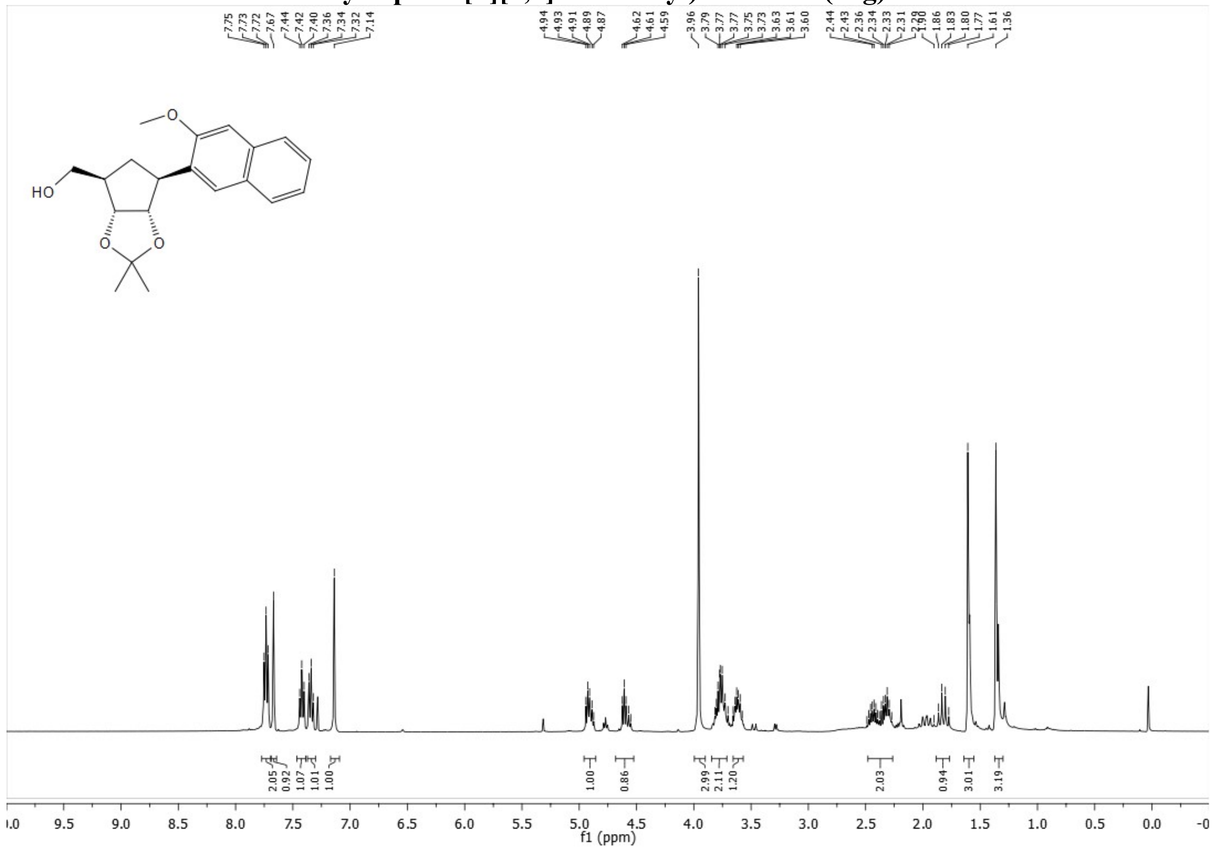

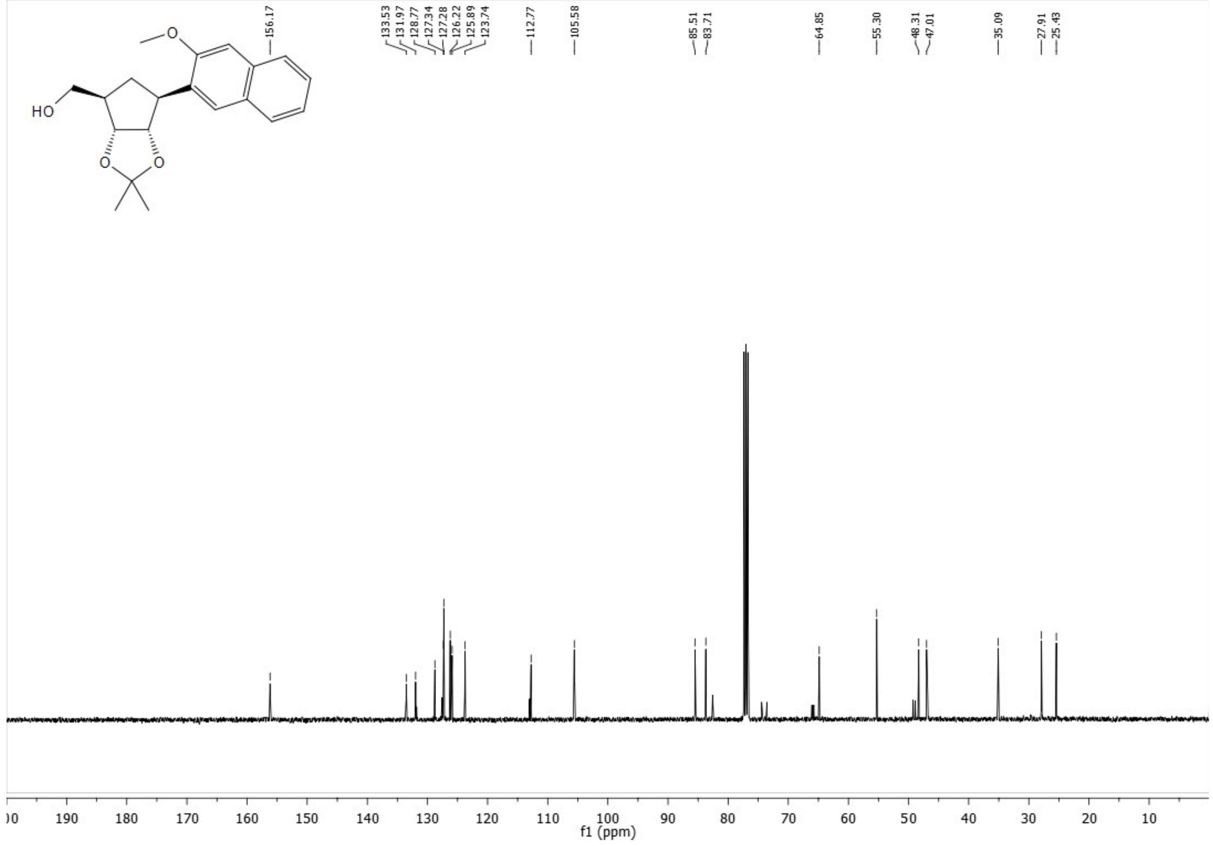

((3aR,4R,6S,6aS)-2,2-dimethyl-6-(naphthalen-1-yl)tetrahydro-4H-cyclopenta[d][1,3]dioxol-4-yl)methanol (10h)

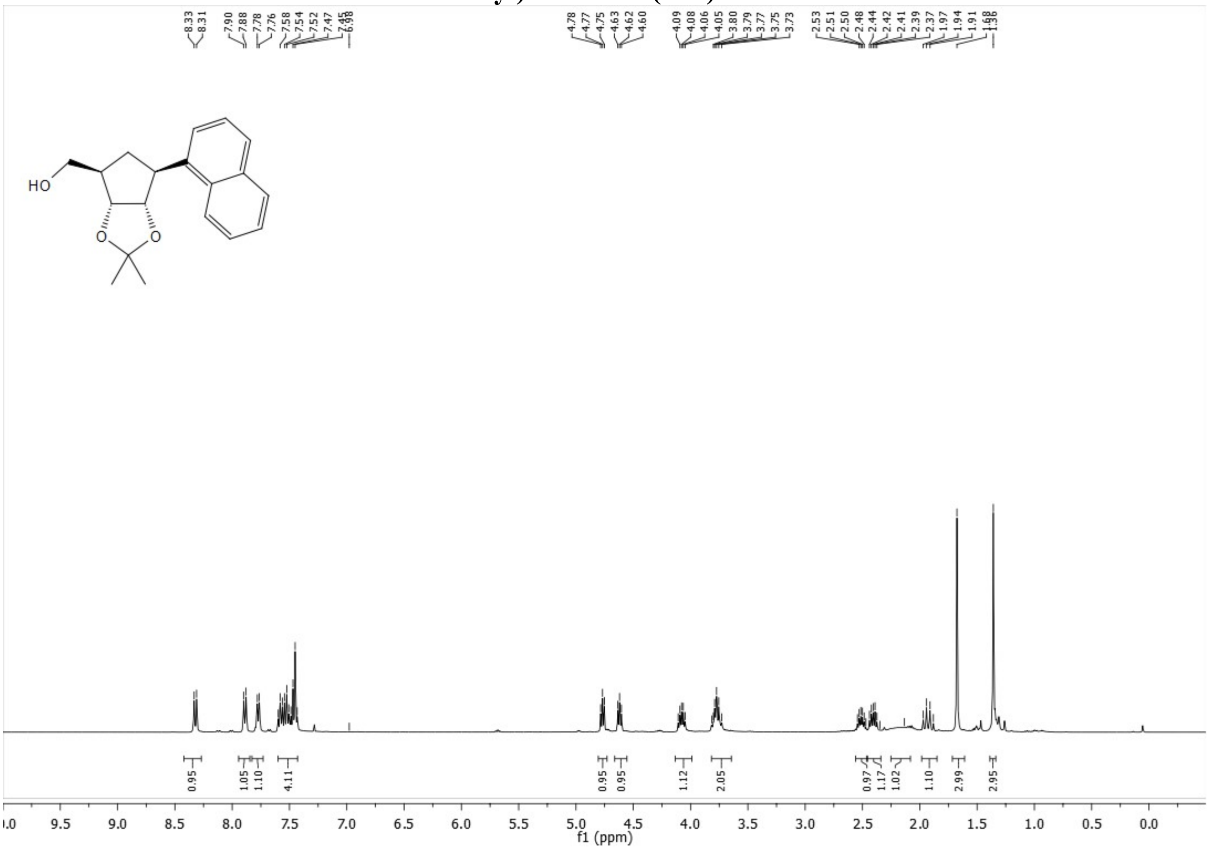

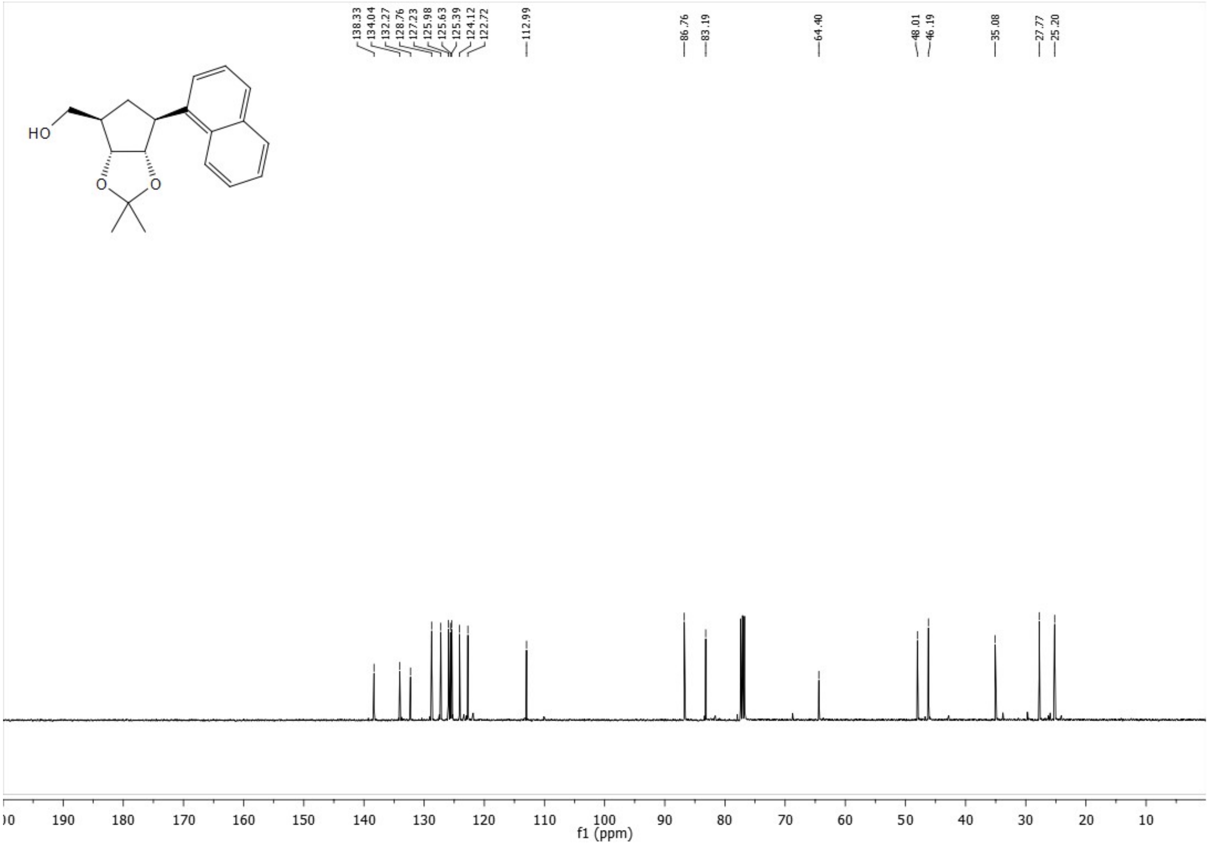

((3aR,4R,6S,6aS)-6-(2,4-difluoro-5-methylphenyl)-2,2-dimethyltetrahydro-4H-cyclopenta[d][1,3]dioxol-4-yl)methanol (10i)

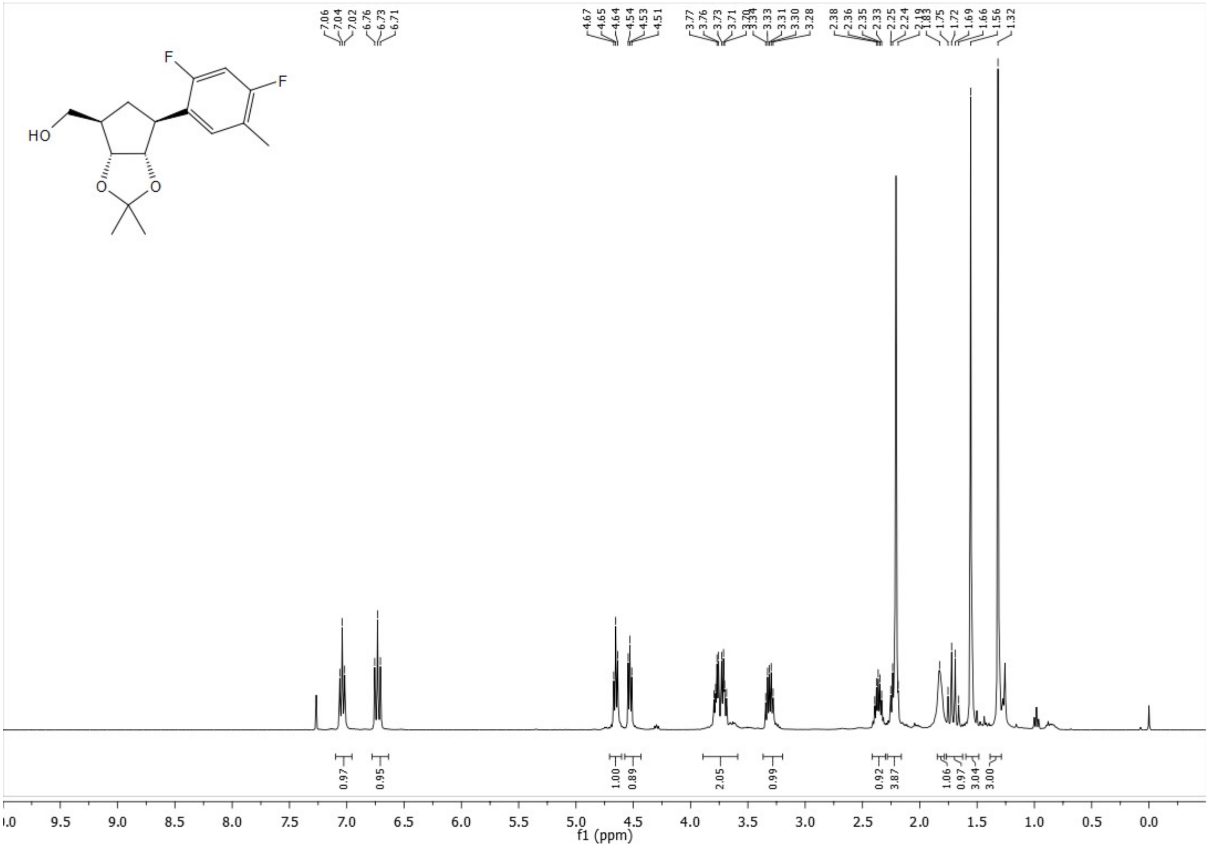

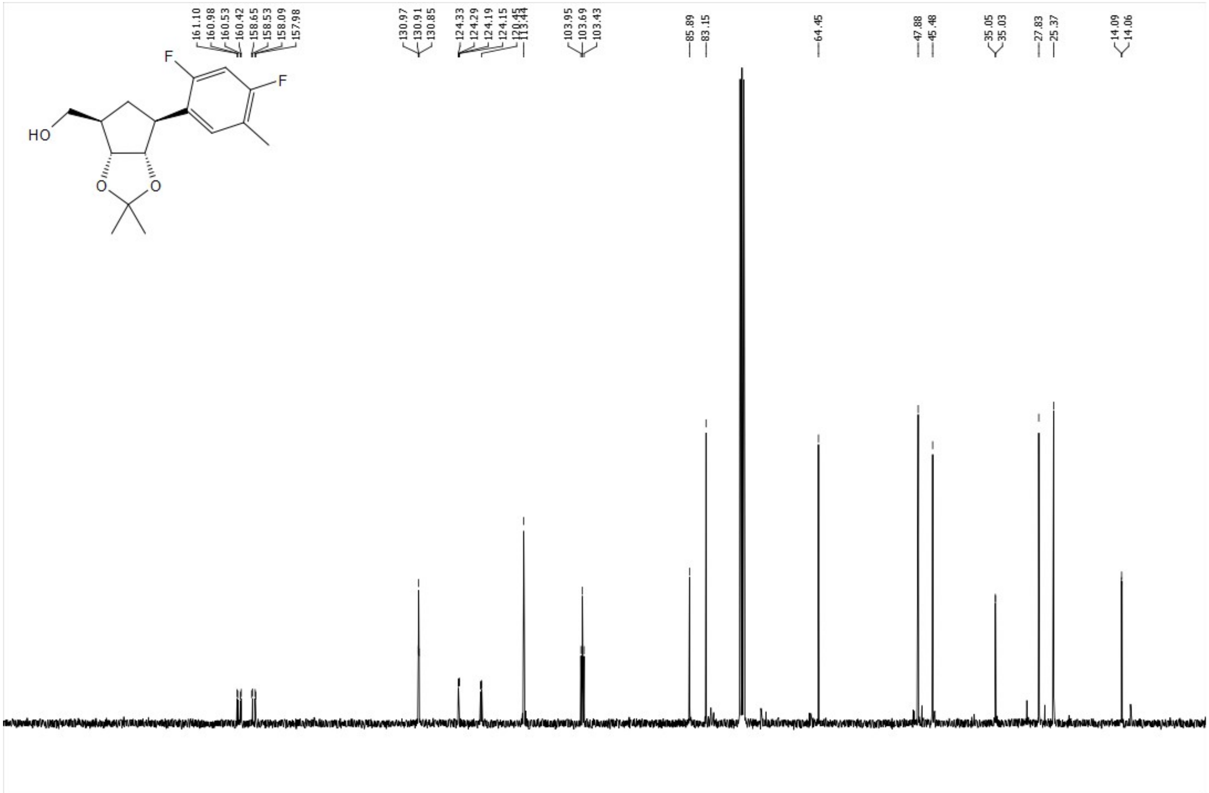

((3aR,4R,6S,6aS)-2,2-dimethyl-6-(pyridin-3-yl)tetrahydro-4H-cyclopenta[d][1,3]dioxol-4-yl)methanol (10j)

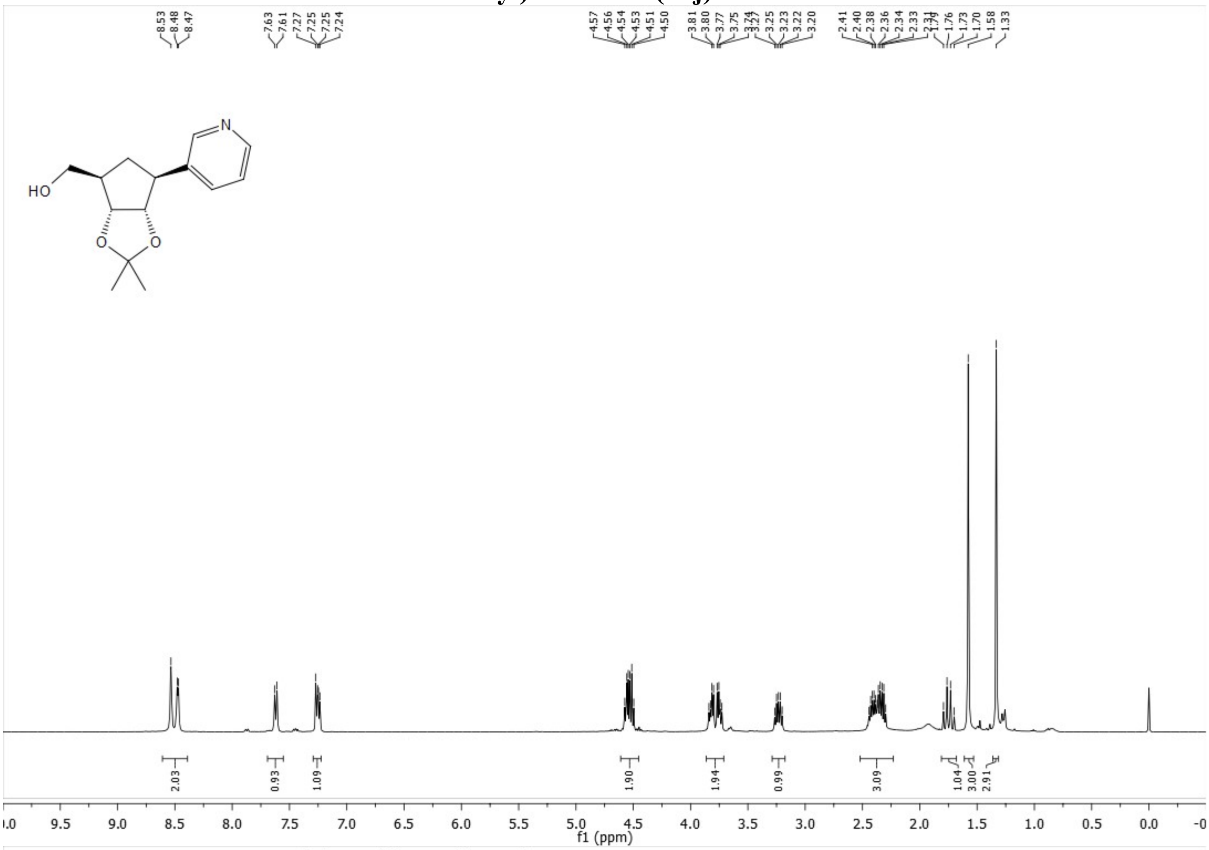

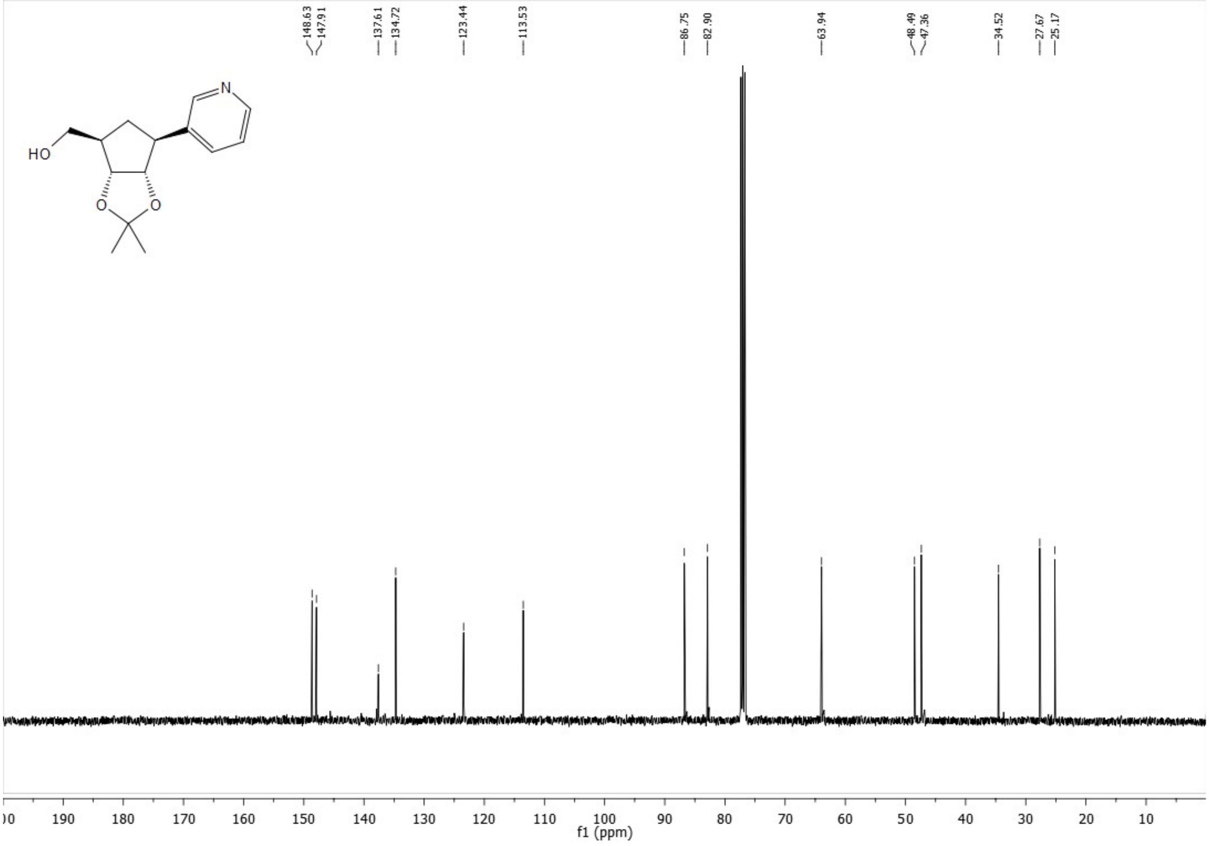

((3aR,4R,6S,6aS)-6-(6-fluoropyridin-3-yl)-2,2-dimethyltetrahydro-4H-cyclopenta[d][1,3]dioxol-4-yl)methanol (10k)

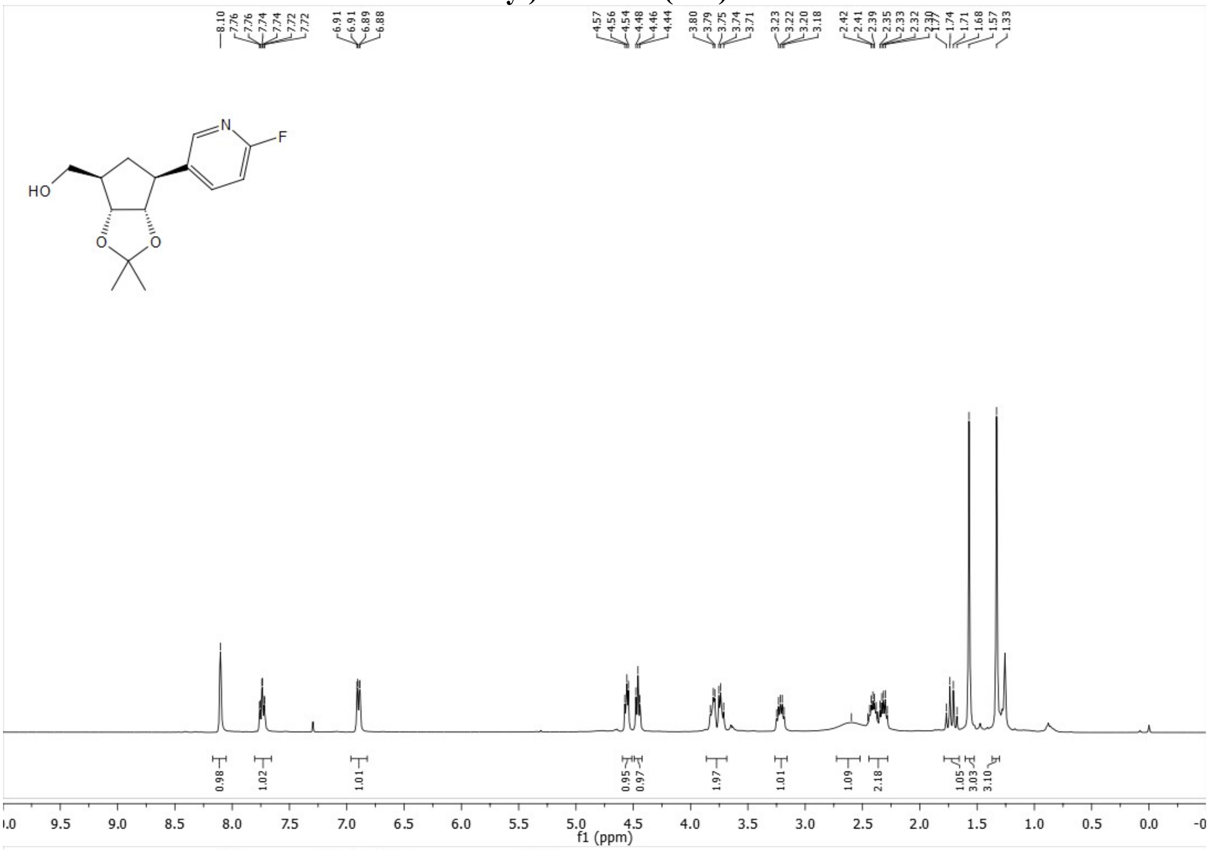

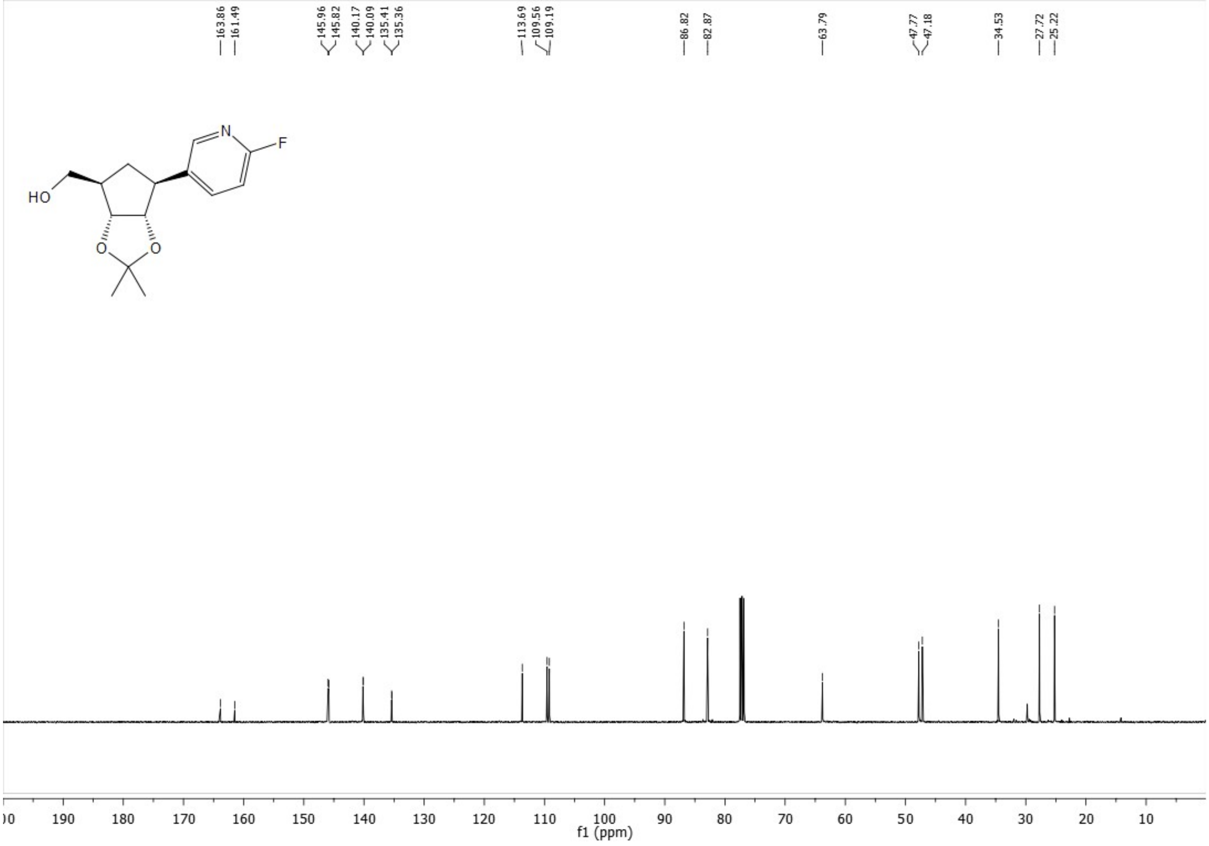

4-((3aS,4S,6R,6aR)-6-(hydroxymethyl)-2,2-dimethyltetrahydro-4H-cyclopenta[d][1,3]dioxol-4-yl)benzonitrile (10l)

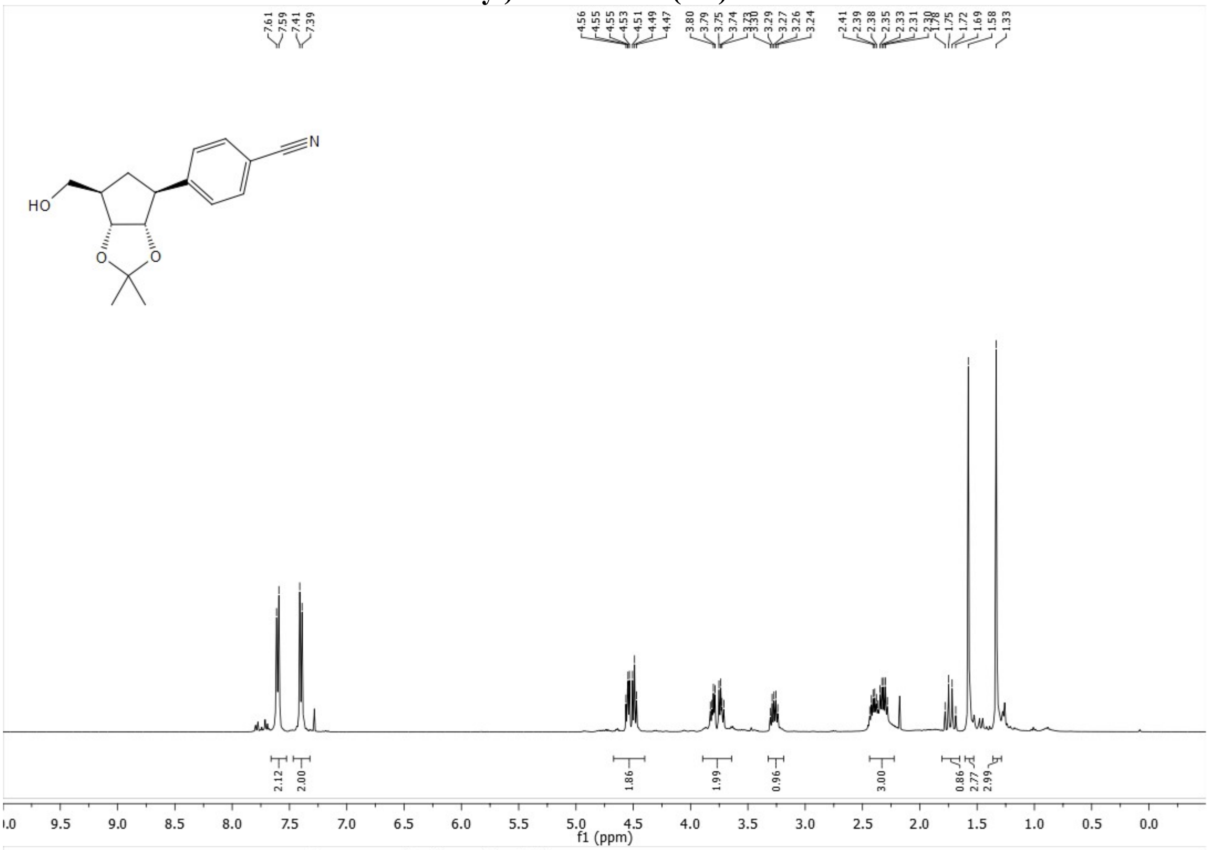

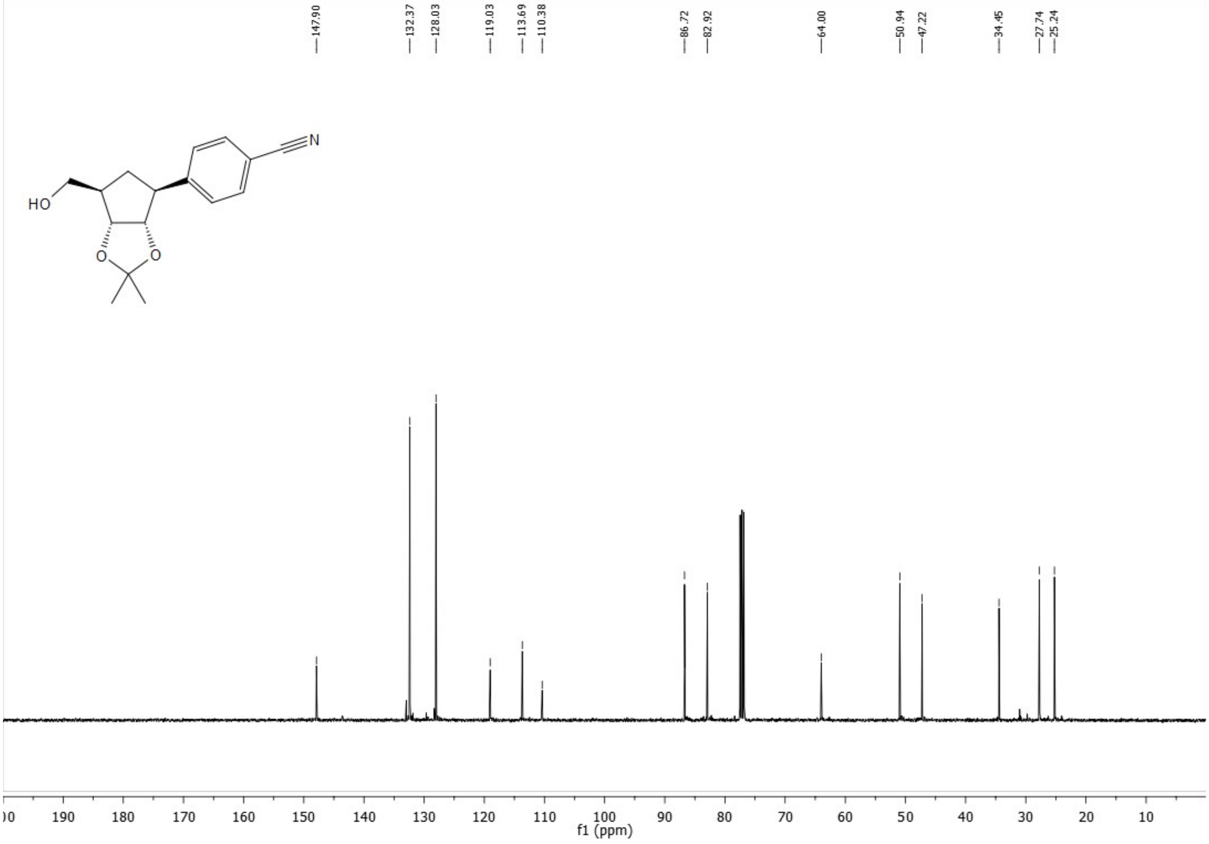

1S,2R,3R,5S)-3-(hydroxymethyl)-5-phenylcyclopentane-1,2-diol (11a)

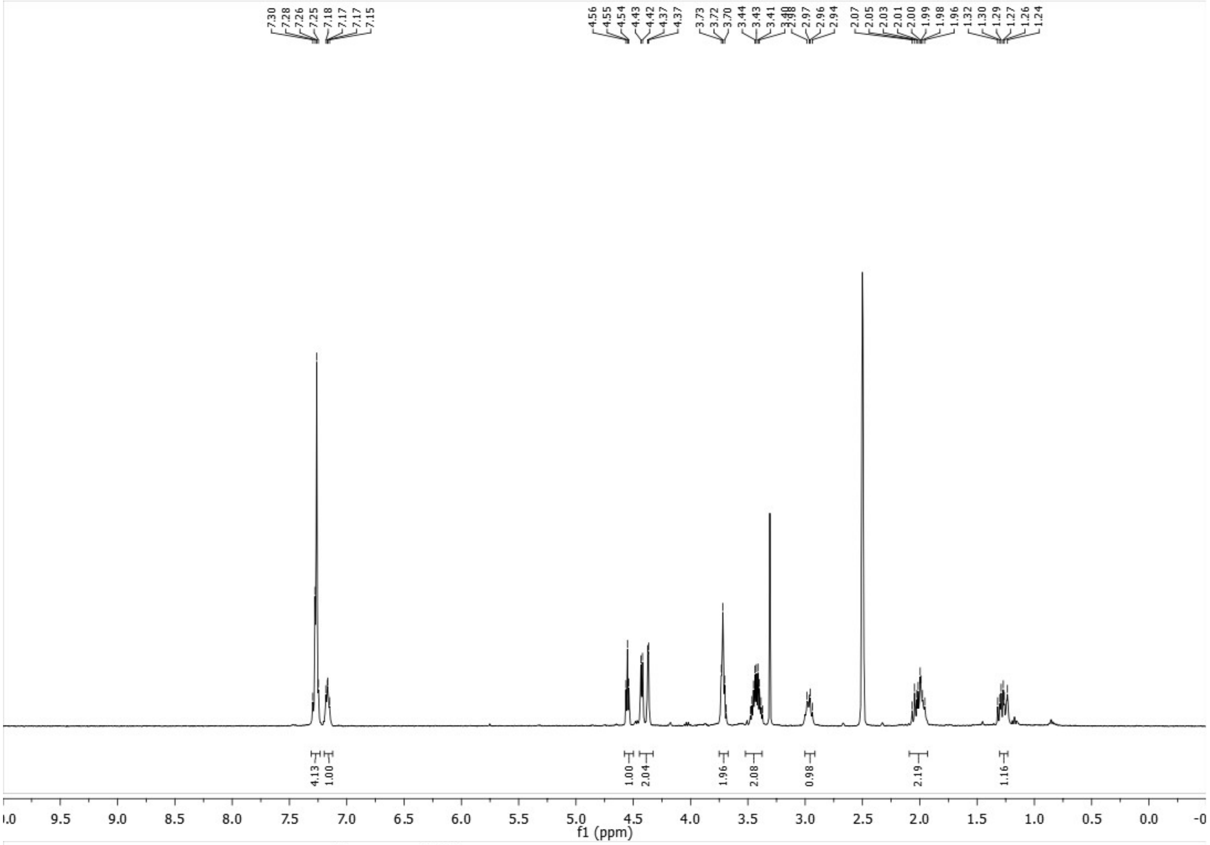

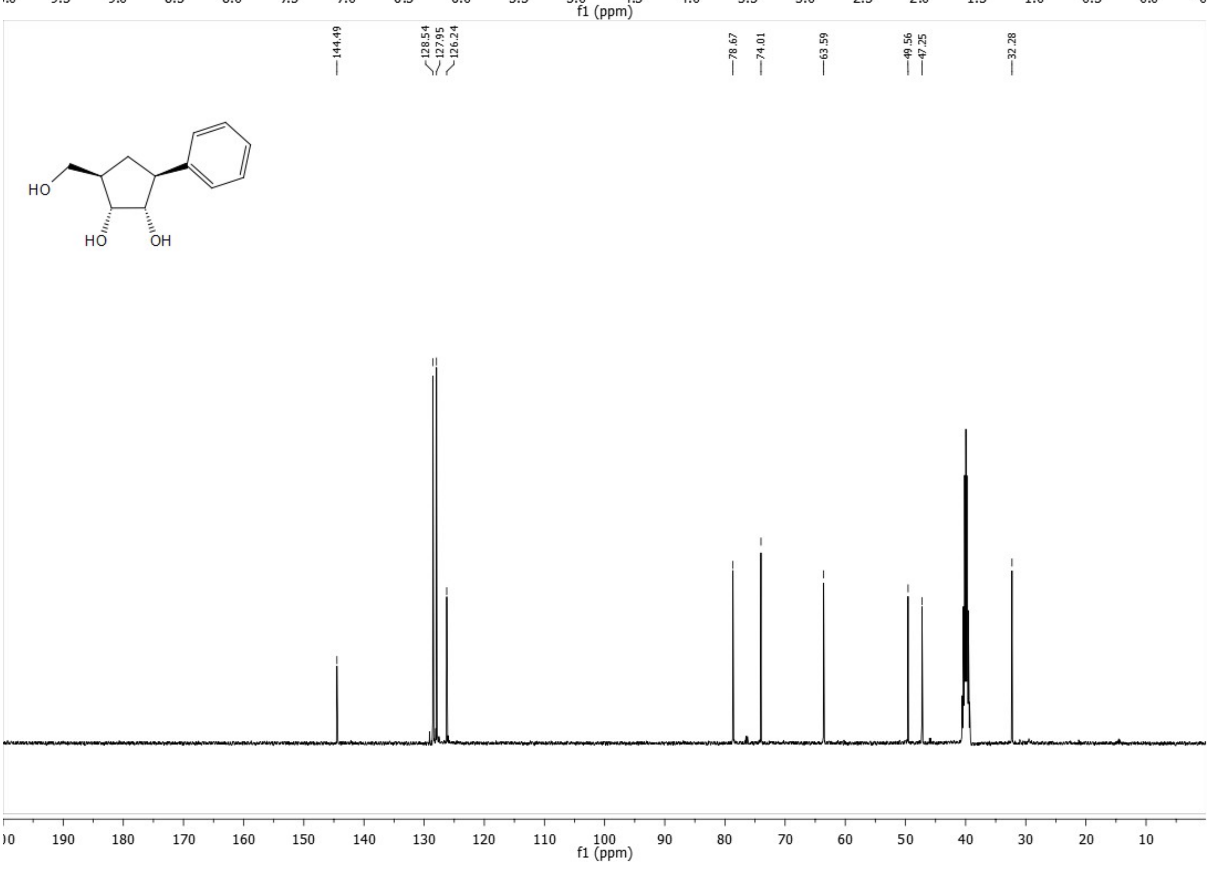

(1R,2S,3S,5R)-3-(4-fluorophenyl)-5-(hydroxymethyl)cyclopentane-1,2-diol (11b)

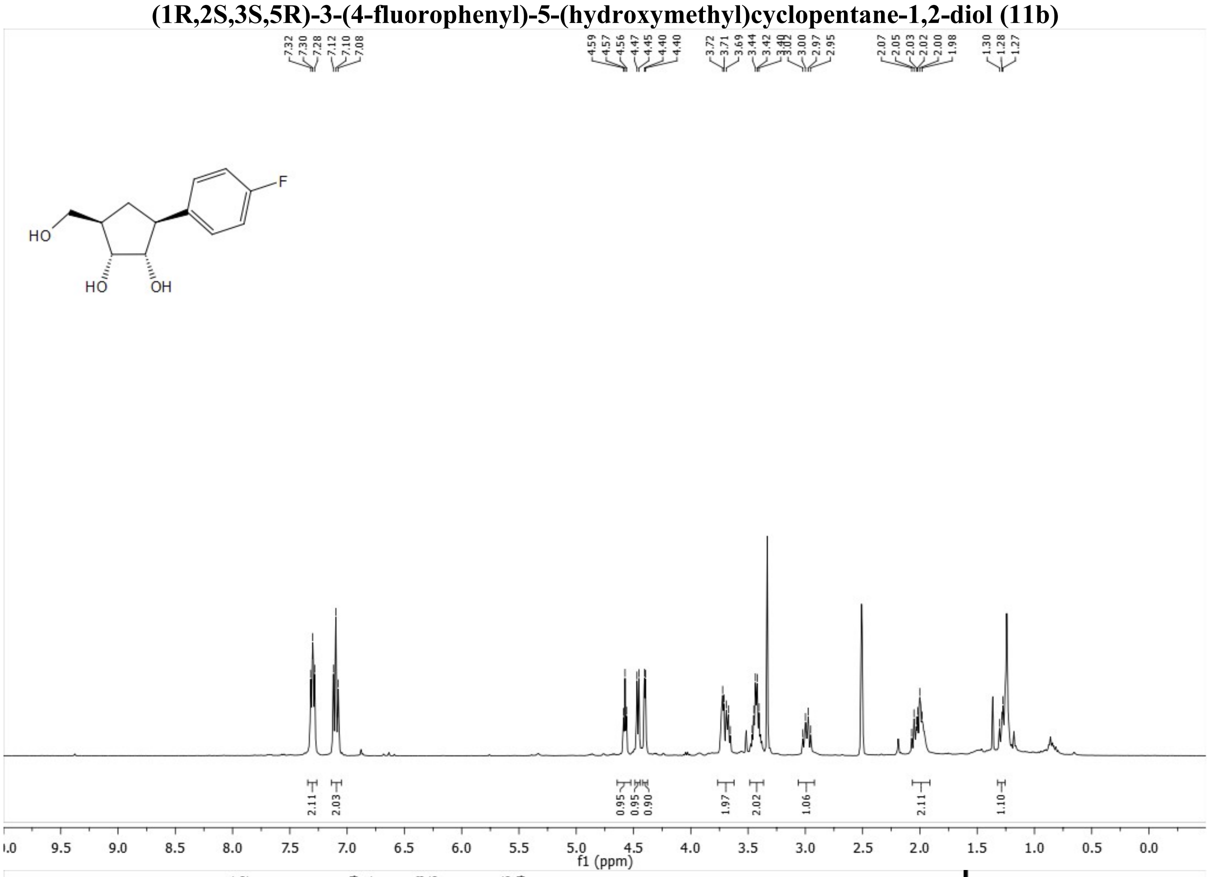

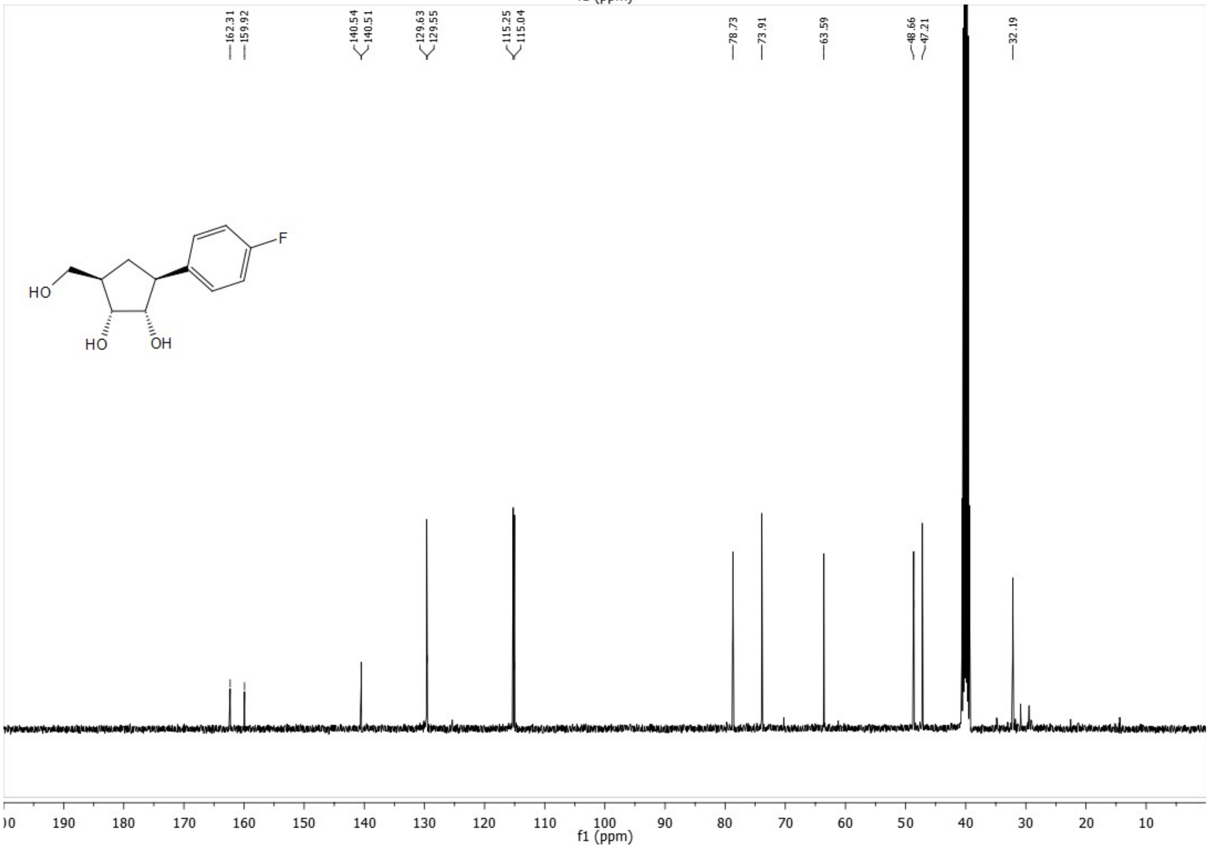

(1S,2R,3R,5S)-3-(hydroxymethyl)-5-(4-methoxyphenyl)cyclopentane-1,2-diol (11c)

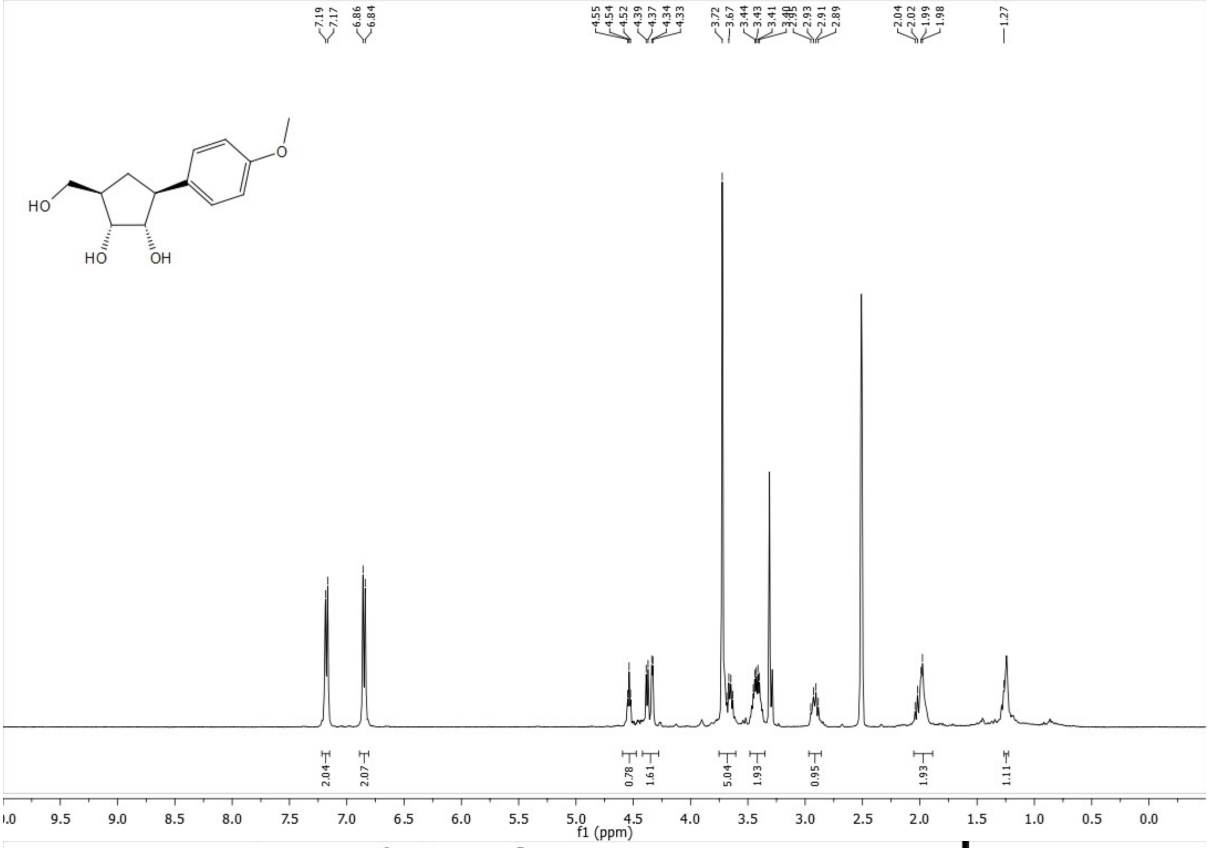

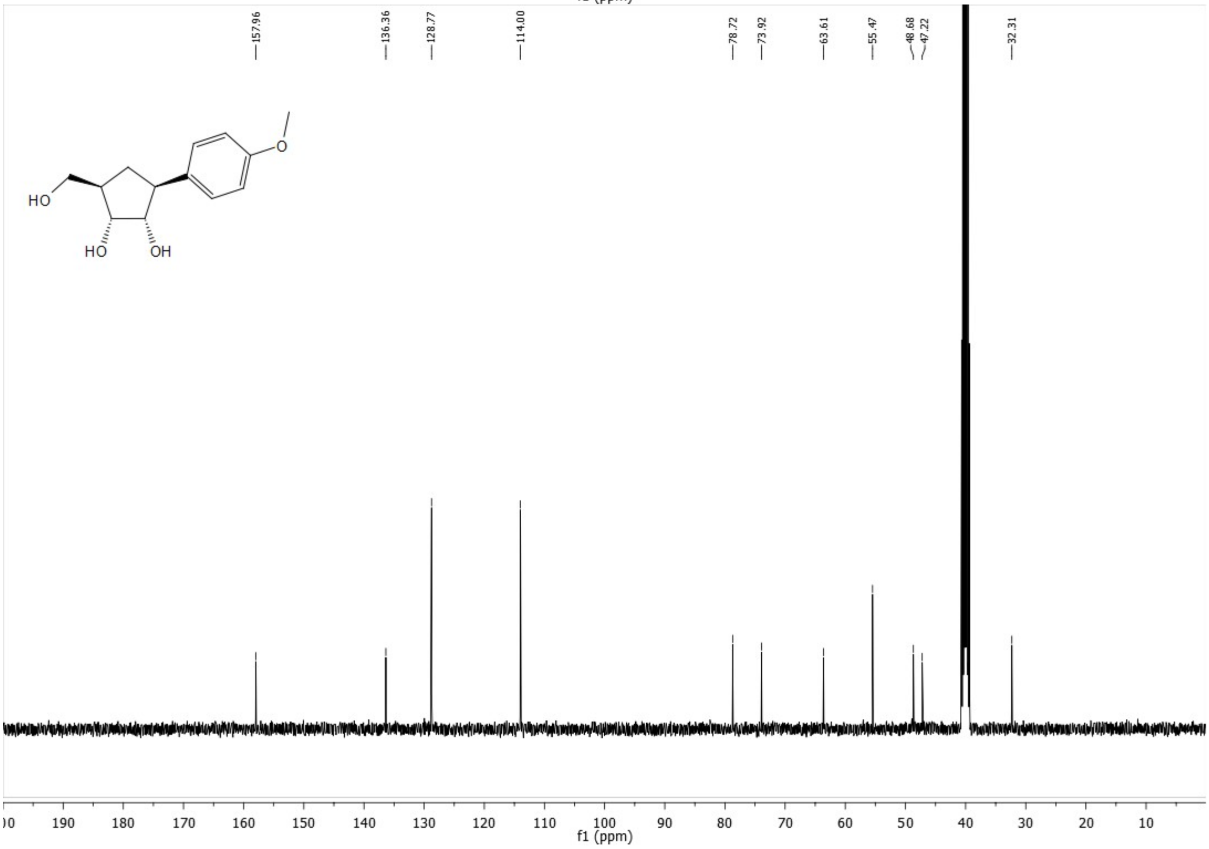

(1S,2R,3R,5S)-3-(hydroxymethyl)-5-(4-(trifluoromethoxy)phenyl)cyclopentane-1,2-diol (11d)

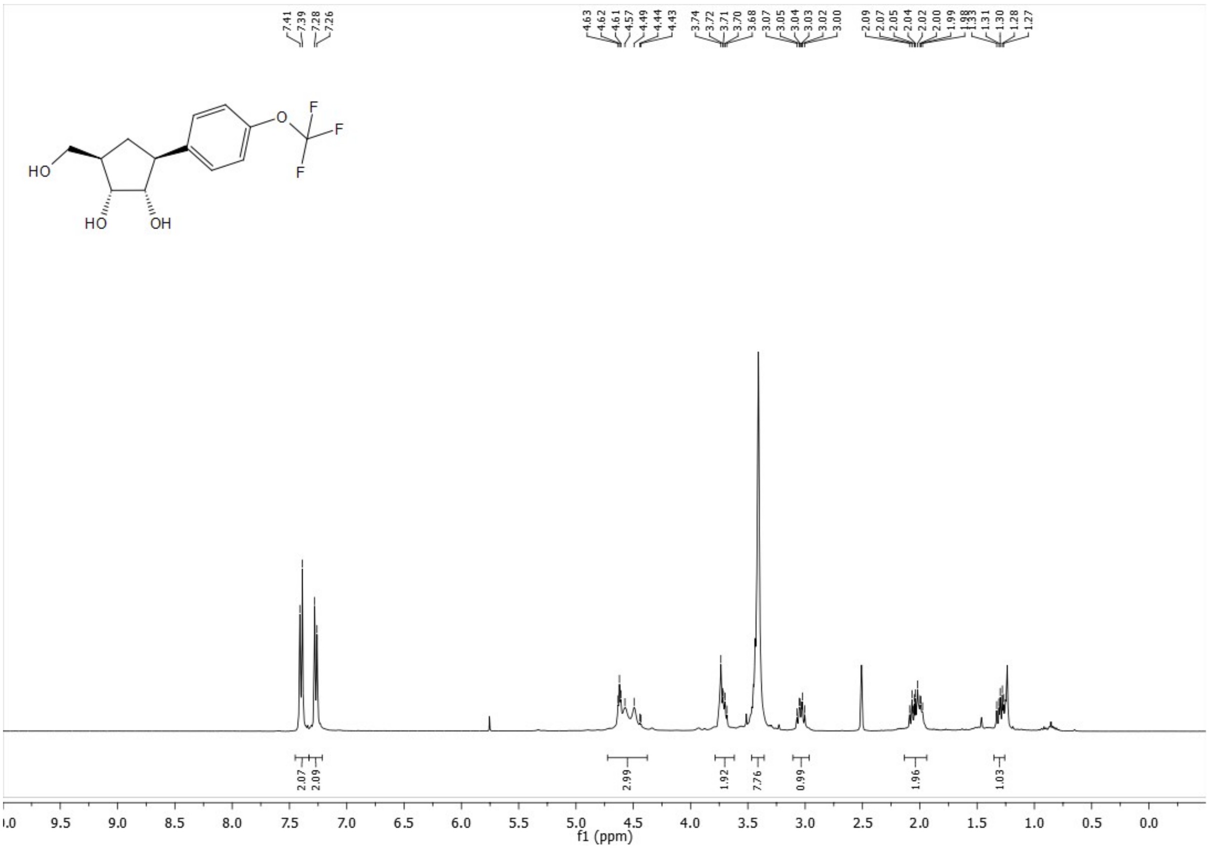

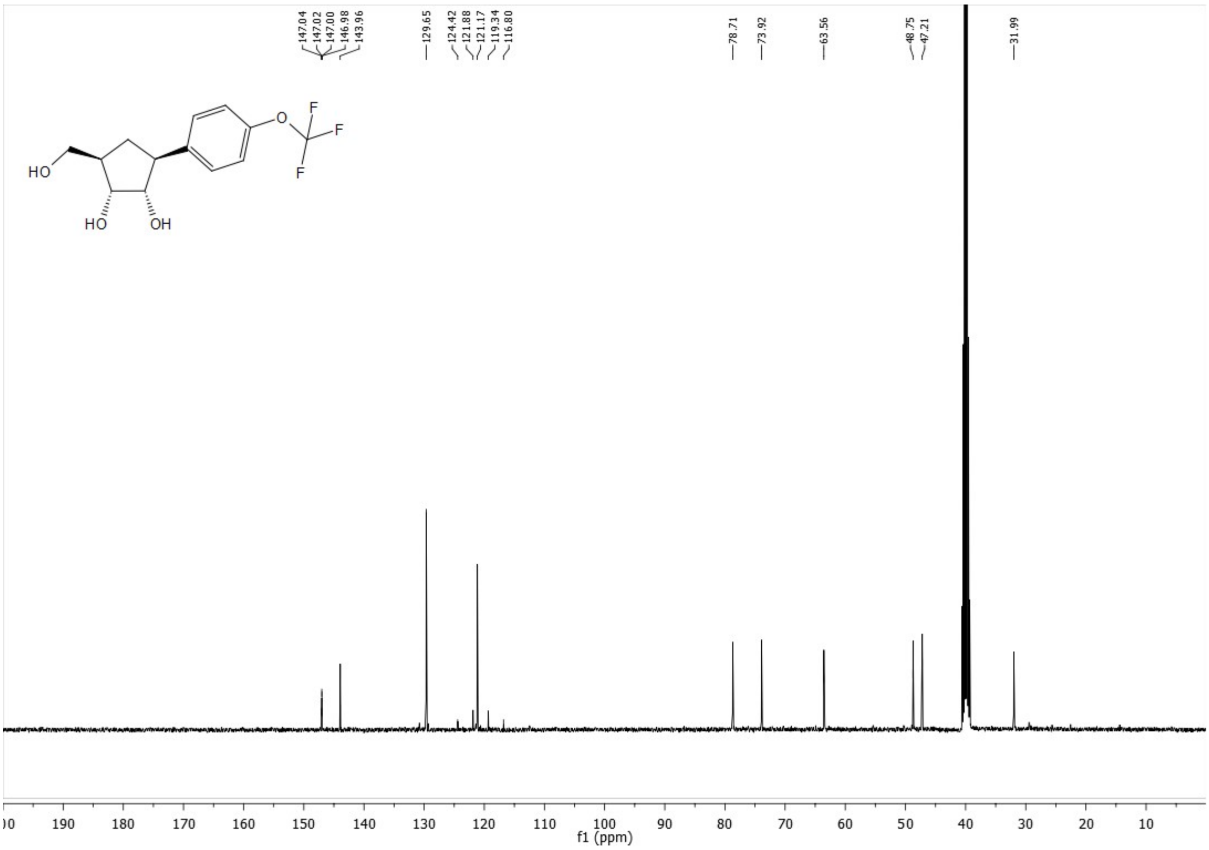

(1S,2R,3R,5S)-3-(hydroxymethyl)-5-(2-methylpyridin-3-yl)cyclopentane-1,2-diol (11e)

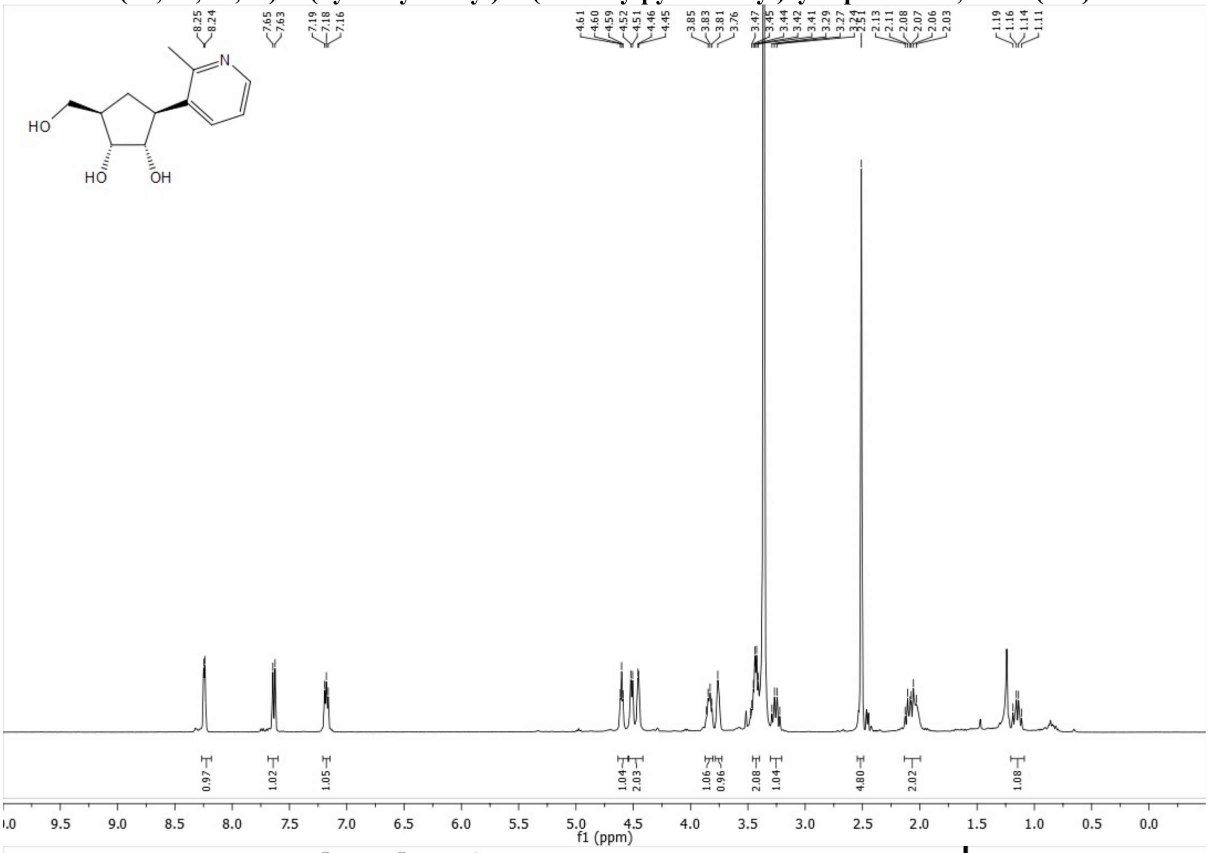

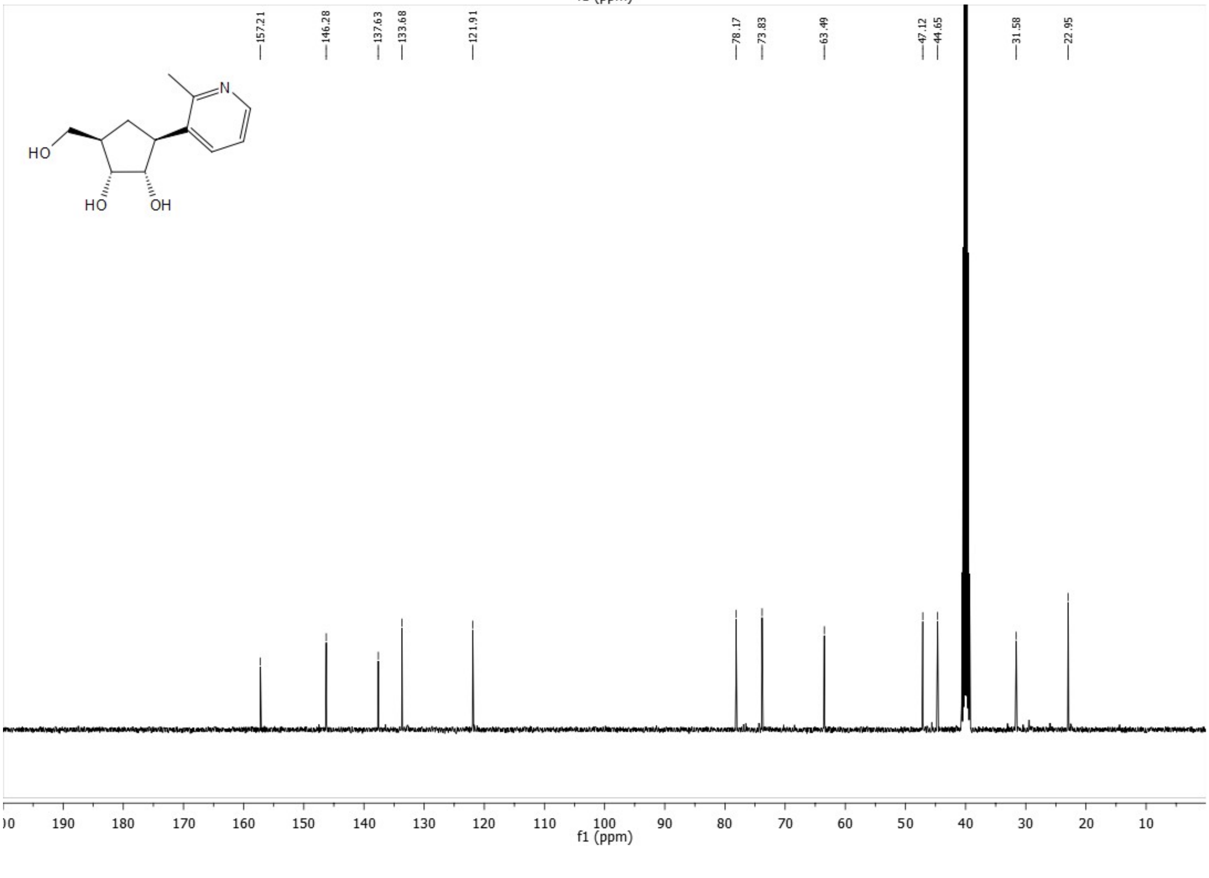

(1S,2R,3R,5S)-3-(hydroxymethyl)-5-(naphthalen-2-yl)cyclopentane-1,2-diol (11f)

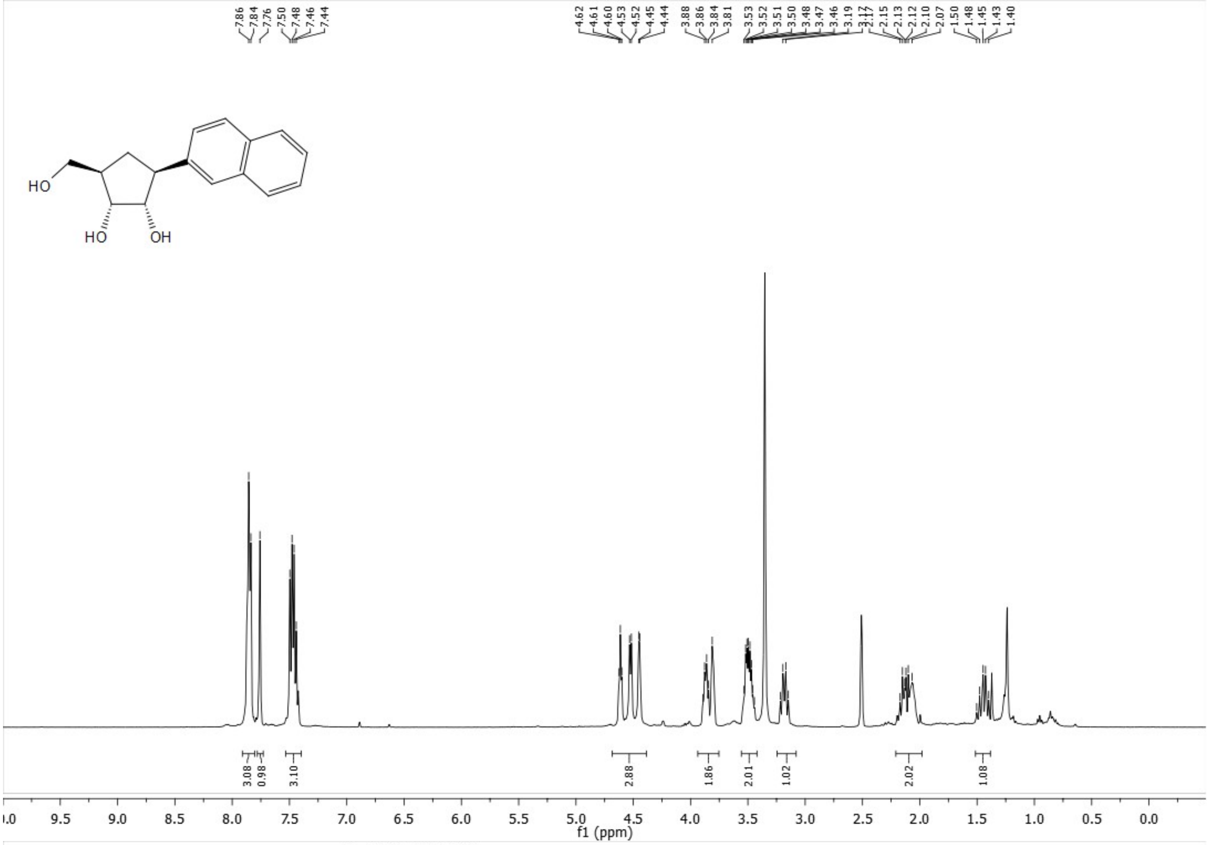

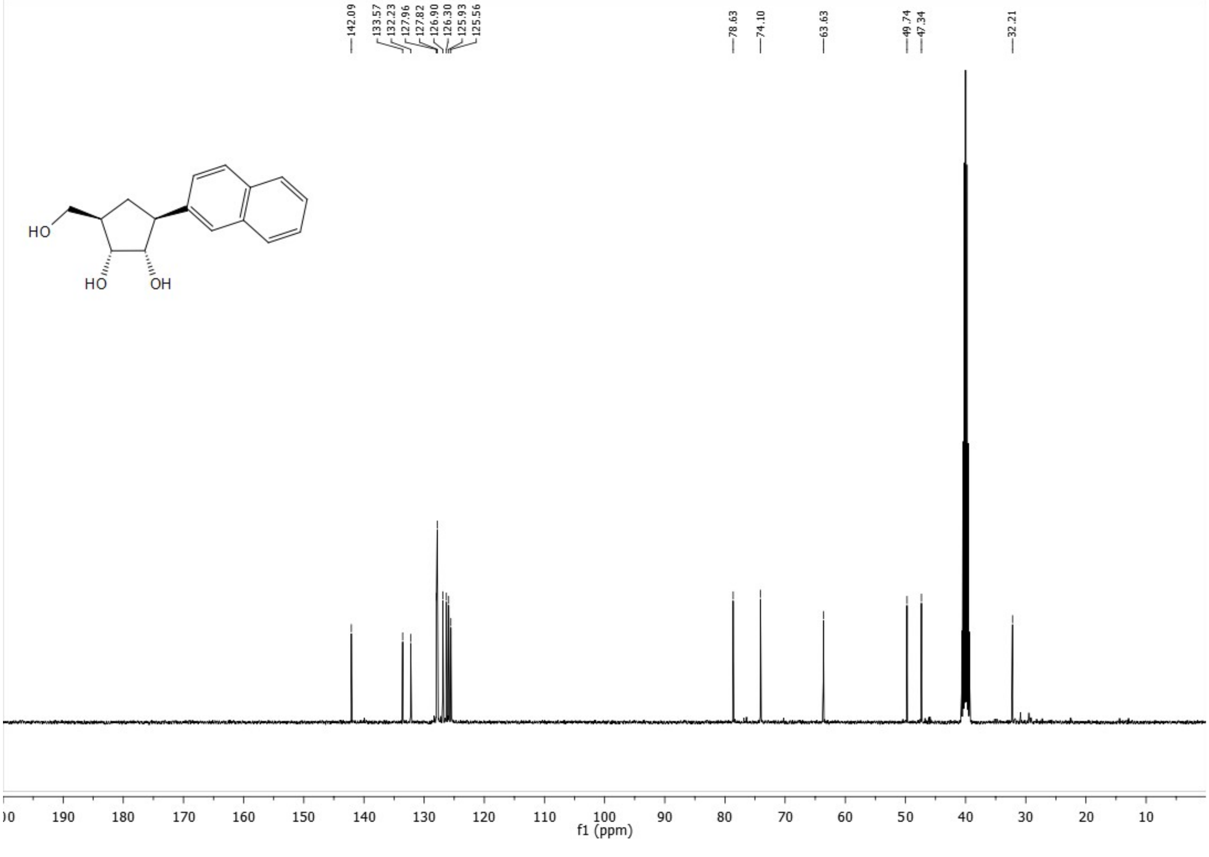

(1S,2R,3R,5S)-3-(hydroxymethyl)-5-(3-methoxynaphthalen-2-yl)cyclopentane-1,2-diol (11g)

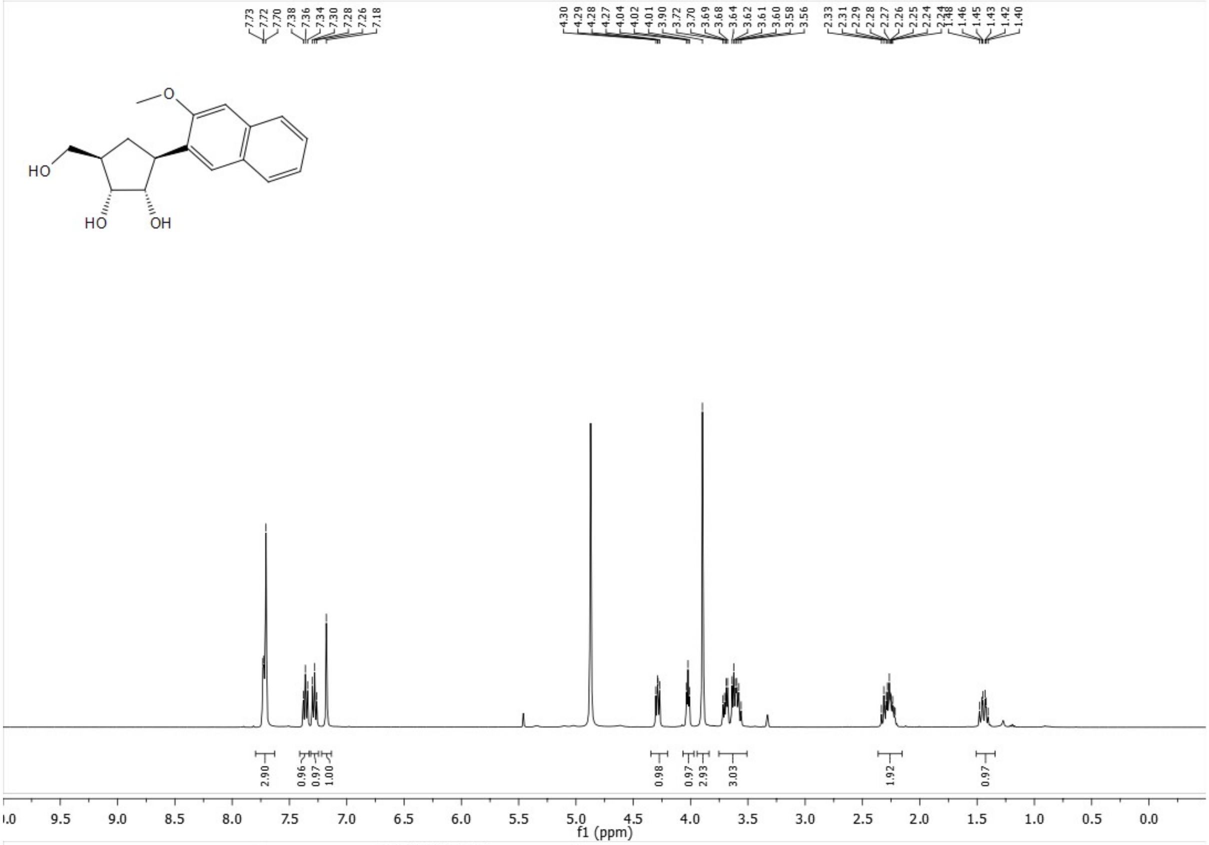

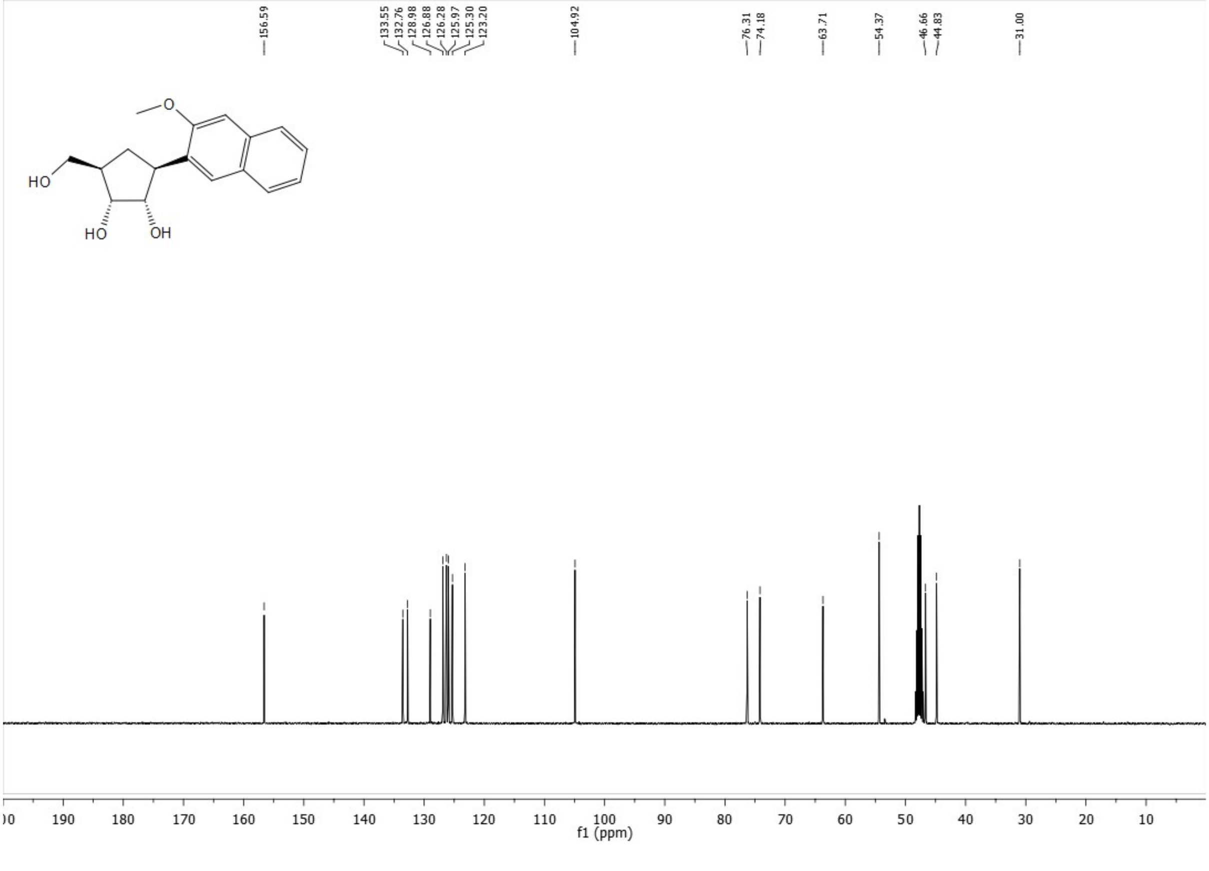

(1S,2R,3R,5S)-3-(hydroxymethyl)-5-(naphthalen-1-yl)cyclopentane-1,2-diol (11h)

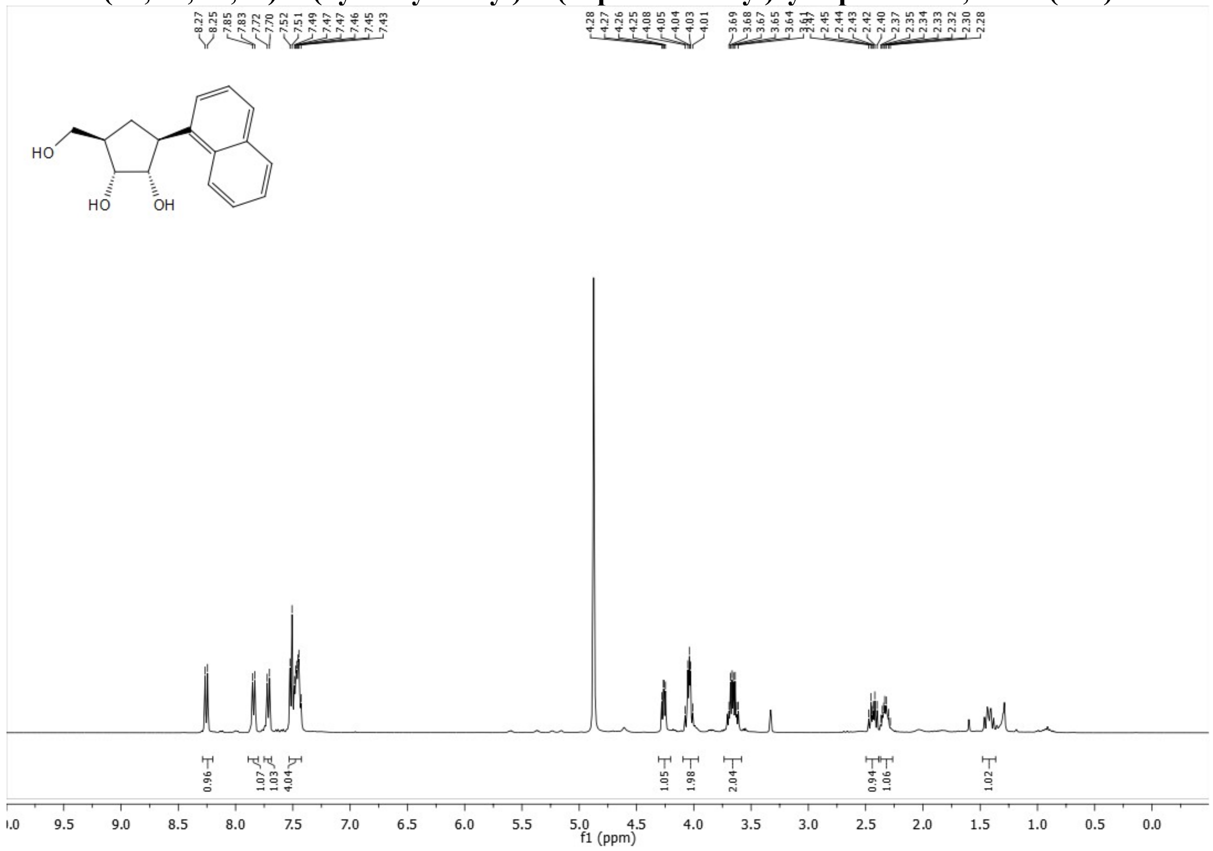

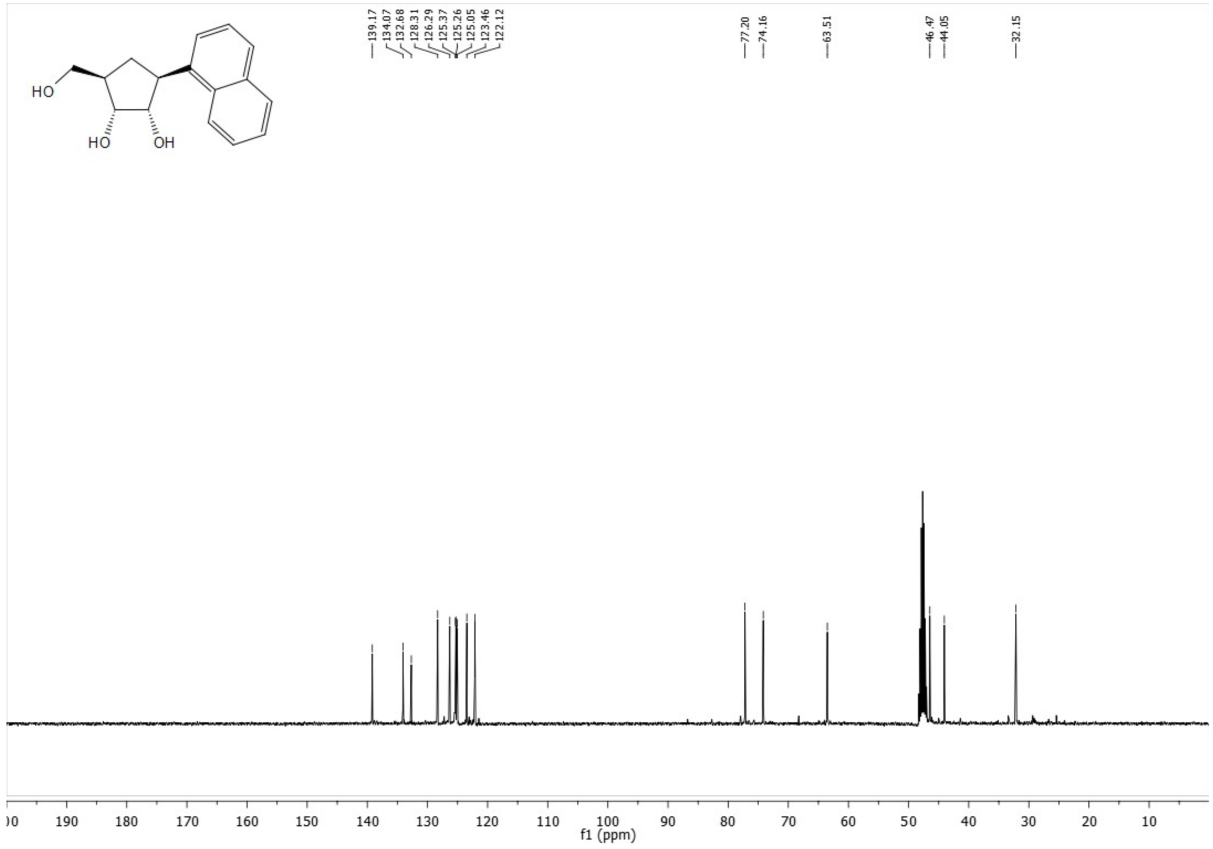

(1R,2S,3S,5R)-3-(2,4-difluoro-5-methylphenyl)-5-(hydroxymethyl)cyclopentane-1,2-diol (11i)

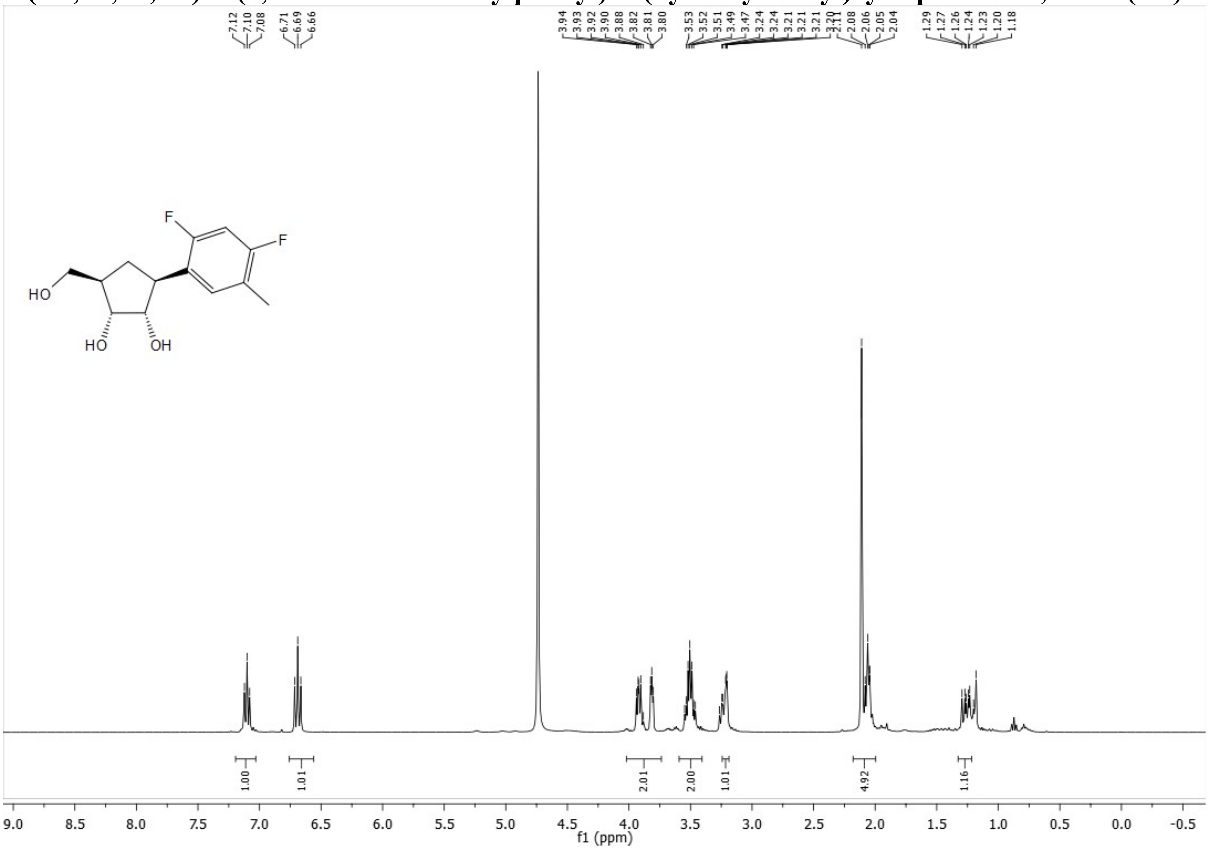

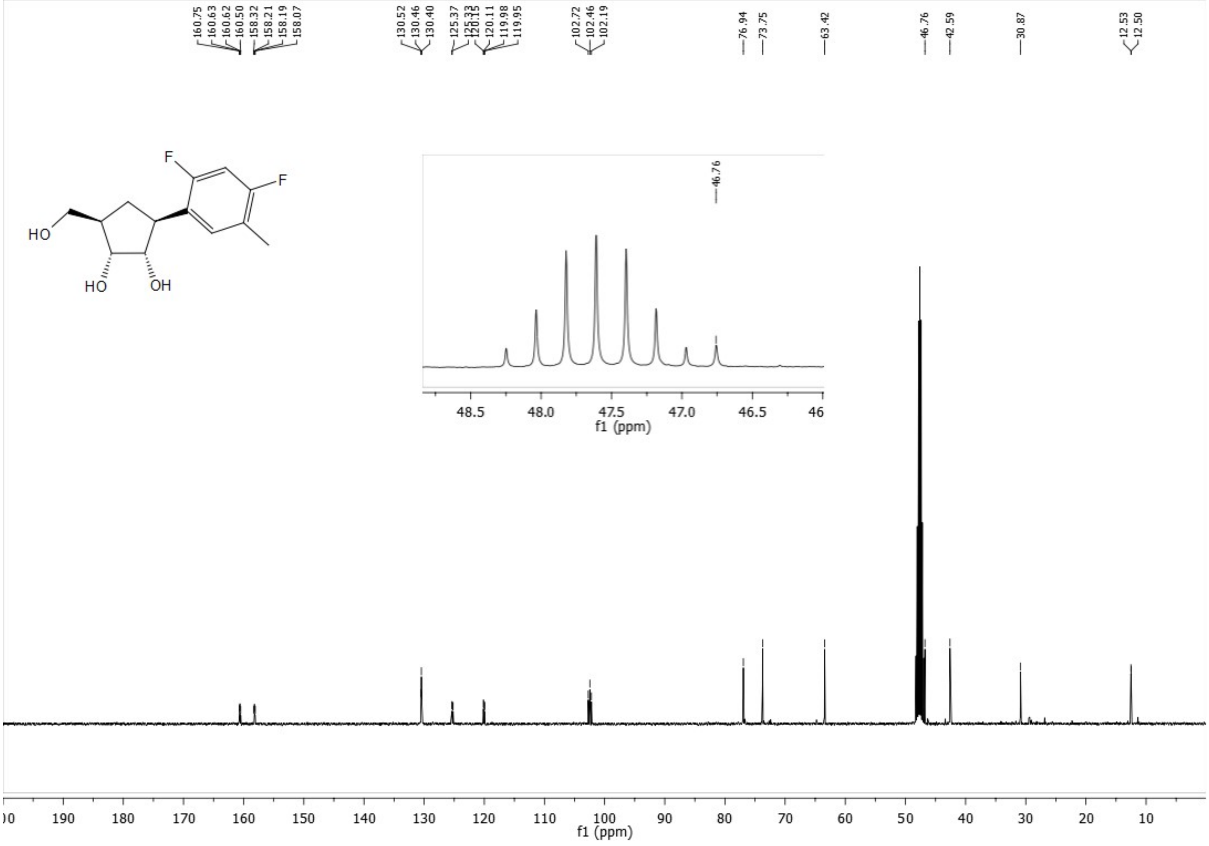

(1S,2R,3R,5S)-3-(hydroxymethyl)-5-(pyridin-3-yl)cyclopentane-1,2-diol (11j)

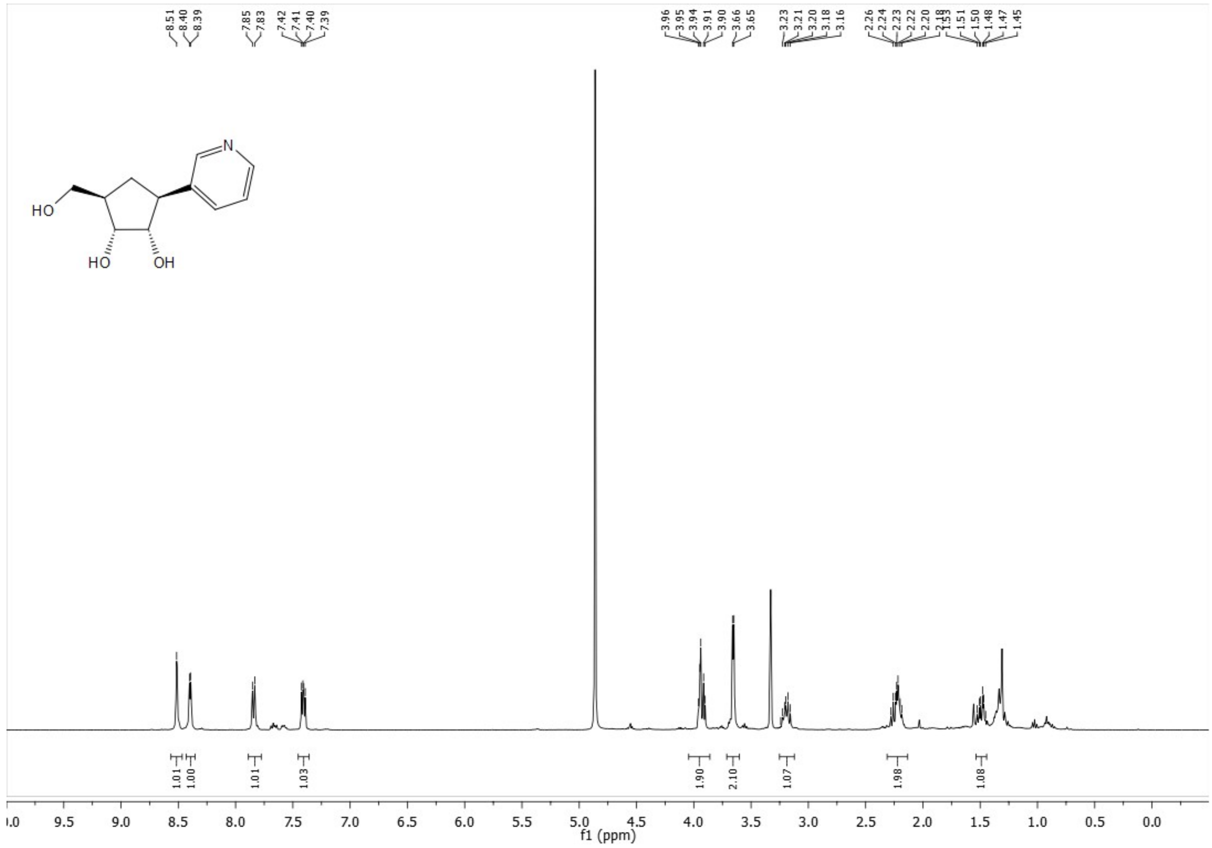

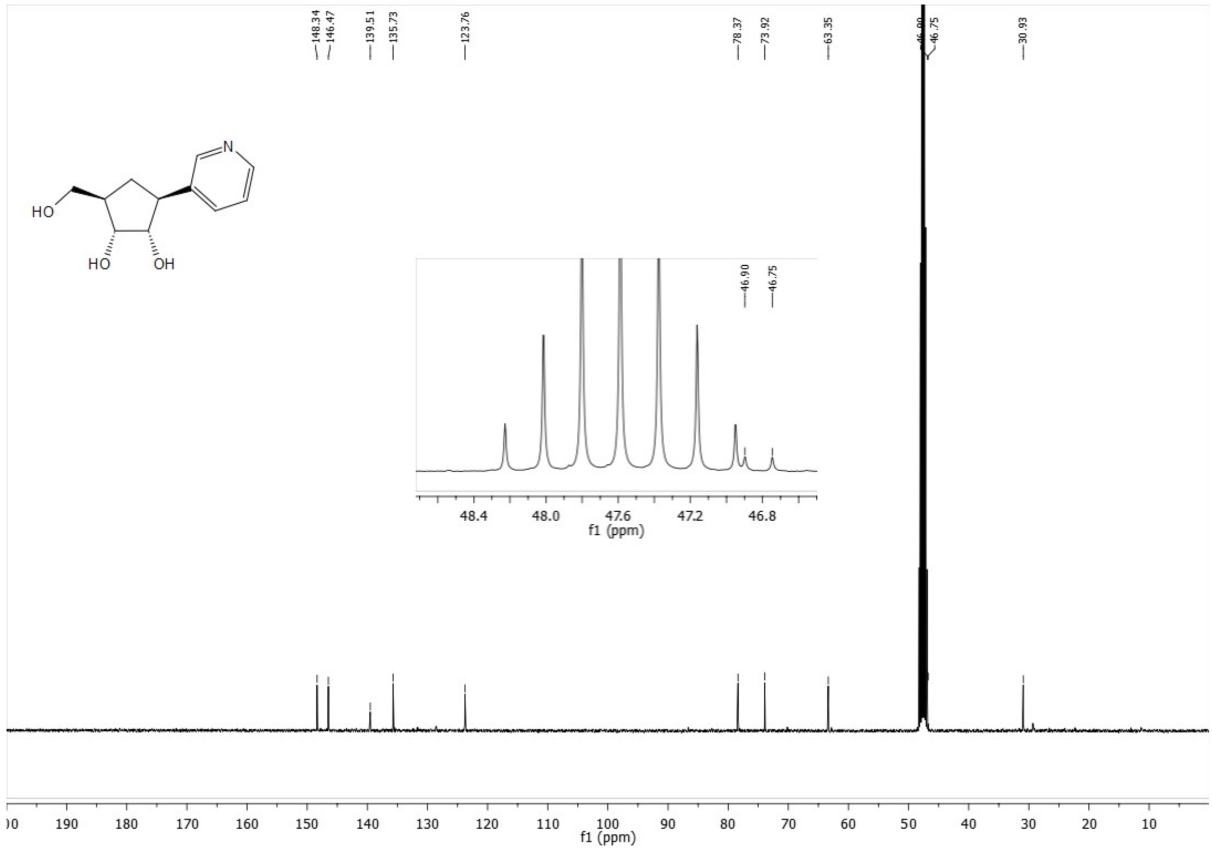

(1R,2S,3S,5R)-3-(6-fluoropyridin-3-yl)-5-(hydroxymethyl)cyclopentane-1,2-diol (11k)

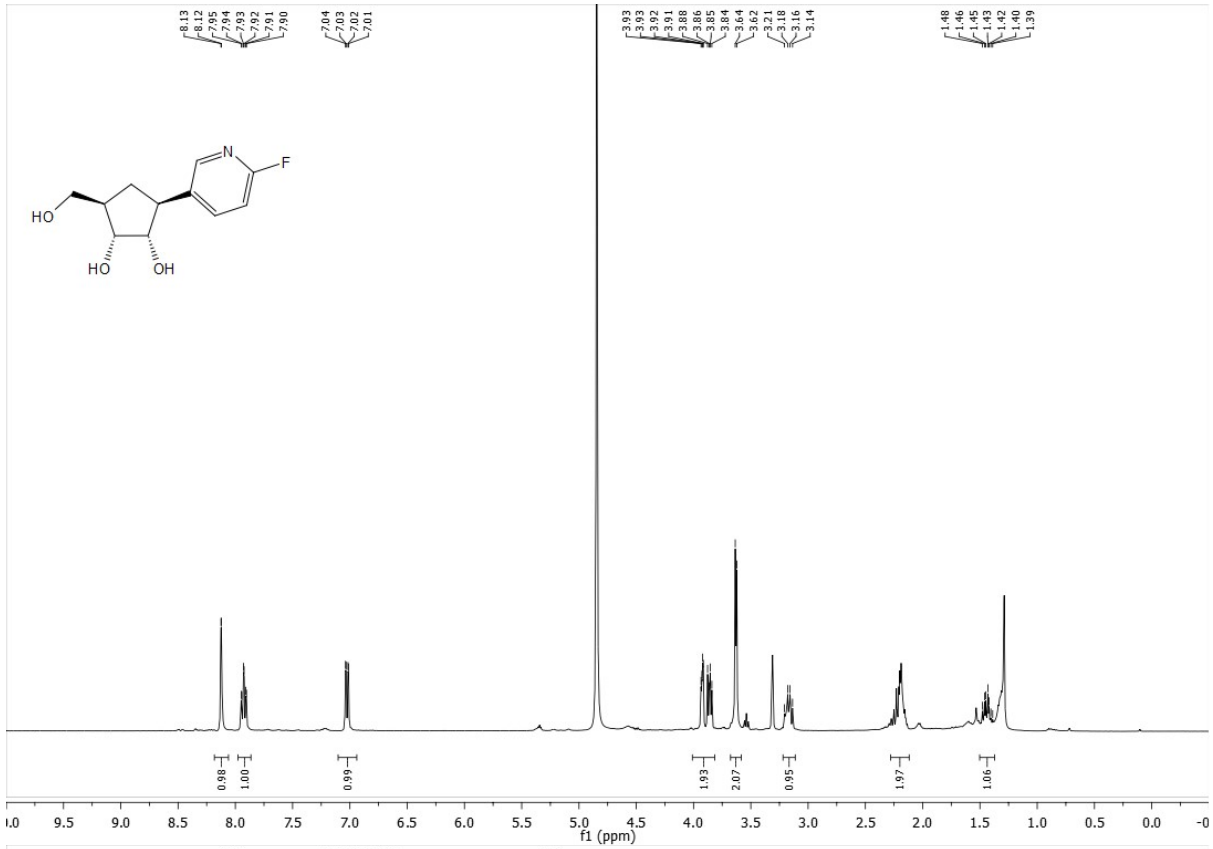

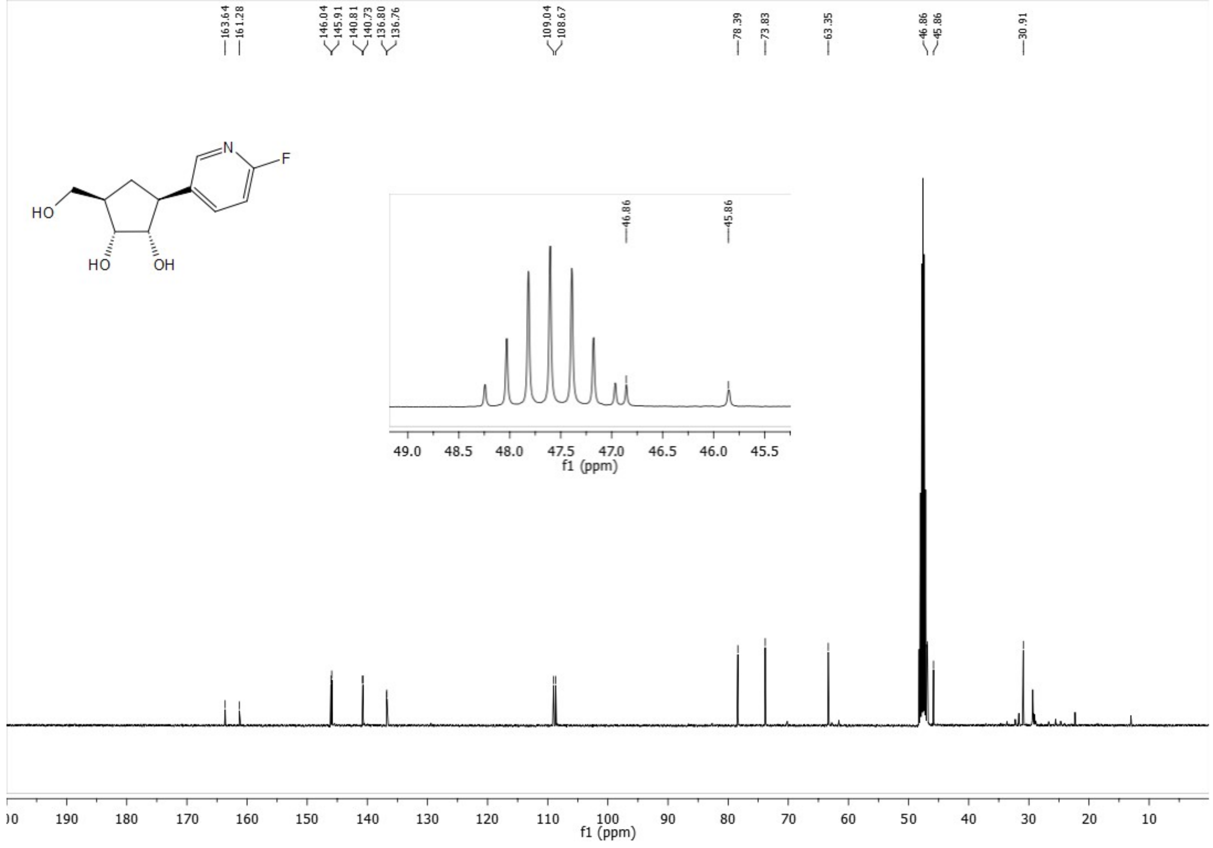

4-((1S,2S,3R,4R)-2,3-dihydroxy-4-(hydroxymethyl)cyclopentyl)benzonitrile (11l)

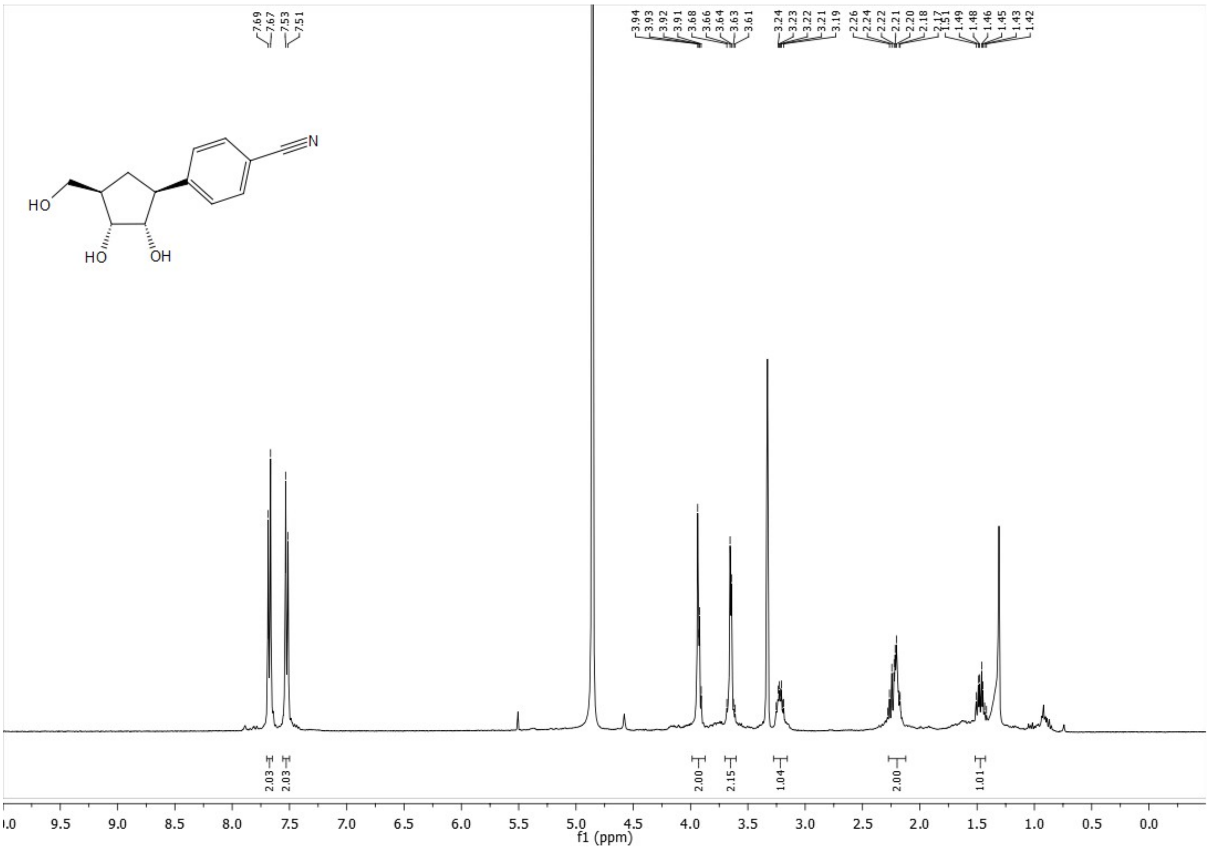

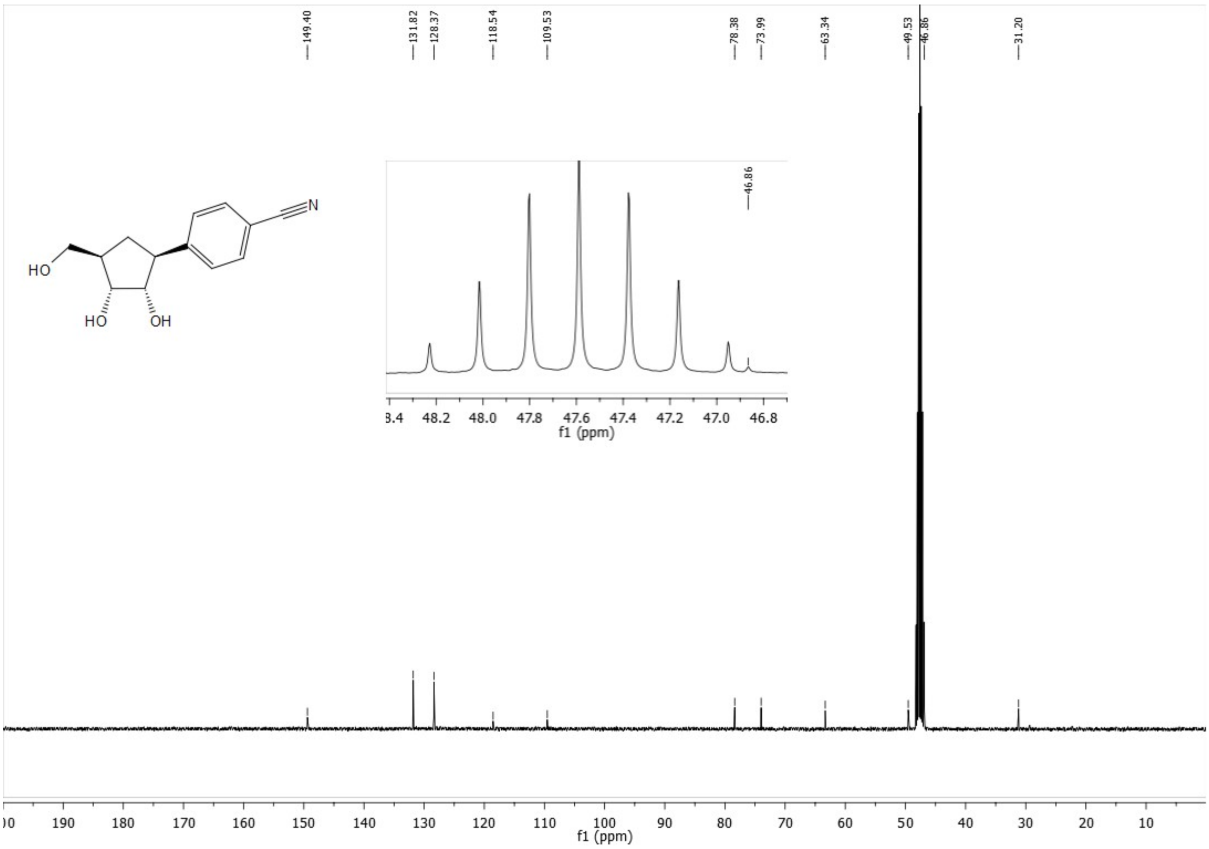

3D view (JSmol viewer) of best conformer of compound 2 calculated by Cheminfo

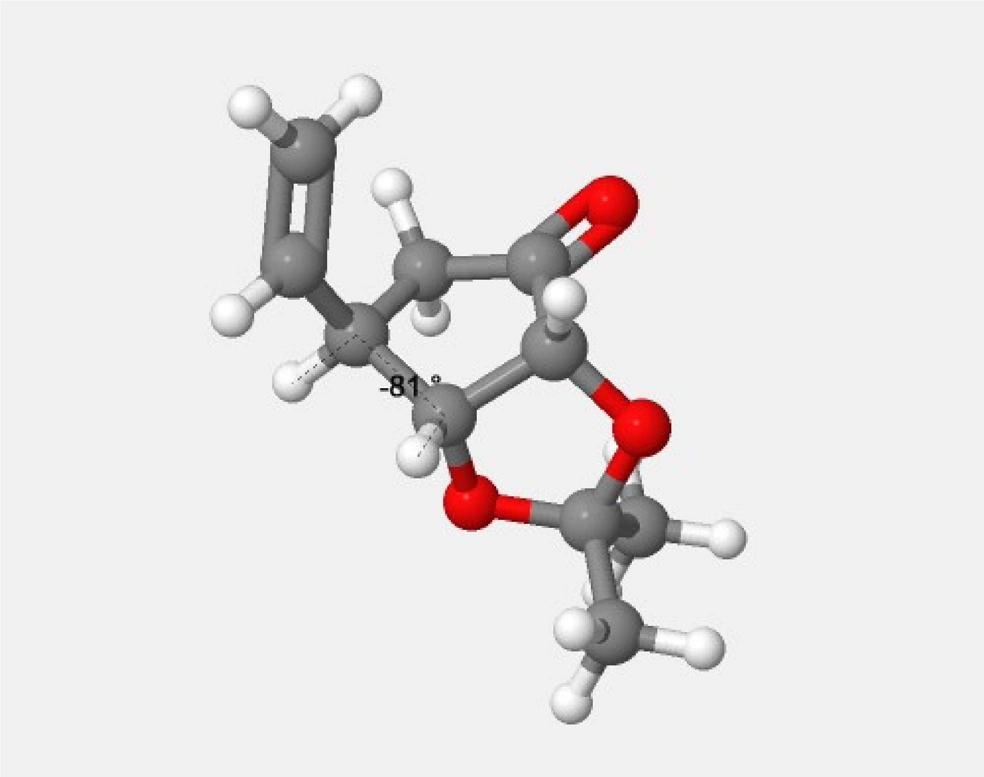

(from http://www.cheminfo.org/flavor/scgc/3D_models/2D_to_3D/index.html)

3D view (JSmol viewer) of best conformer of compound 3 calculated by Cheminfo

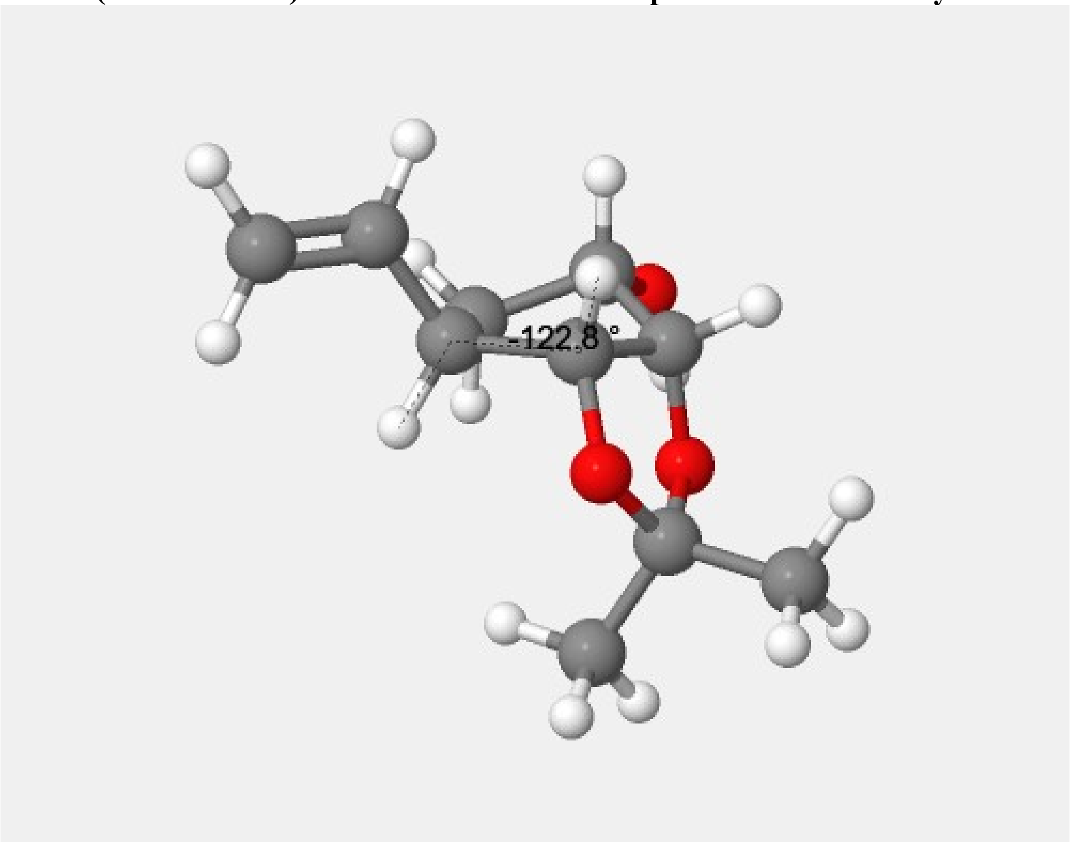

(from http://www.cheminfo.org/flavor/scgc/3D_models/2D_to_3D/index.html)
